# Asymmetric Synthesis and Biological Evaluation of 1,3‐ and 1,4‐Disubstituted Isoquinoline‐Containing Lipoxin A_4_ Analogues

**DOI:** 10.1002/chem.202502091

**Published:** 2025-08-27

**Authors:** Denise Moran, Monica de Gaetano, Braden Millar, Catherine Godson, Patrick J. Guiry

**Affiliations:** ^1^ Centre for Synthesis & Chemical Biology School of Chemistry University College Dublin Belfield Dublin 4 Ireland; ^2^ UCD Conway Institute & School of Biomolecular & Biomedical Science University College Dublin Belfield Dublin 4 Ireland; ^3^ UCD Conway Institute & School of Medicine University College Dublin Belfield Dublin 4 Ireland

**Keywords:** asymmetric synthesis, Heck coupling, inflammation, lipoxin analogues

## Abstract

The resolution of inflammation is increasingly recognized as an active, highly regulated process essential for restoring tissue homeostasis following immune activation. Lipoxin‐A_4_ (LXA_4_), an endogenous specialized pro‐resolving mediator (SPM), plays a central role in this process through activation of the ALX/FPR2 receptor. However, its clinical application is limited by rapid metabolic degradation and poor in vivo stability. In this study, we report the design, asymmetric synthesis, and biological evaluation of novel 1,3‐ and 1,4‐disubstituted isoquinoline‐based analogues of LXA_4_ (Isoq‐sLXms), designed to enhance metabolic stability. The synthetic route employed a palladium‐catalyzed Heck cross‐coupling and Ru(II)‐catalyzed asymmetric transfer hydrogenation, affording diastereomerically pure compounds. Biological assessment in THP‐1 LUCIA monocytes demonstrated that several analogues, particularly (*1R*)‐**8**, significantly attenuate lipopolysaccharide (LPS)‐induced NF‐κB activation and downstream pro‐inflammatory cytokine secretion, including IL‐6, IL‐1β, and TNF. Functional assays using ALX/FPR2‐transfected HEK‐293 cells revealed that (*1R*)‐**8** acts as a partial agonist, supporting its role in engaging pro‐resolving signaling mechanisms. Safety profiling confirmed low cytotoxicity across physiologically relevant concentrations. These findings demonstrate that isoquinoline‐based LXA_4_ mimetics retain and enhance key pro‐resolving bioactivities while offering improved stability and receptor selectivity. (*1R*)‐**8** emerges as a promising lead compound for the development of resolution‐directed therapeutics in the context of chronic inflammation.

## Graphical abstract

(1*R*)‐ and (1*S*)‐Epimers of novel isoquinoline‐derived SPMs were prepared, with a Pd‐catalyzed Heck reaction and a Ru(II)‐catalyzed asymmetric transfer hydrogenation as the key synthetic steps. Biological assessment demonstrated that several analogues significantly attenuate lipopolysaccharide‐induced NF‐κB activation and downstream pro‐inflammatory cytokine secretion, including IL‐6, IL‐1β, and TNF.

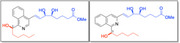

**Abbreviations**
GPCR: G protein‐coupled receptor
IL: interleukin
TLC: thin layer chromatography

## 1 Introduction

**Scheme 1:**
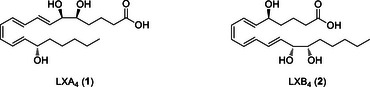
Native LXs LXA4 and LXB4.

The principal strategy for the treatment of inflammatory diseases has involved inhibiting the biosynthesis or the activity of pro‐inflammatory mediators. In particular, anti‐inflammatory and immune‐suppressive effects of glucocorticoids have been of paramount importance in controlling acute inflammation and autoimmune diseases (including rheumatoid arthritis, inflammatory bowel disease, multiple sclerosis, psoriasis, and eczema). However, their clinical efficacy is compromised by the metabolic effects of long‐term treatment, which include osteoporosis, hypertension, dyslipidemia, and insulin resistance/type 2 diabetes mellitus.^[^ ^1^ ^]^ To avoid the immune‐suppressive adverse side effects of this approach, more recent alternatives have looked at actively promoting the resolution of inflammation.^[^ ^2^ ^]^ Lipoxins (LXs) are a class of lipid mediators, which were discovered to act as endogenous anti‐inflammatory or pro‐resolving mediators.^[^ ^3^ ^]^ The two main types, LXA_4_ and LXB_4_ (Scheme 1), are derived from arachidonic acid and are produced at the site of inflammation, where they play a key role in potently suppressing inflammation.^[^ ^4^ ^]^ They act as stop signals for inflammation by regulating the chemotaxis, adhesion, and transmigration of polymorphonuclear leukocytes (PMNs).^[^ ^5^, ^6^ ^]^ LXA_4_ is believed to bind to a GPCR (namely FPR2) and induces nonphlogistic apoptosis of PMNs by macrophages.^[^ ^7^, ^8^ ^]^ Soon after their discovery in 1984, many pathways to the synthesis of LX were described.^[^ ^9^, ^10^, ^11^ ^]^ LXs are part of a cohort of molecules, collectively referred to as specialized pro‐resolving mediators (SMPs), that play an active role in the resolution phase of inflammation. Recent advances in the chemistry and biology of specialized pro‐resolving mediators (SPMs) have been reviewed by us.^[^ ^12^, ^13^, ^14^ ^]^

**Scheme 2:**
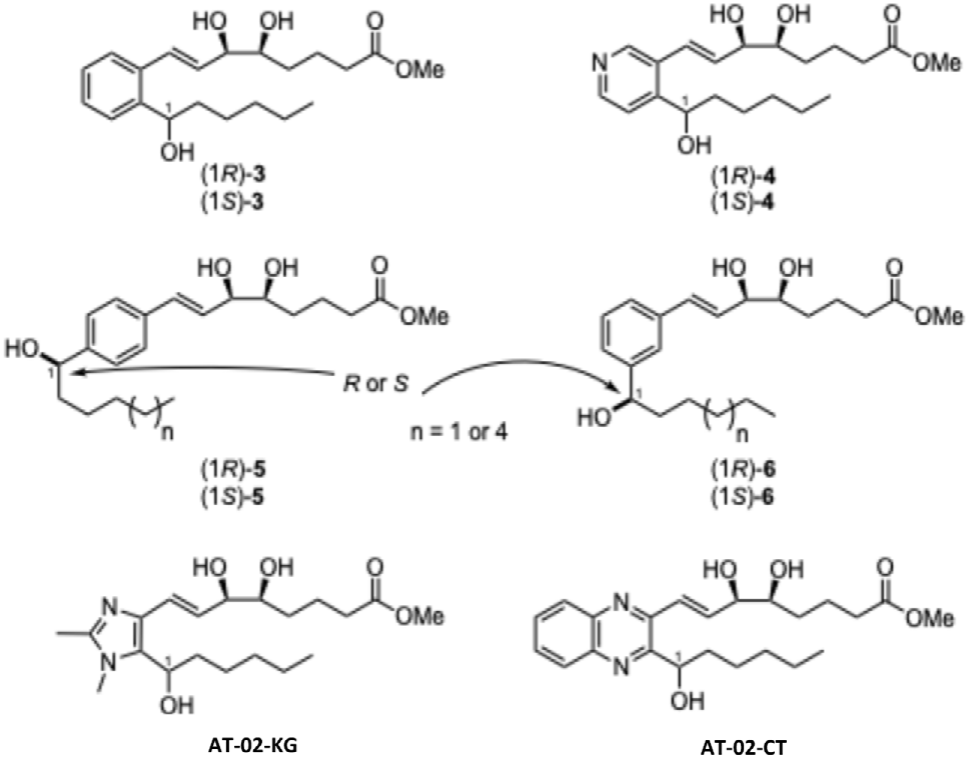
A selection of our previously reported synthetic LXA_4_ analogues.

The therapeutic potential of the native LXs is greatly hindered by their rapid metabolism in vivo, as they are prone to C_15_ and C_20_ oxidation and reduction at the C_13_‐C_14_ double bond.^[^ ^15^, ^16^ ^]^ A variety of strategies have been employed to circumvent these problems, including structural modifications of the C_15–20_ chain by Serhan and Petasis,^[^ ^17^, ^18^, ^19^ ^]^ while Guildford designed β‐oxidation‐resistant analogues by modifying the C_1–8_ unit.^[^ ^20^ ^]^ We attempted to stabilize the triene core of LXA_4_ with an aromatic ring, namely benzo‐LXA_4_ analogues (*1R/S*)‐**3** (Scheme 2). The stereoselective synthesis of both benzylic epimers was reported, and pleasingly, it was found that they induced a significant increase in phagocytosis of PMNs by F‐actin rearrangement, wherein the analogues displayed greater potency than the native LXA_4_.^[^ ^21^ ^]^ Subsequent studies discovered that benzo‐LXA_4_ can inhibit the production of the pro‐inflammatory leukotriene LTB_4_,^[^ ^22^ ^]^ can decrease obesity‐induced adipose inflammation,^[^ ^23^, ^24^ ^]^ and can attenuate renal fibrosis by inducing let‐7c and suppressing TGFβR1.^[^ ^25^ ^]^ Benzo‐LXA_4_ was found to protect against diabetes‐induced vascular complications in a similar manner to native LXA_4_, albeit at a lower dose. Importantly, our study demonstrated that LXA_4_ and benzo‐LXA_4_ suppress fibrotic gene expression by negatively regulating the early growth response factor (EGR‐1) transcription factor network, a complex dysregulated in human renal fibrosis.^[^ ^26^ ^]^

Petasis was the first to investigate the conformational flexibility of the ALX/FPR2 receptor by varying the angular orientation of the upper (C1‐C8) and the lower (C13‐C20) chains. *Ortho‐* and *meta‐*benzo analogues were synthesized and were found to have similar efficacy in blocking PMN infiltration in zymosan A‐induced peritonitis.^[^ ^27^ ^]^ As an extension of our benzo‐LXA_4_ work, we performed a stereoselective synthesis of novel Lipoxin A_4_ analogues (*1R/S*)‐**5**/**6**, in which we varied the substitution pattern around the benzene ring to afford analogues mimicking potential conformers of the native LXA_4_ that may adapt in vivo, and our preliminary results demonstrate the anti‐inflammatory potential of these novel LXA_4_ analogues.^[^ ^28^ ^]^

In addition, we synthesized pyridine‐LXA_4_ analogues (*1R/S*)‐**4** to probe the potential of using a heteroaromatic bioisostere of benzene, which has seen much success in medicinal chemistry: the two diastereomers of pyridine‐LXA_4_ significantly enhanced PMN phagocytosis and also suppressed the pro‐inflammatory cytokines IL‐12p40 and IL‐1β.^[^ ^29^ ^]^ Other nitrogen‐containing heterocycles such as imidazole, quinoxaline, and benzothiophene were incorporated.^[^ ^30^, ^31^, ^32^ ^]^ Two of the most successful analogues to date are the patented imidazole‐ and quinoxaline‐containing analogues, otherwise known as AT‐01‐KG and AT‐02‐CT, respectively. Both have been extensively screened for the anti‐inflammatory and pro‐resolving functions in comparison to endogenous LXA_4_ (**1**).

**Scheme 3:**
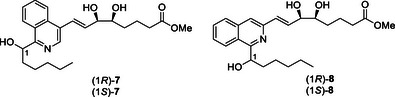
Target isoquinoline LXA_4_ analogues.

Herein we report the development of further heteroaromatic LXA_4_ analogues, namely isoquinoline‐containing (Isoq‐LXA_4_), whilst also probing the importance of the angular conformation of the upper and lower side chains. The initial target was the 1,4‐disubstituted isoquinoline analogues (*1R*/*S*)‐**7,** where the arrangement of the side chains mimics the *para‐*benzo analogue **6** (Scheme 3). Isoquinoline is an electron‐deficient system and the nitrogen in the ring may allow additional binding interactions at the ALX receptor. We also investigated the synthesis of 1,3‐disubstituted isoquinoline analogues (*1R*/*S*)‐**8** to mimic the *meta‐*benzo analogue **5**.^[^ ^33^ ^]^ This choice of substitution patterns was also influenced by the availability of the required starting materials for the proposed synthesis of the target analogues **7** and **8**, relative to that required to prepare the corresponding 3,4‐disubstituted isoquinoline analogue.

**Scheme 4:**
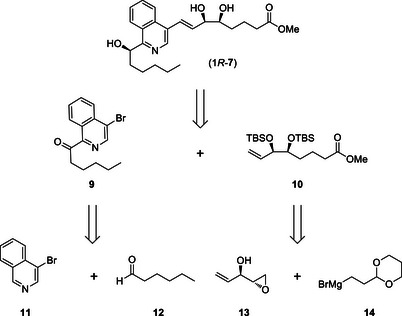
Retrosynthetic analysis of 1,4‐isoquinoline LXA_4_ (*1R*)‐**7**.

The retrosynthetic analysis of the 1,4‐isoquinoline analogue (*1R*)‐**7** was envisaged to involve a desilylation of the dihydroxy groups preceded by an asymmetric reduction of a ketone to access the (*1R*)‐alcohol (Scheme 4). The cross‐coupling of the heteroaryl component **9** to the upper chain **10** was proposed to proceed via a palladium‐catalyzed Heck reaction, while the protected upper chain **10** would be derived from a six‐step route that includes the addition of Grignard reagent **14** to the enantiopure epoxide **13**.

## 2 Synthesis of Isoq‐LXA_4_ Analogues

**Scheme 5:**
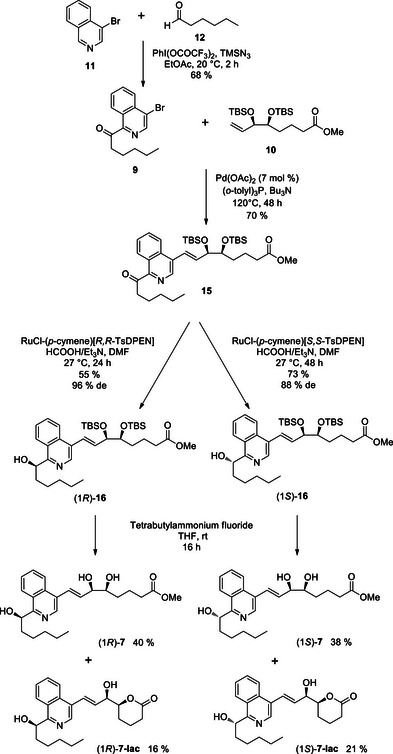
Synthesis of 1,4‐isoquinoline LXA_4_ analogues.

The six‐step synthesis of the upper chain **10** is detailed in our previous reports involving a Sharpless asymmetric epoxidation and a one‐pot deprotection‐esterification procedure catalyzed by ZrCl_4_.^[^ ^21^ ^]^ The synthesis of the heteroaryl ketone **9** was initially attempted using Knochel's mixed Mg/Li amide base, TMPMgCl·LiCl, to selectively metalate carbon‐1 of 4‐bromoisoquinoline, but the desired alcohol was not obtained upon quenching with hexanal. Instead, the synthesis was based on the cross‐dehydrogenative coupling (CDC) protocol by Antonchick, who showed that a hypervalent iodine species can act as a metal‐free, oxidizing agent for the coupling of hetero‐aryls with aldehydes at ambient temperature.^[^ ^34^ ^]^ Isoquinoline was chosen as the model heteroaryl for this methodology and gave C‐H functionalization solely at carbon‐1. We discovered the protocol was also suitable for halo‐isoquinolines whereby 4‐bromoisoquinoline and hexanal were coupled using (bis(trifluoroacetoxy)iodo)benzene (PhI(OCOCF_3_)_2_) with TMS azide as the additive at room temperature to give the desired ketone **9** in a 68 % yield (Scheme 5).

With the upper chain **10** and heteroaryl bromide **9** in hand, the Heck coupling reaction was investigated. The conditions which had worked well for the benzo analogues were chosen using palladium (II) acetate and tri‐*o*‐tolylphosphine with tributylamine as both the base and the solvent for the reaction.^[^ ^21^ ^]^ These conditions successfully formed the new carbon‐carbon bond of intermediate **15** in a 70 % yield. The next step in the synthesis required the asymmetric reduction of the ketone to give both epimers at this position to mimic the native LXA_4_ and C_15_‐epi‐LXA_4_. The initial method of choice was using the proline‐based Corey‐Bakshi‐Shibata (CBS) catalyst in the presence of catecholborane.^[^ ^35^ ^]^ However, no reduction was observed. Corey has shown that such nitrogen‐containing aromatics can be unsuitable for CBS‐catalyzed reductions if the ketone is *ortho* to the nitrogen, as the catecholborane can coordinate to both the nitrogen and the oxygen of the substrate and deliver hydride nonenantioselectively.^[^ ^36^ ^]^ We proved this by performing a CBS‐catalyzed reduction on ketone **9** and obtaining an *ee* of only 13 %. Other attempts investigated were using Brown's DIP chloride reagent and direct hydrogenation by Noyori's Ru‐BINAP complex with varied hydrogen pressure, but these were unsuccessful in reducing the carbonyl group of **9**. As the reduction of the coupled product was proving difficult, it was considered to perform the reduction prior to Heck coupling. To test the suitability of this route, the aryl ketone **9** was racemically reduced and subjected to the same Heck coupling conditions as in Scheme 2 with the upper chain **10**. However, this produced mainly debrominated starting material. However, we were delighted to discover that transfer hydrogenation with a Ru‐diamine complex and a formic acid/trimethylamine mixture could induce high diastereoselectivity to yield the (*1R*)‐**16** and (*1S*)‐**16** epimers in 96 % and 88 % *de*, respectively.^[^ ^37^ ^]^ The final step in the synthesis was the removal of the TBS protecting groups, which was performed using tetrabutylammonium fluoride in THF. The deprotection led to a mixture of the desired diols (*1R*)‐**7**, (*1S*)‐**7**, and lactones (*1R*)‐**7‐lac**, (*1S*)‐**7‐lac**.

**Scheme 6:**
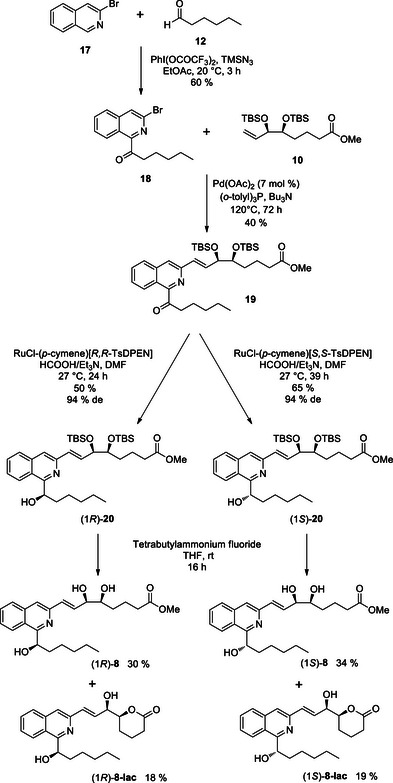
Synthesis of 1,3‐isoquinoline LXA_4_ analogues.

The preparation of the 1,3‐isoquinoline LXA_4_ analogues followed a similar route as described for the 1,4‐analogues (Scheme 6). The ketone coupling partner **18** was accessed by C_1_ ‐functionalization of 3‐bromoisoquinoline **17** with hexanal under the CDC conditions previously employed. The chosen Heck reaction conditions furnished the desired *trans*‐alkene **19,** albeit in a modest yield of 40 %. We were pleased to observe that the Ru‐catalyzed transfer hydrogenation protocol was successful for ketone reduction of **19** to afford the alcohols (*1R*)‐**20** and (*1S*)‐**20** in good yields and excellent levels of diastereoselectivity, reaching 94 % *de* in both epimers. The final desilylation step again produced a mixture of the epimeric LXA_4_ analogues, (*1R/S*)‐**8,** and the lactonized compounds (*1R/S*)‐**8‐lac** in an approximately 2:1 ratio.

With these novel LXA_4_ analogues and their lactone derivatives in hand, the biological evaluation was conducted on six of the eight analogues to investigate their anti‐inflammatory activity. In addition, native LXA_4_ **1** and benzo‐LXA_4_ (*1S*)‐**3** were tested concurrently to allow for direct comparisons of their efficacy and potency.

## 3 Biological Evaluation of Isoq‐LXA_4_ Analogues

THP‐1 LUCIAs, human leukaemia‐derived monocytes with a stably transfected NF‐κB luciferase promoter reporter, exposed to an exogenous nonsterile infectious *stimulus* (50 ng/mL LPS) were used to screen the anti‐inflammatory capabilities of these isoquinoline analogues of LXA_4_ (Isoq‐sLXms), as previously described.^[^ ^30^, ^31^ ^]^ A rational design of the eight compounds initially synthesized is displayed in Supplementary Figure 1.The six compounds pure enough for biological evaluation [specifically, (*1R*)‐**7**, (*1S*)‐**7**, (*1S*)‐**7‐lac**, (*1R*)‐**8**, (*1R*)‐**8‐lac**, and (*1S*)‐**8**] were screened to evaluate their inflammation attenuating capabilities (Sections 3.1 and 3.2), cytotoxicity profiling (Section 3.3), and FPR2 receptor activation (Section 3.4), in response to a pro‐inflammatory stimulus (LPS), with data generated from sLXms‐treated samples expressed as percentages of baseline behavior of the cells.

### 3.1 Isoq‐sLXms Attenuates NF‐κB Activity in Human Monocytes

**Figure 1:**
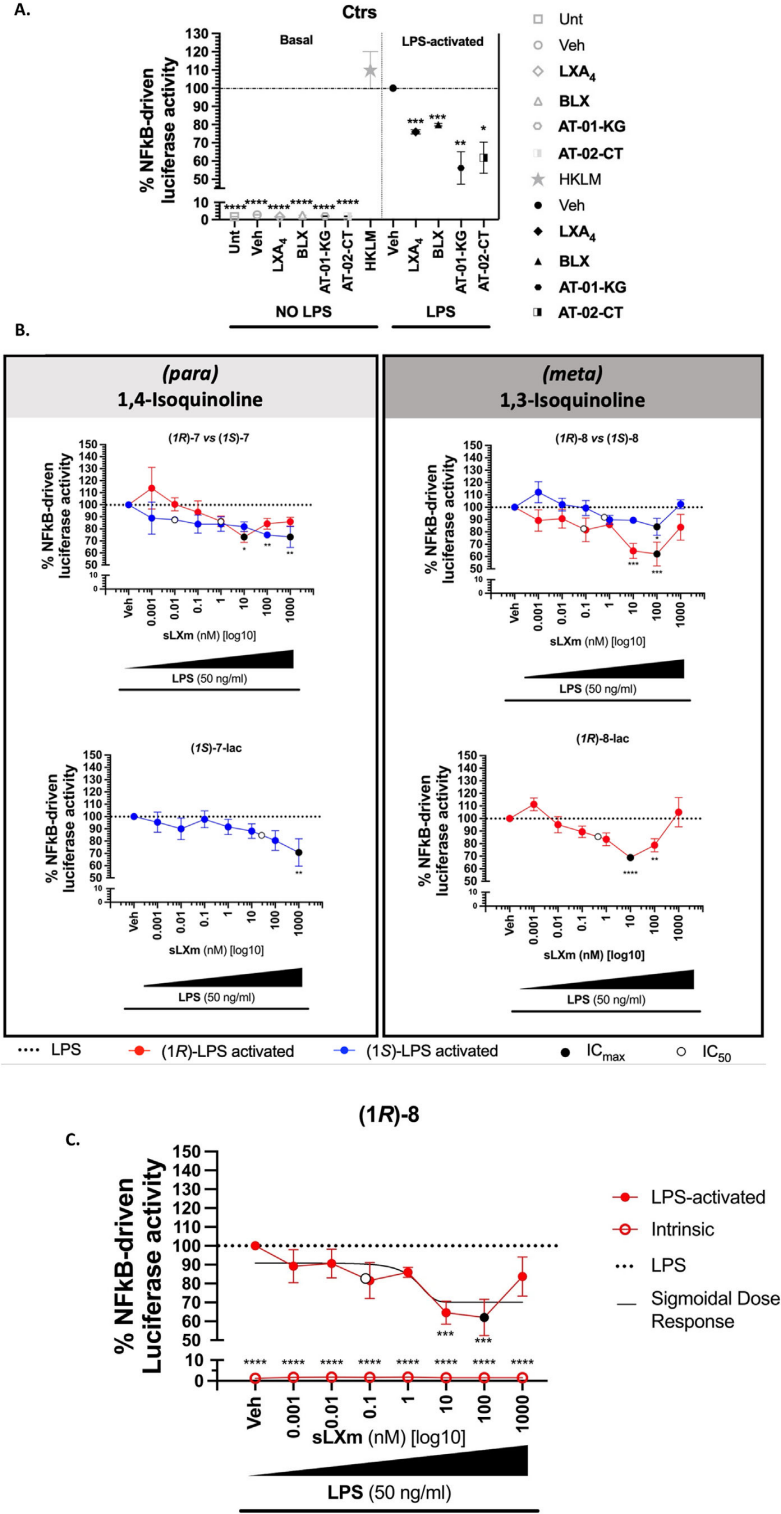
Effect of Isoq Mimetics on LPS‐induced NF‐kB‐driven Luciferase Activity in THP‐1 LUCIA monocytes. 1 x 10^5^ THP‐1 LUCIA monocytes were pre‐treated for 30 min. with increasing concentrations of the isoquinoline‐containing sLXms (10^−12^ M / 1 pM – 10^−6^ M / 1000 nM), vehicle, or appropriate controls. This was followed by the addition of 50 ng/mL LPS (see luciferase activity in the absence of LPS in supplementary Figure 2) for 24 hours. Cell supernatants were collected, and NF‐kB luciferase activity was assayed. Data are expressed as a % ± SEM (n = 3) of NF‐kB‐driven luciferase activity as normalized luminescence unit relative to an LPS‐induced response in the absence of sLXms. A) Single‐point analysis of the internal controls (adapted from de Gaetano et al. 2021). B) Concentration‐response curve comparing the epimers of the eight sLXms. [Note: (1*R)*‐**7**‐lac and (1*S*)‐**8**‐lac were not pure enough for biological evaluation and hence are not included in the figure]. (C) Luciferase activity of the lead compound (1R)‐8 showing LPS‐induced and basal effects on luciferase activity, and a best‐fitting curve of the compound. Statistical analyses were performed using Student's unpaired two‐tailed t‐test of the tested compounds versus LPS and one‐way ANOVA with multiple comparisons (*p < 0.05; **p < 0.01; ***p < 0.001; ****p,0.0001).

As expected, both the LPS and HKLM *stimuli* yielded NF‐κB activity to the cell, evoking a luminescence signal. Hence, intrinsic LPS‐induced activity of untreated‐ or vehicle‐treated cells was set to 100% for analysis of the individual effects of the six Isoq‐sLXms. As previously shown, LXA_4_, AT‐01‐KG, and AT‐02‐CT, respectively, reduced NF‐κB activity by 24% (±1%, IC_max_ 100 nM, IC_50_ 0.1 nM), 44% (±9%, IC_max_ 1 pM, IC_50_ < 1 pM), and 38% (±9%, IC_max_ 10 nM, IC_50_ 0.1 nM),^[^ ^31^ ^]^ setting a benchmark for the performance of the Isoq‐sLXms currently tested (Figure 1A).

As shown in Figure 1B, when examining the effects Isoq‐sLXms had on NF‐κB activity, their relative luminescence via the luciferase reporter was recorded as a percentage of HKLM activation. Overall, substantial and significant reduction in NF‐κB activity was recorded across all Isoq‐sLXms, with *R*‐epimers and compounds lacking a lactone ring appearing dominant in their effects.

The (*S*)‐epimers appeared less efficacious, achieving maximal reduction at the highest concentrations (1 µM), although (*1S*)‐**7** has the lowest IC_50_ at 1 pM (with a shallow concentration‐response). (*1S*)‐**7** and (*1S*)‐**7‐lac** both possess an IC_max_ of 1 µM, reducing NF‐κB activity by 26.8% (±8.7%, IC_50_ 1 µM, p < 0.01) and 29.2% (±11.2%, IC_50_ 20 nM, p < 0.05), respectively. On the other hand, (*R*)‐epimers, namely (*1R*)‐**7**, (*1R*)‐**8**, and (*1R*)‐**8‐lac**, reduced NF‐κB activity to a greater extent. In particular, (*1R*)‐**7** and (*1R*)‐**8‐lac** achieved a reduction of 26.8% (±4.54%, IC_50_ 0.9 nM, p < 0.05) and 31.1% (±2.6%, IC_50_ ∼0.8 nM, p < 0.0001), respectively, with an identical IC_max_ of 10 nM (Figure 1b).

(*1R*)‐**8** proved as the most potent compound for reduction of NF‐κB activity yielding a 37.9% (±9.7%, IC_50_ ∼90 pM, p < 0.001) with an IC_max_ of 100 nM and similar effects at 10 nM (Figure 1c).

In summary, all ISOQ‐sLXms highlighted a significant ability to reduce the activation of the pro‐inflammatory pathway NF‐κB, with (*1R*)‐**8** being identified as the lead candidate by attenuating NF‐κB activity by approximately 40% in the presence of an LPS stimulus. In control experiments, where cells were not stimulated with LPS prior to the addition of the ISOQ‐sLXms, as expected, NF‐κB was not activated by any of the tested compounds (Supplementary Figure 2), indicating that none of the tested compounds induce per se NF‐κB activity, in the absence of an LPS stimulation (Supplementary Figure 2).

### 3.2 Isoq‐sLXms Attenuate NF‐κB Downstream Signaling by Reducing Pro‐Inflammatory Cytokine Release in Human Monocytes

NF‐κB activity has been well established as a driving force in the production and secretion of key pro‐inflammatory cytokines.^[^ ^38^, ^39^ ^]^ Out of the 6 compounds screened for NF‐κB activity, the 5 top‐performing mimetics were further tested for NF‐κB‐related downstream activities, specifically the pro‐inflammatory cytokine release, upon LPS stimulation. (*1S*)‐**8**, identified as the least effective in reducing NF‐κB activity in the initial assessment of the ISOQ‐sLXms, was therefore excluded from further testing (see Section 3.2). Due to the volume of data generated, we will here describe in detail solely the significant changes in secretion of the most pivotal and relevant targets, with more in‐depth analysis of (*1R*)‐**8**, our lead compound. All values are presented as a percentage of vehicle‐treated monocytes to indicate baseline changes in cytokine secretion.

All analogues tested reduced the secretion of pro‐inflammatory cytokines via attenuation of NF‐κB activity (Figure 2a). As previously reported, IL‐6 secretion was abolished by native LXA_4_ (**1**) (10 pM ‐ 100 nM) (p < 0.0001) with a maximal reduction of 97 ± 1%.^[^ ^30^ ^]^ In the Isoq‐sLXms testing, we observe a similar result as IL‐6 secretion is abolished from a baseline secretion of 3486.92 ± 259.78 pg/mL, with a maximal reduction of IL‐6 by 98.0 ± 1.9% (IC_max_ 0.01 nM, p < 0.0001) treating cells with (*1R*)‐**8‐lac**, and a minimal reduction by 85.6 ± 2.7% (IC_max_ 1 nM, p < 0.0001) using (*1S*)‐**7**.

Figure 2Effect of the sLXms on the Modulation of the Release of Pivotal Pro‐inflammatory Cytokines. 1 x 10^5^ THP‐1 LUCIA monocytes were pre‐treated for 30 min. with increasing concentrations of the isoquinoline‐containing sLXms (10^−12^ M [1 pM] – 10^−6 ^M [1,000 nM]), vehicle, or appropriate controls. This was followed by the addition of 50 ng/mL LPS. After 24 h, cell supernatant was collected, and cytokine release was assessed using a 7‐plex multiplex ELISA (IFN‐γ, IL‐10, IL‐12 p70, IL‐1β, IL‐6, IL‐8, and TNF). A) Comprehensive view of cytokine release from all sLXms. B) Comparative view of 3 key pro‐inflammatory cytokines across all sLXms. These data are expressed as a % Cytokine release ± SEM (n = 3) relative to vehicle control at 100%. [Note: (1S)‐8 showed inefficient reduction of NF‐*κ*B activity: therefore, excluded due to the extensive cost of the MSD plate and available space.].

**Figure:**
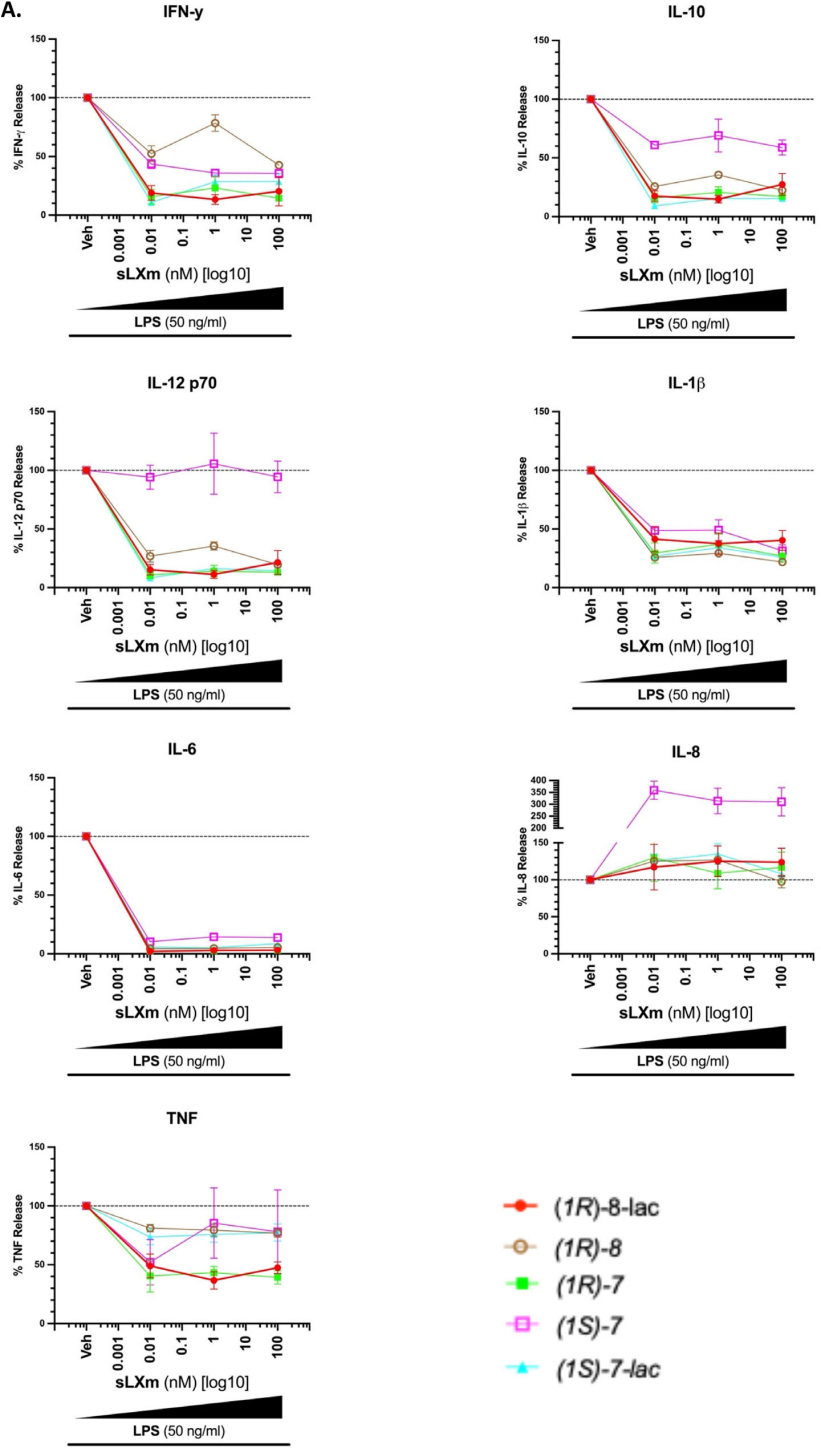

**Figure:**
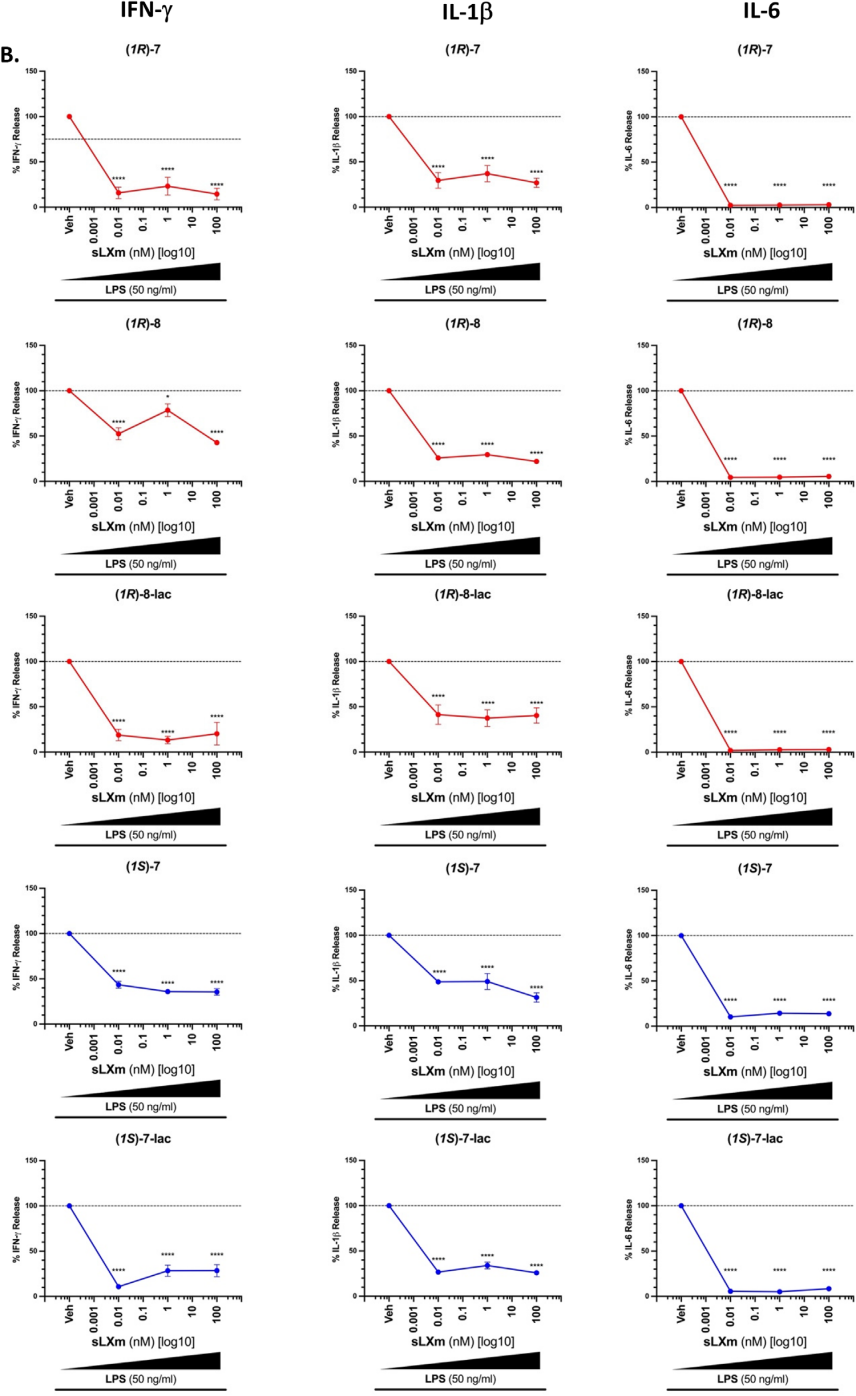

**Figure 3:**
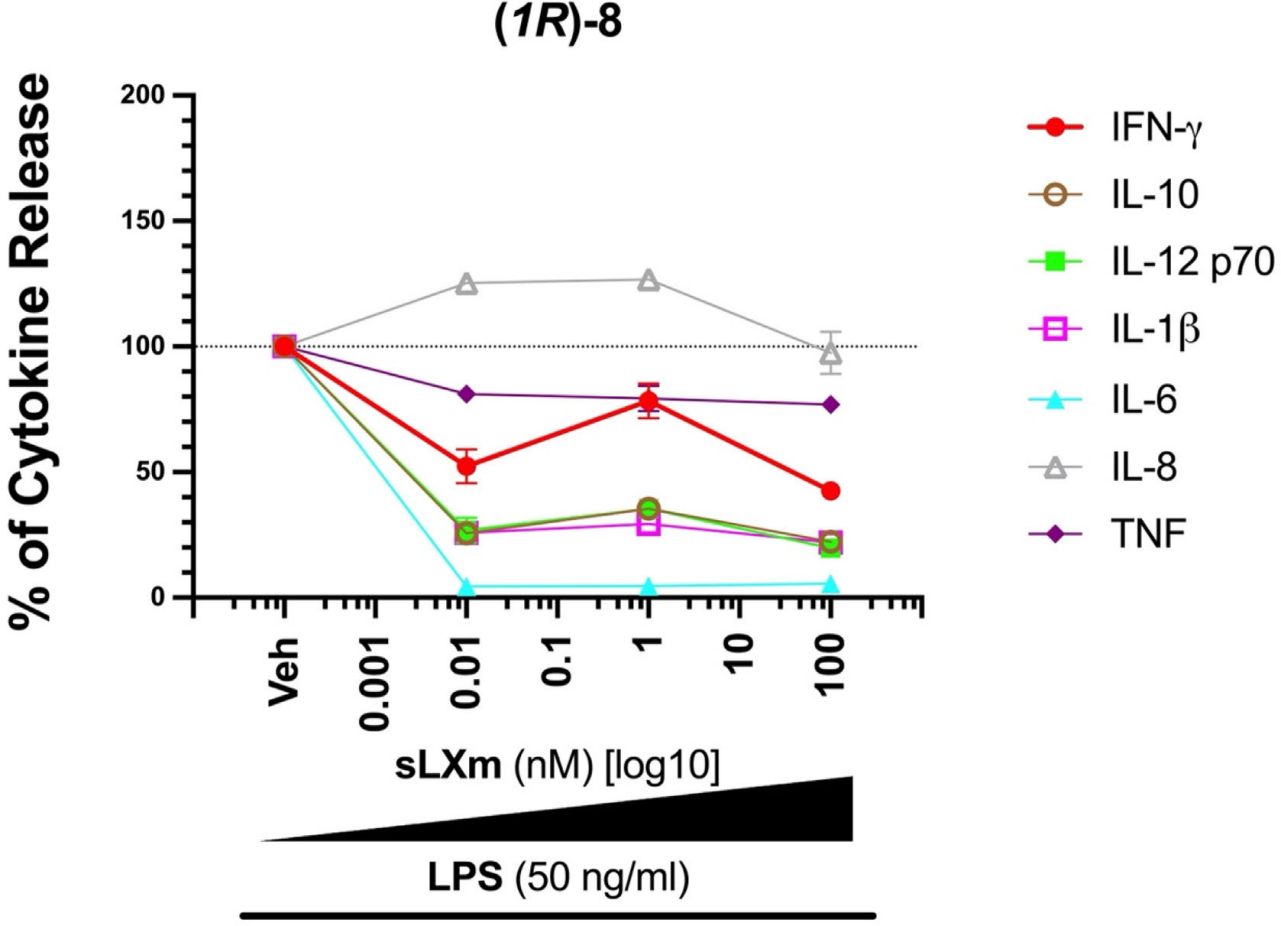
Anti‐inflammatory Effects of (1*R*)‐**8** on Pivotal Pro‐inflammatory Cytokines. A comprehensive overview of the capacity of (1*R*)‐**8** to attenuate inflammation by decreasing the secretion of the listed pivotal pro‐inflammatory cytokines in 1 x 10^5^ THP‐1 LUCIA monocytes pre‐treated for 30 minutes. with this mimetic before a 24‐hour exposure to 50 ng/mL LPS. These data are presented as a % release of each cytokine relative to its vehicle treatment at 10 pM, 1 nM, and 100 nM of (1*R*)‐**8**.

Between the two epimers tested for compound **7**, the (*1R*)‐epimer outperformed the (*1S*) one in reducing the production of these pivotal cytokines. For the different cytokines measured, the reduction observed using (*1S*)‐**7** was demonstrated by the following ICmax: 100 nM, by 64.5 ± 7.3%, p < 0.0001 for IFN‐ɣ; 100 nM, by 68.6 ± 9.1%, p < 0.0001 for IL‐1β; 0.01 nM, by 89.7 ± 2.5%, p < 0.0001 for IL‐6; 0.01 nM, by 47.9 ± 33.41%, p < 0.05 for TNF. On the other hand, the IC_max_ of cytokines measured for (*1R*)‐**7** were: 100 nM, by 85.6 ± 12.8%, p < 0.0001 for IFN‐ɣ; 100 nM, by 73.2 ± 10.23%, p < 0.0001 for IL‐1β; 0.01 nM, by 97.7 ± 1.9%, p < 0.0001 for IL‐6; 100 nM, by 60.8 ± 11.3%, p < 0.01 for TNF (Figure 2b). (*1R*)‐**8** displayed a maximal inhibitory activity concentration of 100 nM for all cytokines (except IL‐6 with an IC_max_ 0.01 nM), as seen in Figure 3: the observed reduction was by 57.4 ± 4.3%, p < 0.0001 for IFN‐ɣ; 77.7% ± 2.6%, p < 0.0001 for IL‐10; 80.4 ± 3.6%, p < 0.0001 for IL‐12 p70; 78.1 ± 2.3%, p < 0.0001 for IL‐1β; 96.7 ± 1.6%, p < 0.0001 for IL‐6; 23.2 ± 7%, not significant, for TNF. Therefore, (*1R*)‐**8** is suggested to be the lead candidate.

As previously observed with the native compound and also other sLXms,^[^ ^31^ ^]^ (*1S*)‐**7** also significantly increased IL‐8 secretion EC_max_ 10 pM by 259.1 ± 76.0%, p < 0.0001).

### 3.3 Isoq‐sLXm (1R)‐**8** Is a Partial ALX/FPR2 Receptor Agonist

ALX/FPR2 is a G‐coupled receptor which is activated by endogenous ligands such as LXA_4_. Here, the affinity for the SPM receptor was assessed using stably double transfected HEK‐293 overexpressing ALX/FPR2 receptor coupled to a G⍶_q_ subunit. The activation of this receptor triggers intracellular Ca^2+^ flux which can be fluorescently measured, thus, activation of the receptor via 1(*R*)‐**8** was determined by measuring Ca^2+^ flux.

**Figure 4:**
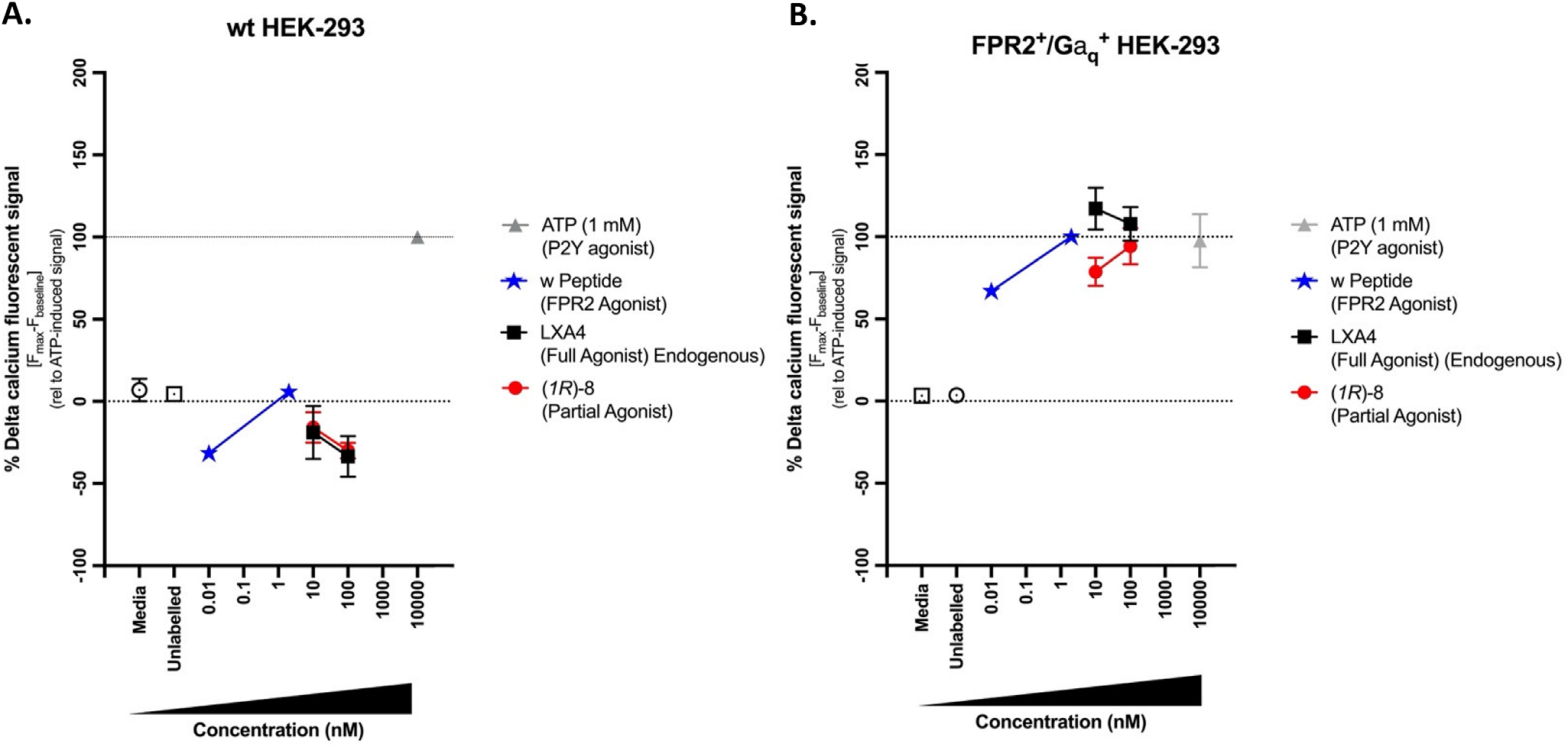
Effects of (1*R*)‐**8** on Intracellular Calcium Flux in A: wt HEK‐293 cells and B: Stably Transfected HEK‐293 versus Endogenous LXA_4_. Cells were cultured for 18 h prior to labelling with Fluo‐4 (37 °C, 1 h). Quantification of three independent experiments was carried out by calculating differential calcium signals measured at the baseline and at maximum peak. Data are expressed as % delta calcium‐induced fluorescent signal relative to the peak of a known full agonist (1) ± SEM (n = 3).

LXA_4_ (**1**), as previously documented,^[^ ^30^ ^]^ and (*1R*)‐**8** treated cells induced increased intracellular Ca^2+^ flux, achieving maximal activation at 10 nM and 100 nM, respectively. In wt HEK‐293 cells we see no activation of ALX/FPR2, as the only evidence of Ca^2+^ flux is seen in the ATP (Figure 4A). In transfected HEK‐293 cells we see maximal Ca^2+^ activation via ATP through the same P2Y receptor, but also full receptor activation by W peptide and LXA_4_ (**1**) (full endogenous agonist) (Figure 4B). (*1R*)‐**8,** on the other hand did not achieve the same receptor activation as the control or endogenous LXA_4_ (**1**). However, (*1R*)‐**8** did stimulate Ca^2+^ with similar potency to internal controls and LXA_4_ (**1**), but not as efficiently, thus suggesting that it is a partial agonist of FPR2.

### 3.4 Safety Profiling of Isoq sLXms

Lactate dehydrogenase (LDH) is an in vitro method of assessing cellular damage by measuring plasma‐membrane integrity that is usually associated with cell damage. Here, we measured LDH release from THP‐1 Lucia monocytes, treated with our sLXms, including a vehicle control (THP‐1 Lucias with no sLXms) to measure baseline LDH release. Maximal LDH release was measured in response to 2% Triton X‐100 cell lysis buffer treatment, and LDH release from sLXm‐treated cells was expressed as a percentage of maximal cell damage. “Safety windows” were also generated by setting limits based off of vehicle (negative control), LPS‐treatment (treatment control), and maximal LDH release (Triton X‐100).

As shown in Supplementary Figure 3, vehicle‐treated cells release 5x less LDH than Triton X‐100, very similar to untreated THP‐1 Lucias, showing again that the 0.1% EtOH solution in which the sLXms are reconstituted and diluted in, is not having a detrimental effect on the cells, as has been shown before.^[^ ^30^, ^31^ ^]^ LPS does double the release of LPS relative to untreated and vehicle controls, as previous reports have reported that LPS itself does induce LDH release, and hence LPS‐induced release of LDH has been set as the upper limit for degree of risk.

**Figure 5:**
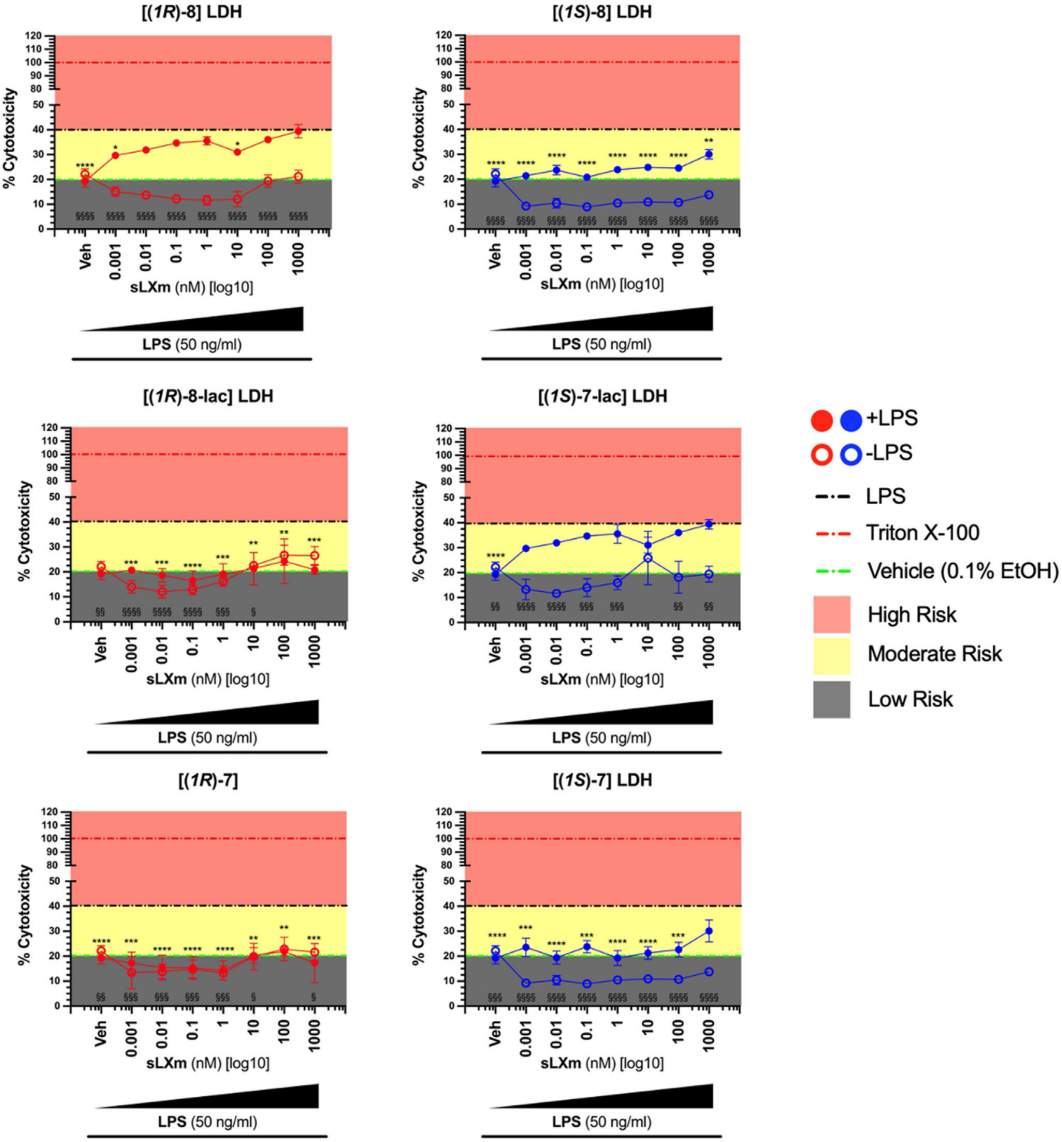
**Intrinsic/Extrinsic Cytotoxicity Profile of ISOQ Mimetics**. 1 x 10^5^ THP‐1 LUCIA monocytes were pre treated for 30 minutes. with increasing concentrations of the isoquinoline‐containing sLXms (10^−12^ M / 1 pM – 10^−6^ M / 1000 nM), vehicle, or appropriate controls (the latter shown in Supplementary Figure 3). This was followed by the addition of 50 ng/mL LPS. After 24 hours, LDH release was analyzed and expressed as a percentage of cytotoxicity. Concentration‐curve of the (R)‐epimers (red) and (S)‐epimers (blue), both with LPS stimulus (red/blue full points) and without LPS (red/blue hollow points). Triton X‐100 represents maximal LDH release from cells and has been set to 100%, allowing for benchmarks to be set at spontaneous LDH release (green line) and LPS release alone (black line). Data expressed as %Triton X‐100 ± SEM (n = 3). Data analysis performed as Two‐way ANOVA with Dunnett's multiple comparisons test comparing reduction of LDH compared to LPS LDH release, where p < 0.05 for LPS‐treated samples is annotated as *, and p < 0.05 is annotated by § for non‐LPS‐treated samples.

All Isoq sLXms show a protective effect in the absence of LPS stimulus, showing a reduced release of LDH and being primarily resident in the “low risk” zones relative to both Triton X‐100 and LPS. Furthermore, in the presence of LPS, all ISOQ sLXms remain within the safety margin by releasing less LDH than an LPS stimulus on its own (Figure 5). Although our lead compound (*1R*)‐**8** exhibits relatively “high” LDH release in comparison to its counterparts (39.4%, p > 0.99), its lowest LDH release is present coincidentally at its most optimal concentration in terms of NF‐κB activity reduction (10 nM, 31.0%, p < 0.05). These data further highlight the potential of (*1R*)‐**8** as a pro‐resolving agent and its safety.

## 4 Discussion

The traditional approach to treating inflammatory diseases has predominantly targeted the suppression of pro‐inflammatory mediators, with corticosteroids and NSAIDs serving as the cornerstone therapies. However, chronic usage of these agents is associated with substantial adverse effects, including immunosuppression, metabolic dysregulation, and tissue damage. This has prompted a paradigm shift in the field of immunopharmacology, from merely inhibiting inflammation to actively promoting its resolution. Resolution of inflammation is now recognized as a highly regulated, active process, rather than a passive termination of inflammatory signaling. SPMs, such as LXs, resolvins, protectins, and maresins, orchestrate this transition by promoting the clearance of apoptotic cells (efferocytosis), dampening further neutrophil recruitment, and re‐establishing tissue homeostasis. LXs, particularly LXA_4_, play a central role in this process by binding to the G‐protein‐coupled receptor ALX/FPR2, leading to anti‐inflammatory and pro‐resolving signaling cascades.

However, native LXA_4_ suffers from rapid inactivation in vivo, which limits its clinical utility. This has promoted the development of metabolically stable synthetic lipoxin analogues (sLXms), designed to mimic the function of the parent compound while overcoming its pharmacokinetic limitations. In this context, we have described herein the design, synthesis, and biological evaluation of novel 1,3‐ and 1,4‐disubstituted isoquinoline‐containing LXA_4_ analogues, aimed at enhancing receptor selectivity and promoting pro‐resolving functions with improved stability.

### 4.1 Synthetic Analogues as Pro‐resolving Therapeutics

The synthetic isoquinoline‐based LXA_4_ mimetics (Isoq‐sLXms) developed in this study were designed to improve on the limitations of native LXA_4_ by introducing heteroaromatic rings for greater metabolic stability and receptor affinity. Among the compounds synthesized, (*1R*)‐**8** emerged as a particularly promising candidate, demonstrating superior potency in suppressing NF‐κB activation and downstream pro‐inflammatory cytokines in monocyte‐derived in vitro models. Despite this impressive and significant suppression of pro‐inflammatory cytokine secretion, neither (*1R*)‐**8** nor any of the other compounds were as efficacious as the native LXA_4_ (**1**), despite a more superior reduction of NF‐κB activity from the luciferase assay (as previously reported, native LXA_4_ (**1**) had an IC_max_ of 10 pM for all cytokines (p < 0.001). For comparison, LXA_4_ achieved a reduction of 94 ± 1% for IL‐1β; 97 ± 1% for IL‐6; 74 ± 4% for IL‐12 p70; 70 ± 5% for IFN‐ɣ; 80 ± 5% for TNF.^[^ ^30^ ^]^

### 4.2 Receptor Activation and Functional Outcomes

Crucially, (*1R*)‐**8** was shown to act as a partial agonist of ALX/FPR2, eliciting calcium mobilization while not fully replicating the maximal receptor activation achieved by native LXA_4_. This partial agonism may confer therapeutic advantages by biasing the receptor toward resolution pathways without overstimulating the system, potentially reducing off‐target effects (in line with the principle of resolution pharmacology). While not achieving the same cytokine‐suppressing efficacy as LXA_4_ across the board, (*1R*)‐**8** and related compounds nonetheless induced robust suppression of key mediators such as IL‐6 and IL‐1β. Conversely, IL‐8 is a cytokine that has been shown to exhibit both pro‐ and antiinflammatory actions endogenously, and the increasing trend in production of this cytokine by these sLXms has been previously discussed.^[^ ^31^ ^]^ With this in mind, we can once again state that though these Isoq‐sLXms effectively modulate pro‐inflammatory responses to promote anti‐inflammatory/pro‐resolving actions, they are not as effective as previously described sLXms.

### 4.3 Resolution versus Immune‐Suppression: A Paradigmatic Mechanistic Shift

The data presented reinforce the importance of engaging resolution‐specific pathways as opposed to broadly inhibiting inflammation. This approach may preserve essential immune functions, while selectively turning off chronic inflammatory signaling (i.e., NFkB signaling). The activity profile of (*1R*)‐**8** in particular suggests that synthetic resolution‐targeted agents can be rationally designed to fine‐tune immune modulation. Furthermore, the lack of cytotoxicity at therapeutic concentrations indicates the safety of such compounds, an essential consideration for chronic inflammatory disease treatment.

## 5 Conclusion

This work underscores the therapeutic promise of synthetic LXA_4_ analogues in promoting the resolution of inflammation. Through rational design and structural optimization, the isoquinoline‐containing mimetic (*1R*)‐**8** was shown to effectively attenuate pro‐inflammatory signaling, activate the ALX/FPR2 receptor, and suppress key cytokines without significant cytotoxicity. While native LXA_4_ remains more potent in certain endpoints, its rapid degradation in vivo limits clinical applicability. Our findings contribute to a growing body of evidence supporting the resolution‐focused paradigm in inflammation therapy and validate isoquinoline‐derived scaffolds as a viable platform for next‐generation pro‐resolving agents.

Future studies should aim to evaluate these compounds in in vivo models of chronic inflammation and tissue repair to further establish their translational relevance. Optimization for pharmacokinetic parameters and receptor biasing will be the crucial next steps in bringing synthetic pro‐resolving mediators toward clinical application.

## 6 Experimental Section

### 6.1 General (Chemistry)

^1^H NMR (300, 400, or 500 MHz) and ^13^C NMR (75 or 126 MHz) were recorded at room temperature in CDCl_3_ with Varian‐Unity spectrometers. Chemical shifts (*δ*) are in parts per million relative to CHCl_3_ (7.26, ^1^H), CDCl_3_ (77.0, ^13^C), or to CH_3_CN (1.94, ^1^H), CD_3_CN (118.3, ^13^C). Coupling constants are given as absolute values expressed in hertz. High‐resolution mass spectra were measured on a Waters/Micromass instrument. Infrared spectra were recorded on a Perkin‐Elmer infrared FT spectrometer. Optical rotation values were measured on a Perkin‐Elmer polarimeter. Thin layer chromatography was carried out using Merck Kieselgel 60 F254 silica gel plates. Column chromatography separations were performed using Merck Kieselgel 60 (230–400 mesh). Solvents were dried immediately before use by distillation from standard drying agents. HPLC analyses were performed using a Waters Acquity UPC^2^ system or a Shimadzu LC‐2010A system.

#### 6.1.1 1‐(4‐Bromoisoquinolin‐1‐yl)hexan‐1‐one (9)

4‐Bromoisoquinoline (0.5 g, 2.40 mmol) was dissolved in EtOAc (20 mL) and hexanal (1.18 mL, 9.61 mmol) and TMSN_3_ (0.63 mL, 4.81 mmol) were added. (Bis(trifluoroacetoxy)iodo)benzene (2.07 g, 4.81 mmol) was added slowly over 10 minutes and the mixture was stirred at room temperature for 2 hours. Triethylamine (6.25 mL) was added and the reaction was stirred for 10 minutes. After removal of the solvents in vacuo the residue was purified by silica column chromatography (cyclohexane/MeOH, 99:1) to yield the ketone (0.5 g, 68 %) as an orange solid. TLC: *R_f_* = 0.52 (pentane/EtOAc, 19:1); ^1^H NMR (400 MHz, CDCl_3_) δ 8.85 (d, *J* = 8.6 Hz, 1H), 8.73 (s, 1H), 8.20 (d, *J* = 8.5 Hz, 1H), 7.79 (dd, *J* = 8.5, 7.6 Hz, 1H), 7.69 (dd, *J* = 8.6, 7.6 Hz, 1H), 3.26 (t, *J* = 7.4 Hz, 2H), 1.80 – 1.71 (m, 2H), 1.41 – 1.35 (m, 4H), 0.91 (t, *J* = 6.9 Hz, 3H); ^13^C NMR (101 MHz, CDCl_3_) δ 204.3, 152.6, 143.0, 135.7, 131.6, 129.8, 127.3, 127.0, 126.3, 123.6, 40.5, 31.6, 24.0, 22.7, 14.1; IR (neat) (ν_max_, cm^−1^) 3053, 2957, 1697, 1560, 1490, 1265; HRMS (ES) Found 306.0498 [M + H]^+^ C_15_H_17_NOBr requires 306.0494.

#### 6.1.2 (*5S*,*6R*,*E*)‐Methyl 5,6‐bis((tert‐butyldimethylsilyl)Oxy)‐8‐(1‐hexanoylisoquinolin‐4‐yl)oct‐7‐enoate (15)

Pd(OAc)_2_ (2.5 mg, 0.01 mmol), P(*o*‐tolyl)_3_ (8 mg, 0.03 mmol) and tributylamine (0.85 mL, 3.59 mmol) were sealed under N_2_ and stirred at 80 °C for 10 minutes. Ketone **9** (50 mg, 0.163 mmol) and alkene **10** (74 mg, 0.179 mmol) were added and the reaction mixture was stirred at 120 °C for 36 hours. After filtering through silica with EtOAc (250 mL) most of the solvent (200 mL) was removed in vacuo and the remaining solution was washed with 10 % (w/v) CuSO_4_ solution (3 x 20 mL). The organic layer was separated and washed with water (50 mL) and brine (50 mL) and dried over MgSO_4_. The solvent was removed in vacuo and the residue was purified by silica column chromatography (pentane/EtOAc, 19:1) to afford **15** (74 mg, 70 %) as an orange oil. TLC: *R_f_* = 0.31 (pentane/EtOAc, 19:1); [α]_D_^20^ ‐16.3 (c = 0.55, CHCl_3_); ^1^H NMR (400 MHz, CDCl_3_) δ 8.93 – 8.89 (d, *J* = 8.3 Hz, 1H), 8.65 (s, 1H), 8.11 (d, *J* = 8.3 Hz, 1H), 7.76 – 7.71 (m, 1H), 7.70 – 7.65 (m, 1H), 7.16 (d, *J* = 15.9 Hz, 1H), 6.40 (dd, *J* = 15.9, 6.7 Hz, 1H), 4.31 – 4.26 (m, 1H), 3.77 (m, 1H), 3.66 (s, 3H), 3.31 (t, *J* = 7.5 Hz, 2H), 2.34 (t, *J* = 7.2 Hz, 2H), 1.81 – 1.73 (m, 4H), 1.66 – 1.54 (m, 2H), 1.42 – 1.38 (m, 4H), 0.95 (s, 9H), 0.92 (t, *J* = 7.1 Hz, 3H), 0.87 (s, 9H), 0.09 (4 x s, 12H); ^13^C NMR (101 MHz, CDCl_3_) δ 205.0, 174.1, 152.5, 138.8, 137.4, 134.7, 131.9, 130.4, 128.8, 127.3, 125.5, 125.0, 123.3, 77.2, 76.2, 51.6, 40.4, 34.4, 33.4, 31.7, 26.1, 26.1, 24.1, 22.7, 20.7, 18.5, 18.3, 14.1, ‐3.8, ‐3.8, ‐4.4, ‐4.4; IR (neat) (ν_max_, cm^−1^) 3054, 2963, 2928, 2855, 1735, 1677, 1420, 1265; HRMS (ES) Found 642.3979 [M + H]^+^ C_36_H_60_NO_5_Si_2_ requires 642.4010.

#### 6.1.3 (*5S*,*6R*,*E*)‐Methyl 5,6‐bis((tert‐butyldimethylsilyl)Oxy)‐8‐(1‐((*R*)‐1‐hydroxyhexyl) Isoquinolin‐4‐yl)Oct‐7‐enoate ((*1R*)‐16)

A mixture of formic acid (0.03 mL, 670 µmol) and triethylamine (0.05 mL, 390 µmol) was added to ketone **15** (100 mg, 0.16 µmol) with RuCl‐(*p*‐cymene)[*R*, *R*‐TsDPEN] (5 mg, 8 µmol). The mixture was degassed by freeze‐thaw cycles and stirred at 27 °C for 24 hours. The mixture was neutralized with aqueous sat. NaHCO_3_ solution (5 mL) and diluted with EtOAc (10 mL). The organic phase was separated and the aqueous phase was extracted with EtOAc (3 x 20 mL). The combined organic phase was washed with water (20 mL) and brine (20 mL) and dried over MgSO_4_. The solvent was removed in vacuo and the residue was purified by silica column chromatography (pentane/EtOAc, 9:1) to afford (*1R*)‐**16** (55 mg, 55 %) as an orange oil. *de* = 96 %, as determined by chiral UPC^2^ using a Chiralpak IC column (CO_2_: *i*PrOH, 99:1 to 85:15 after 1 minute); flow rate: 3 mL/min; *t*_R_‐(*S*) = 1.91 minutes, *t*_R_‐(*R*) = 2.24 minutes; TLC: *R_f_* = 0.36 (pentane/EtOAc, 9:1); [α]_D_^20^ + 1.9 (c = 1.00, CHCl_3_); ^1^H NMR (400 MHz, CDCl_3_) δ 8.51 (s, 1H), 8.10 (d, *J* = 8.5 Hz, 1H), 8.04 (d, *J* = 8.4 Hz, 1H), 7.74 – 7.70 (m, 1H), 7.66 – 7.61 (m, 1H), 7.10 (d, *J* = 15.8 Hz, 1H), 6.30 (dd, *J* = 15.8, 6.8 Hz, 1H), 5.47 (t, *J* = 6.2 Hz, 1H), 5.08 (s, 1H), 4.27 (dd, *J* = 6.8, 4.9 Hz, 1H), 3.76 (m, 1H), 3.66 (s, 3H), 2.33 (t, *J* = 7.5 Hz, 2H), 2.01 – 1.92 (m, 1H), 1.83 – 1.56 (m, 6H), 1.53 – 1.44 (m, 1H), 1.34 – 1.26 (m, 4H), 0.94 (s, 9H), 0.89 – 0.84 (m, 12H), 0.16 – 0.04 (4 x s, 12H); ^13^C NMR (101 MHz, CDCl_3_) δ 174.1, 160.8, 138.1, 135.6, 134.5, 130.3, 128.3, 127.2, 125.1, 124.6, 124.4, 124.1, 77.3, 76.3, 69.8, 51.6, 39.6, 34.5, 33.3, 32.0, 26.2, 26.1, 25.5, 22.8, 20.8, 18.5, 18.4, 14.2, ‐3.8, ‐3.8, ‐4.4, ‐4.4; IR (neat) (ν_max_, cm^−1^) 3623, 3054, 2928, 2852, 1734, 1450, 1266, 1019, 740, 705; HRMS (ES) Found 644.4145 [M + H]^+^ C_36_H_62_NO_5_Si_2_ requires 644.4167.

#### 6.1.4 (*5S*,*6R*,*E*)‐Methyl 5,6‐bis((tert‐butyldimethylsilyl)Oxy)‐8‐(1‐((*S*)‐1‐hydroxyhexyl)Isoquinolin‐4‐yl)Oct‐7‐enoate ((*1S*)‐16)

A mixture of formic acid (0.03 mL, 670 µmol) and triethylamine (0.05 mL, 390 µmol) was added to a mixture of ketone **15** (100 mg, 0.16 µmol) and RuCl‐(*p*‐cymene)[*S*, *S*‐TsDPEN] (5 mg, 8 µmol) in anhydrous DMF (1 mL). The mixture was degassed by freeze‐thaw cycles and then stirred at 27 °C for 48 hours. The mixture was neutralized with aqueous sat. NaHCO_3_ solution (5 mL) and diluted with EtOAc (10 mL). The organic phase was separated and the aqueous phase extracted with EtOAc (3 x 20 mL). The combined organic phase was washed with water (20 mL) and brine (20 mL) and dried over MgSO_4_. The solvent was removed in vacuo and the residue was purified by silica column chromatography (pentane/EtOAc, 9:1) to afford (*1S*)‐**16** (73 mg, 73 %) as an orange oil. *de* = 88 %, as determined by chiral UPC^2^ using a Chiralpak IC column (CO_2_: *i*PrOH, 99:1 to 85:15 after 1 minute); flow rate: 3 mL/min; *t*_R_‐(*S*) = 2.03 min, *t*_R_‐(*R*) = 2.37 minutes; TLC: *R_f_* = 0.42 (pentane/EtOAc, 9:1); [α]_D_^20^ ‐21.9 (c = 1.00, CHCl_3_); ^1^H NMR (400 MHz, CDCl_3_) δ 8.51 (s, 1H), 8.10 (d, *J* = 8.4 Hz, 1H), 8.04 (d, *J* = 8.4 Hz, 1H), 7.76 – 7.71 (m, 1H), 7.66 – 7.60 (m, 1H), 7.10 (d, *J* = 15.8 Hz, 1H), 6.28 (dd, *J* = 15.8, 6.8 Hz, 1H), 5.48 – 5.42 (m, 1H), 4.27 (dd, *J* = 6.8, 4.5 Hz, 1H), 3.79 – 3.73 (m, 1H), 3.66 (s, 3H), 2.33 (t, *J* = 7.4 Hz, 2H), 2.01 – 1.92 (m, 1H), 1.83 – 1.70 (m, 2H), 1.66 – 1.46 (m, 5H), 1.33 – 1.22 (m, 4H), 0.96 – 0.93 (m, 9H), 0.87 (s, 12H) 0.17 – 0.02 (4 x s, 12H); ^13^C NMR (101 MHz, CDCl_3_) δ 174.1, 160.8, 138.1, 135.6, 134.5, 130.3, 128.3, 127.2, 125.2, 124.6, 124.4, 124.1, 77.3, 76.2, 69.8, 51.6, 39.6, 34.5, 33.3, 32.0, 26.1, 26.1, 25.6, 22.8, 20.8, 18.5, 18.3, 14.2, ‐3.8, ‐3.8, ‐4.4, ‐4.4; IR (neat) (ν_max_, cm^−1^) 3428, 3055, 2929, 2852, 1735, 1642, 1450, 1265, 1019, 740; HRMS (ES) Found 644.4195 [M + H]^+^ C_36_H_62_NO_5_Si_2_ requires 644.4167.

#### 6.1.5 (*5S*,*6R*,*E*)‐Methyl 5,6‐dihydroxy‐8‐(1‐((*R*)‐1‐hydroxyhexyl)Isoquinolin‐4‐yl)Oct‐7‐enoate ((*1R*)‐7)

Tetra‐*n*‐butylammonium fluoride (0.20 mL, 1 M in THF, 0.20 mmol) was added to alcohol (*1R*)‐**16** (50 mg, 0.08 mmol) in anhydrous THF (3 mL) and the solution was stirred at room temperature for 16 hours. The solvent was removed in vacuo at 25 °C and the residue was purified by preparative TLC (CH_2_Cl_2_/MeOH, 96:4) to afford (*1R*)‐**7** (14 mg, 40 %) as a yellow oil. TLC: *R_f_* = 0.13 (CH_2_Cl_2_/MeOH, 96:4); [α]_D_^20^ + 22.9 (c = 0.32, CHCl_3_); ^1^H NMR (500 MHz, CD_3_CN) δ 8.58 (s, 1H), 8.24 (m, 2H), 7.85 (t, *J* = 7.5 Hz, 1H), 7.73 (t, *J* = 7.8 Hz, 1H), 7.29 (d, *J* = 15.6 Hz, 1H), 6.44 (dd, *J* = 15.6, 6.2 Hz, 1H), 5.47 – 5.42 (m, 1H), 4.27 (dd, *J* = 5.1, 4.8 Hz, 1H), 3.69 – 3.64 (m, 1H), 3.63 (s, 3H), 3.34 (s, 1H), 2.99 (s, 1H), 2.37 (t, *J* = 6.9 Hz, 2H), 1.87 – 1.26 (m, 12H) 0.89 (t, *J* = 6.9 Hz, 3H); ^13^C NMR (126 MHz, CD_3_CN) δ 174.8, 162.0, 138.8, 135.5, 135.1, 131.5, 129.3, 128.2, 125.9, 125.8, 125.2, 124.8, 76.3, 74.8, 70.6, 51.8, 39.7, 34.4, 32.6, 32.4, 26.1, 23.3, 22.2, 14.3; IR (neat) (ν_max_, cm^−1^) 3392, 2959, 2930, 1733, 1649, 1569, 1487, 1462, 1380, 1252, 1068; HRMS (ES) Found 416.2444 [M + H]^+^ C_24_H_34_NO_5_ requires 416.2437.

#### 6.1.6 (*5S*,*6R*,*E*)‐Methyl 5,6‐dihydroxy‐8‐(1‐((*S*)‐1‐hydroxyhexyl)Isoquinolin‐4‐yl)Oct‐7‐enoate ((*1S*)‐7)

Tetra‐*n*‐butylammonium fluoride (0.20 mL, 1 M in THF, 0.20 mmol) was added to a solution of alcohol (*1S*)‐**16** (50 mg, 0.08 mmol) in anhydrous THF (3 mL) and the solution was stirred at room temperature for 16 hours. The solvent was removed in vacuo at 25 °C and the residue was purified by preparative TLC (CH_2_Cl_2_/MeOH, 96:4) to afford (*1S*)‐**7** (13 mg, 38 %) as a yellow wax. TLC: *R_f_* = 0.13 (CH_2_Cl_2_/MeOH, 96:4); [α]_D_^20^ ‐29.8 (c = 3.00, CHCl_3_); ^1^H NMR (500 MHz, CDCl_3_) δ 8.52 (s, 1H), 8.10 (d, *J* = 8.5 Hz, 1H), 8.04 (d, *J* = 8.4 Hz, 1H), 7.74 (t, *J* = 7.3 Hz, 1H), 7.63 (t, *J* = 7.5 Hz, 1H), 7.27 (d, *J* = 15.7 Hz, 1H), 6.36 (dd, *J* = 15.7, 6.0 Hz, 1H), 5.45 (dd, *J* = 7.8, 2.8 Hz, 1H), 4.42 (dd, *J* = 6.0, 3.9 Hz, 1H), 3.84 (dt, *J* = 8.9, 3.9 Hz, 1H), 3.66 (s, 3H), 3.02 – 2.96 (s, 1H), 2.39 (td, *J* = 7.2, 3.4 Hz, 2H), 1.98 – 1.87 (m, 2H), 1.83 – 1.23 (m, 12H), 0.87 (t, *J* = 7.0 Hz, 3H); ^13^C NMR (126 MHz, CDCl_3_) δ 174.3, 161.2, 138.1, 134.4, 132.4, 130.5, 127.9, 127.4, 126.7, 124.6, 124.3, 124.1, 75.8, 74.0, 69.8, 51.8, 39.5, 33.8, 31.9, 31.7, 25.5, 22.8, 21.2, 14.2; IR (neat) (ν_max_, cm^−1^) 3420, 3054, 2987, 2927, 1729, 1648, 1421, 1265; HRMS (ES) Found 416.2422 [M + H]^+^ C_24_H_34_NO_5_ requires 416.2437.

#### 6.1.7 (*S*)‐6‐((*R*,*E*)‐1‐hydroxy‐3‐(1‐((*S*)‐1‐hydroxyhexyl)Isoquinolin‐4‐yl)Allyl)Tetrahydro‐2H‐pyran‐2‐one ((*1S*)‐7‐lac)

The lactone was isolated by preparative TLC (CH_2_Cl_2_/MeOH, 96:4) to yield (*1S*)‐**7‐lac** (8 mg, 21 %) as a yellow oil. TLC: *R_f_* = 0.14 (CH_2_Cl_2_/MeOH, 96:4); [α]_D_^20^ ‐26.2 (c = 0.85, CHCl_3_); ^1^H NMR (500 MHz, CDCl_3_) δ 8.52 (s, 1H), 8.16 – 8.04 (m, 2H), 7.83 – 7.73 (m, 1H), 7.71 – 7.64 (m, 1H), 7.40 (d, *J* = 15.8 Hz, 1H), 6.30 (dd, *J* = 15.8, 5.4 Hz, 1H), 5.53 – 5.43 (m, 1H), 4.78 – 4.67 (m, 1H), 4.59 – 4.46 (m, 1H), 2.78 – 2.43 (m, 2H), 2.11 – 1.85 (m, 5H), 1.74 – 1.24 (m, 9H), 0.89 (t, *J* = 5.9 Hz, 3H); ^13^C NMR (126 MHz, CDCl_3_) δ 171.4, 161.4, 138.0, 134.4, 130.7, 130.7, 127.8, 127.5, 127.0, 124.7, 124.3, 124.1, 82.9, 73.3, 69.8, 39.5, 31.9, 29.9, 25.5, 22.8, 21.9, 18.5, 14.2; IR (neat) (ν_max_, cm^−1^) 3675, 3408, 2966, 1730, 1651, 1568, 1381, 1251, 1066; HRMS (ES) Found 384.2185 [M + H]^+^ C_23_H_30_NO_4_ requires 384.2175.

#### 6.1.8 1‐(3‐Bromoisoquinolin‐1‐yl)hexan‐1‐one (18)

3‐Bromoisoquinoline **17** (0.5 g, 2.40 mmol) was dissolved in EtOAc (20 mL). Hexanal (1.18 mL, 9.61 mmol) and TMSN_3_ (0.63 mL, 4.81 mmol) were added. (Bis(trifluoroacetoxy)iodo)benzene (2.07 g, 4.81 mmol) was added slowly over 45 minutes and the mixture was stirred at room temperature for 2 hours. Triethylamine (6.25 mL) was added and the reaction was stirred for 10 minutes. After removal of the solvents in vacuo the residue was purified by silica column chromatography (pentane/EtOAc, 19:1) to yield the ketone **18** (0.44 g, 60 %) as an orange oil. TLC: *R_f_* = 0.65 (pentane/EtOAc, 19:1); ^1^H NMR (400 MHz, CDCl_3_) δ 8.81 (d, *J* = 8.3 Hz, 1H), 8.03 (s, 1H), 7.83 – 7.63 (m, 3H), 3.29 (t, *J* = 7.4 Hz, 2H), 1.81 – 1.73 (m, 2H), 1.42 – 1.37 (m, 4H), 0.92 (t, *J* = 7.1 Hz, 3H); ^13^C NMR (101 MHz, CDCl_3_) δ 203.7, 153.8, 139.4, 133.8, 131.4, 129.5, 127.8, 127.2, 126.2, 124.8, 40.4, 31.6, 23.9, 22.7, 14.1; IR (neat) (ν_max_, cm^−1^) 3056, 2986, 2957, 1700, 1560, 1490, 1266, 1046; HRMS (ES) Found 306.0489 [M + H]^+^ C_15_H_17_NOBr requires 306.0494.

#### 6.1.9 (*5S*,*6R*,*E*)‐Methyl 5,6‐bis((tert‐butyldimethylsilyl)Oxy)‐8‐(1‐hexanoylisoquinolin‐3‐yl)oct‐7‐enoate (19)

Pd(OAc)_2_ (9 mg, 0.04 mmol), P(*o*‐tolyl)_3_ (27 mg, 0.09 mmol) and tributylamine (3.55 mL, 15.0 mmol) were sealed under N_2_ and stirred at 80 °C for 10 minutes. Ketone **18** (208 mg, 0.68 mmol) and alkene **10** (341 mg, 0.82 mmol) were added and the reaction mixture was stirred at 120 °C for 72 hours. After filtering through silica with EtOAc (250 mL) most of the solvent (200 mL) was removed in vacuo and the remaining solution was washed with 10 % (w/v) CuSO_4_ solution (3 x 20 mL). The organic layer was separated and washed with water (50 mL) and brine (50 mL) and dried over MgSO_4_. The solvent was removed in vacuo and the residue was purified by silica column chromatography (pentane/EtOAc, 19:1) to afford **19** (255 mg, 40 %) as an orange oil. TLC: *R_f_* = 0.38 (pentane/EtOAc, 19:1); [α]_D_^20^ ‐10.0 (c = 1.00, CHCl_3_); ^1^H NMR (400 MHz, CDCl_3_) δ 8.80 (d, *J* = 8.8 Hz, 1H), 7.80 (d, *J* = 8.1 Hz, 1H), 7.68 – 7.63 (m, 1H), 7.61 – 7.55 (m, 2H), 6.94 (dd, *J* = 15.5, 7.1 Hz, 1H), 6.70 (d, *J* = 15.5 Hz, 1H), 4.24 (dd, *J* = 7.1, 5.4 Hz, 1H), 3.72 (dt, *J* = 6.4, 5.4 Hz, 1H), 3.66 (s, 3H), 3.34 (t, *J* = 7.5, 2H), 2.33 (t, *J* = 7.3 Hz, 2H), 1.81 – 1.74 (m, 4H), 1.68 – 1.58 (m, 2H) 1.43 – 1.37 (m, 4H), 0.96 – 0.90 (m, 15H) 0.86 (s, 9H), 0.13 – 0.00 (4 x s, 12H); ^13^C NMR (101 MHz, CDCl_3_) δ 205.4, 174.2, 153.2, 147.4, 138.0, 135.9, 130.4, 130.2, 128.4, 127.2, 127.0, 125.0, 121.5, 76.9, 76.0, 51.6, 40.4, 34.6, 33.2, 31.8, 26.1, 26.1, 24.2, 22.7, 20.5, 18.4, 18.3, 14.2, ‐3.7, ‐3.9, ‐4.3, ‐4.5; IR (neat) (ν_max_, cm^−1^) 3054, 2929, 2852, 1734, 1680, 1422, 1265; HRMS (ES) Found 664.3820 [M + Na]^+^ C_36_H_59_NO_5_NaSi_2_ requires 664.3830.

#### 6.1.10 (*5S*,*6R*,*E*)‐Methyl 5,6‐bis((tert‐butyldimethylsilyl)Oxy)‐8‐(1‐((*R*)‐1‐hydroxyhexyl)Isoquinolin‐3‐yl)Oct‐7‐enoate ((*1R*)‐20)

A mixture of formic acid (0.06 mL, 1.61 mmol) and triethylamine (0.13 mL, 0.94 mmol) was added to ketone **19** (240 mg, 0.37 mmol) and RuCl‐(*p*‐cymene)[*R*,*R*‐TsDPEN] (19 mg, 0.02 mmol). Anhydrous DMF (1.8 mL) was added and the mixture was degassed by freeze‐thaw cycles and then stirred at 27 °C for 24 hours. The mixture was neutralized with aqueous sat. NaHCO_3_ (5 mL) and diluted with EtOAc (10 mL). The organic phase was separated and the aqueous phase was extracted with EtOAc (3 x 20 mL). The combined organic phase was washed with water (20 mL) and brine (20 mL) and dried over MgSO_4_. The solvent was removed in vacuo and the residue was purified by silica column chromatography (pentane/EtOAc, 9:1) to afford (*1R*)‐**20** (119 mg, 50 %) as an orange oil. *de* = 94 %, as determined by chiral UHPLC using a Chiralpak OD column (Heptane:*i*PrOH, 98.5:1,5); flow rate: 1 mL/min; *t*_R_‐(*S*) = 3.93 minutes, *t*_R_‐(*R*) = 5.69 minutes; TLC: *R_f_* = 0.44 (pentane/EtOAc, 9:1); [α]_D_^20^ + 6.0 (c = 1.00, CHCl_3_); ^1^H NMR (400 MHz, CDCl_3_) δ 7.97 (d, *J* = 8.4 Hz, 1H), 7.81 (d, *J* = 8.1 Hz, 1H), 7.70 – 7.62 (m, 1H), 7.58 – 7.51 (m, 1H), 7.39 (s, 1H), 6.86 (dd, *J* = 15.5, 7.0 Hz, 1H), 6.67 (d, *J* = 15.5 Hz, 1H), 5.46 – 5.39 (m, 1H), 5.36 – 5.30 (m, 1H), 4.22 (dd, *J* = 7.6, 6.1 Hz, 1H), 3.73 – 3.67 (m, 1H), 3.66 (s, 3H), 2.32 (t, *J* = 7.3 Hz, 2H), 2.01 – 1.54 (m, 6H), 1.37 – 1.27 (m, 6H), 0.94 – 0.82 (m, 21H), 0.15 – ‐0.04 (4 x s, 12H); ^13^C NMR (101 MHz, CDCl_3_) δ 174.2, 161.4, 146.5, 137.5, 135.4, 130.5, 130.2, 127.8, 126.9, 124.4, 124.2, 118.1, 77.4, 75.9, 69.7, 51.6, 39.4, 34.6, 33.0, 32.0, 29.9, 26.1, 26.1, 22.8, 20.5, 18.4, 18.3, 14.2, ‐3.7, ‐3.9, ‐4.3, ‐4.5; IR (neat) (ν_max_, cm^−1^) 3445, 3054, 2986, 2929, 2852, 1734, 1450, 1422, 1265; HRMS (ES) Found 644.4144 [M + H]^+^ C_36_H_62_NO_5_Si_2_ requires 644.4167.

#### 6.1.11 (*5S*,*6R*,*E*)‐Methyl 5,6‐bis((tert‐butyldimethylsilyl)Oxy)‐8‐(1‐(*S*)‐1‐hydroxyhexyl)Isoquinolin‐3‐yl)Oct‐7‐enoate ((*1S*)‐20)

A mixture of formic acid (0.03 mL, 0.67 mmol) and triethylamine (0.06 mL, 0.39 mmol) was added to ketone **19** (100 mg, 0.16 mmol) and RuCl‐(*p*‐cymene)[*R*,*R*‐TsDPEN] (5 mg, 0.01 mmol). Anhydrous DMF (0.7 mL) was added and the mixture was degassed by freeze‐thaw cycles and then stirred at 27 °C for 39 hours. The mixture was neutralized with aqueous sat. NaHCO_3_ (5 mL) and diluted with EtOAc (10 mL). The organic phase was separated and the aqueous phase was extracted with EtOAc (3 x 20 mL). The combined organic phase was washed with water (20 mL) and brine (20 mL) and dried over MgSO_4_. The solvent was removed in vacuo and the residue was purified by silica column chromatography (pentane/EtOAc, 9:1) to afford (*1S*)‐**20** (65 mg, 65 %) as an orange oil. *de* = 94 %, as determined by chiral UHPLC using a Chiralpak OD column (Heptane:*i*PrOH, 98.5:1.5); flow rate: 1 mL/min; *t*_R_‐(*S*) = 3.95 minutes, *t*_R_‐(*R*) = 5.65 minutes; TLC: *R_f_* = 0.41 (pentane/EtOAc, 9:1); [α]_D_^20^ ‐14.0 (c = 1.00, CHCl_3_); ^1^H NMR (400 MHz, CDCl_3_) δ 7.97 (d, *J* = 8.4 Hz, 1H), 7.81 (d, *J* = 8.2 Hz, 1H), 7.69 – 7.62 (m, 1H), 7.57 – 7.51 (m, 1H), 7.40 (s, 1H), 6.89 (dd, *J* = 15.5, 6.7 Hz, 1H), 6.68 (d, *J* = 15.5 Hz, 1H), 5.47 – 5.37 (m, 1H), 4.23 (t, *J* = 5.7 Hz, 1H), 3.73 – 3.67 (m, 1H), 3.65 (s, 3H), 3.38 – 3.24 (m, 1H), 2.32 (t, *J* = 7.3 Hz, 2H), 1.82 – 1.53 (m, 6H), 1.51 – 1.18 (m, 6H), 0.97 – 0.81 (m, 21H), 0.13 – ‐0.02 (m, 12H); ^13^C NMR (101 MHz, CDCl_3_) δ 174.2, 161.3, 146.5, 137.4, 135.3, 130.5, 130.0, 127.8, 126.9, 124.4, 124.2, 118.1, 76.7, 76.0, 69.7, 51.6, 39.4, 34.6, 33.0, 32.0, 29.8, 26.1, 26.1, 22.8, 20.5, 18.4, 18.3, 14.2, ‐3.8, ‐3.9, ‐4.3, ‐4.5; IR (neat) (ν_max_, cm^−1^) 3407, 3055, 2968, 2928, 2856, 1734, 1643, 1450, 1422, 1265; HRMS (ES) Found 644.4156 [M + H]^+^ C_36_H_62_NO_5_Si_2_ requires 644.4167.

#### 6.1.12 (*5S*,*6R*,*E*)‐Methyl 5,6‐dihydroxy‐8‐(1‐(*R*)‐1‐hydroxyhexyl)Isoquinolin‐3‐yl)Oct‐7‐enoate (*1R*)‐8

Tetra‐*n*‐butylammonium fluoride (0.23 mL, 1 M in THF, 0.23 mmol) was added to alcohol (*1R*)‐**20** (60 mg, 0.09 mmol) in anhydrous THF (3.6 mL) and the solution was stirred at room temperature for 16 hours. The solvent was removed in vacuo at 25 °C and the residue was purified by preparative TLC (CH_2_Cl_2_/MeOH, 96:4) to afford (*1R*)‐**8** (11.6 mg, 30 %) as a yellow oil. TLC: *R_f_* = 0.16 (CH_2_Cl_2_/MeOH, 96:4); [α]_D_^20^ + 17.5 (c = 0.11, CHCl_3_); ^1^H NMR (400 MHz, CD_3_CN) δ 8.13 (d, *J* = 8.4 Hz, 1H), 7.89 (d, *J* = 8.2 Hz, 1H), 7.76 – 7.69 (m, 1H), 7.63 – 7.56 (m, 2H), 7.00 (dd, *J* = 15.6, 6.0 Hz, 1H), 6.82 (d, *J* = 15.6 Hz, 1H), 5.43 – 5.35 (m, 1H), 4.26 – 4.13 (m, 1H), 3.65 – 3.60 (m, 1H), 3.59 (s, 3H), 2.32 (t, *J* = 7.2 Hz, 2H), 1.85 – 1.24 (m, 15H), 0.86 (t, *J* = 7.0 Hz, 3H); ^13^C NMR (101 MHz, CD_3_CN) δ 174.8, 162.7, 147.4, 138.3, 134.2, 131.6, 130.8, 128.6, 127.9, 125.6, 125.0, 119.0, 76.0, 74.8, 70.6, 51.8, 39.8, 34.4, 32.5, 32.4, 26.2, 23.3, 22.2, 14.3; IR (neat) (ν_max_, cm^−1^) 3402, 3055, 2956, 2854, 1724, 1645, 1462, 1378, 1259, 1163; HRMS (ES) Found 416.2420 [M + H]^+^ C_24_H_34_NO_5_ requires 416.2437.

#### 6.1.13 (*S*)‐6‐((*R*,*E*)‐1‐hydroxy‐3‐(1‐(*R*)‐1‐hydroxyhexyl)Isoquinolin‐3‐yl)Allyl)Tetrahydro‐2H‐pyran‐2‐one ((*1R*)‐8‐lac)

The lactone was isolated by preparative TLC (CH_2_Cl_2_/MeOH, 96:4) to yield (*1R*)‐**8‐lac** (7 mg, 18 %) as a yellow oil. TLC: *R_f_* = 0.26 (CH_2_Cl_2_/MeOH, 96:4); [α]_D_^20^ + 5.5 (c = 0.24, CHCl_3_); ^1^H NMR (500 MHz, CDCl_3_) δ 7.98 (d, *J* = 8.3 Hz, 1H), 7.82 (d, *J* = 8.4 Hz, 1H), 7.68 (t, *J* = 7.7 Hz, 1H), 7.58 (t, *J* = 7.5 Hz, 1H), 7.46 (s, 1H), 7.00 – 6.85 (m, 2H), 5.45 – 5.41 (m, 1H), 4.75 – 4.70 (m, 1H), 4.53 – 4.46 (m, 1H), 2.69 – 2.60 (m, 1H), 2.49 (ddd, *J* = 16.8, 12.4, 4.7 Hz, 1H), 2.01 – 1.92 (m, 2H), 1.90 – 1.81 (m, 1H), 1.67 – 1.57 (m, 3H), 1.40 – 1.27 (m, 8H), 0.88 (t, *J* = 6.5 Hz, 3H); ^13^C NMR (126 MHz, CDCl_3_) δ 171.7, 161.6, 145.6, 137.3, 132.3, 131.3, 130.7, 128.7, 127.9, 127.3, 124.4, 119.0, 83.0, 73.0, 69.9, 39.5, 31.9, 29.9, 25.7, 22.8, 21.5, 18.5, 14.2; IR (neat) (ν_max_, cm^−1^) 3433, 3056, 2924, 1730, 1642, 1427, 1265, 1115; HRMS (ES) Found 384.2179 [M + H]^+^ C_23_H_30_NO_4_ requires 384.2175.

#### 6.1.14 (*5S*,*6R*,*E*)‐Methyl 5,6‐dihydroxy‐8‐(1‐(*S*)‐1‐hydroxyhexyl)Isoquinolin‐3‐yl)Oct‐7‐enoate ((*1S*)‐8)

Tetra‐*n*‐butylammonium fluoride (0.20 mL, 1 M in THF, 0.20 mmol) was added to alcohol (*1S*)‐**20** (50 mg, 0.08 mmol) in anhydrous THF (3 mL) and the solution was stirred at room temperature for 16 hours. The solvent was removed in vacuo at 25 °C and the residue was purified by preparative TLC (CH_2_Cl_2_/MeOH, 96:4) to afford (*1S*)‐**8** (11 mg, 34 %) as a yellow oil. TLC: *R_f_* = 0.21 (CH_2_Cl_2_/MeOH, 96:4); [α]_D_^20^ ‐2.6 (c = 0.45, CHCl_3_); ^1^H NMR (400 MHz, CD_3_CN) δ 8.13 (d, *J* = 8.4 Hz, 1H), 7.89 (d, *J* = 8.2 Hz, 1H), 7.75 – 7.69 (m, 1H), 7.63 – 7.57 (m, 2H), 6.99 (dd, *J* = 15.5, 6.2 Hz, 1H), 6.81 (d, *J* = 15.5 Hz, 1H), 5.41 – 5.37 (m, 1H), 4.20 (m, 1H), 3.65 – 3.60 (m, 1H), 3.59 (s, 3H), 3.29 – 3.16 (m, 1H), 3.03 – 2.85 (m, 1H), 2.32 (t, *J* = 7.2 Hz, 2H), 1.84 – 1.74 (m, 1H), 1.69– 1.28 (m, 12H), 0.86 (t, *J* = 7.0 Hz, 3H); ^13^C NMR (101 MHz, CD_3_CN) δ 174.8, 162.7, 147.4, 138.3, 134.2, 131.6, 130.9, 128.6, 128.0, 125.6, 125.0, 119.0, 76.1, 74.9, 70.6, 51.8, 39.8, 34.4, 32.5, 32.5, 26.2, 23.3, 22.2, 14.3; IR (neat) (ν_max_, cm^−1^) 3424, 3055, 2988, 1723, 1642, 1422, 1368, 1265; HRMS (ES) Found 438.2238 [M + Na]^+^ C_24_H_33_NO_5_Na requires 433.2256.

### 6.2 Biological Evaluation Material and Methods

#### 6.2.1 LPS‐driven NF‐κB Activity in THP‐1 Monocytes

As previously described,^[^ ^30^, ^31^ ^]^ LPS‐driven NF‐κB activity was analyzed using THP‐1 LUCIA cells by measuring luminescence signals from the cell supernatant of sLXms‐treated cells, against untreated‐ or vehicle‐treated cells. Specifically, cells were pre‐treated for 30 minutes. with native LXA_4_ or Isoq‐sLXms (concentration range: 1 pM ‐ 1 µM), before an LPS stimulus (50 ng/mL) was applied for 24 hours. Previously analyzed native LXA_4_ (**1**), Benzo‐LXA_4_ (**3a**), AT‐01‐KG, and AT‐02‐CT (were used as reference compounds, with 1 x 10^8^ heat‐killed *listeria mono‐cytogenes* (HKLM) serving as an intrinsic positive control (Figure 4c).^[^ ^30^, ^31^ ^]^

#### 6.2.2 LPS‐driven Pro‐inflammatory Cytokine Release in THP‐1 Monocytes

The Isoq‐sLXms ability to regulate cytokine release was assessed via electro‐chemi‐luminescence‐based multiplex enzyme‐linked immunosorbent assay (ELISA) analyzing the secretion of several pivotal inflammatory cytokines downstream of NF‐κB activation [specifically, IFN‐ɣ, IL‐10, IL‐12 p70, IL‐1β, IL‐6, IL‐8, and TNF] were measured from culture media obtained from THP‐1 monocytes treated with LPS with or without sLXms (Supplementary Figure 4). The measurement was performed as per manufacturer instruction (Mesoscale Delivery).

#### 6.2.3 Intracellular Calcium Flux in Stably Double Transfected FPR2^+^/Gaq^+^‐HEK‐293 Cells

ALX/FPR2 is a G‐coupled receptor which is activated by endogenous ligands such as LXA_4_. Here, the affinity for the SPM receptor was assessed using stably double transfected HEK‐293 overexpressing ALX/FPR2 receptor coupled to a G⍶_q_ subunit. The activation of this receptor triggers intracellular Ca^2^flux can be fluorescently measured, thus activation of the receptor via (*1R*)‐**8** was determined by measuring Ca^2+^ flux.

Wild‐type HEK‐293 cells served as the control for this experiment, to verify specificity for the receptor, as previously described.^[^ ^30^ ^]^ Briefly, cells treated with 10–100 nM LXA_4_ or (*1R*)‐**8** were subjected to analysis, using ATP (P2Y agonist) (1 mM) and W peptide (FPR2 agonist) (2 nM and 20 pM) as internal controls. W peptide is a synthetic, potent, agonist of ALX/FPR2, whereas ATP functions through GPCR purinergic receptor P2Y expressed in HEK‐293 cells, inducing Ca^2+^ mobilization, independently of ALX/FPR2.

## 7 Statistical analysis

Graphs and statistical analyses were performed using GraphPad Prism (version 10.4.1). Data represent the mean ± SEM from at least three independent experiments and are expressed as a percentage of the positive control. To compare the effects of vehicle‐treated versus sLXms‐treated cells, a one‐way ANOVA was conducted, followed by Dunnett's multiple comparisons test as a post hoc analysis to assess differences between individual groups. A critical alpha value (α) of 0.05 was used, and differences were considered statistically significant at *p* < 0.05.

## 8 Note

Lactones (*1R*)‐**7**‐**lac** and (*1S*)‐**8**‐**lac** proved difficult to fully purify and isolate from their corresponding methyl esters and hence were not subjected to biological testing.

## Supporting Information

^1^H and ^13^C NMR spectra for novel compounds and HPLC chromatograms for key chiral intermediates and products are provided. Supplementary tables relevant to the biological evaluation are supplied. This material is available free of charge via the Internet at xxxx.

## Author Contributions

The manuscript was written through contributions of all authors. All authors have given approval to the final version of the manuscript. D.M. and P.G. wrote the chemical aspect of the manuscript. M.d.G. wrote the biological component of the introduction and discussion of the manuscript and set up and performed all the in vitro experiments and set up the data analysis. B.M. wrote the results section of the biological component of the manuscript, and also performed the data analysis and generated the biological graphical elements. P.G. and C.G. reviewed the manuscript and conceived the study.

## Conflict of Interest

The authors declare no conflict of interest.

## Supporting information



Supporting Information

## Data Availability

The data that support the findings of this study are available from the corresponding author upon reasonable request.
