# Supplementary material for: Asymmetric Synthesis and Biological Evaluation of 1,3‐ and 1,4‐Disubstituted Isoquinoline‐Containing Lipoxin A4 Analogues
Source: Chemistry. 2025 Aug 27;31(56):e02091. doi: 10.1002/chem.202502091 (PMC12510138; doi:10.1002/chem.202502091)
Supplement: Supplementary file 1 — Supporting Information [file CHEM-31-e02091-s001.pdf]

Asymmetric Synthesis and Biological Evaluation of 1,3- and 1,4-Disubstituted Isoquinoline-containing Lipoxin A_4 Analogues.

Denise Moran[a‡], Monica de Gaetano[b‡], Braden Millar[b], Catherine Godson[c] and Patrick J. Guiry*[,a]

[a] Centre for Synthesis & Chemical Biology, School of Chemistry, University College Dublin, Belfield, Dublin 4, Ireland

[b] UCD Conway Institute & School of Biomolecular & Biomedical Science; University College Dublin, Belfield, Dublin 4, Ireland

[c] UCD Conway Institute & School of Medicine; University College Dublin, Belfield, Dublin 4, Ireland

Supporting Information

Table of Contents

General Experimental Information	3
^{1}H and ^{13}C NMR Spectra	4-18
HPLC Chromatograms	16-31
Supplementary Figures 1-3	32-34

General Experimental Information (Chemistry)

^{1}H NMR (300, 400 or 500 MHz) and ^{13}C NMR (75 or 126 MHz) were recorded at room temperature in $CDCl_3$ with Varian-Unity spectrometers. Chemical shifts (δ) are in parts per million relative to $CHCl_3$ (7.26, ^{1}H), $CDCl_3$ (77.0, ^{13}C) or to CH_3CN (1.94, ^{1}H), CD_3CN (118.3, ^{13}C). Coupling constants are given as absolute values expressed in Hertz. High-resolution mass spectra were measured on a Waters/Micromass instrument. Infrared spectra were recorded on a Perkin-Elmer infrared FT spectrometer. Optical rotation values were measured on a Perkin-Elmer polarimeter. Thin layer chromatography was carried out using Merck Kieselgel 60 F254 silica gel plates. Column chromatography separations were performed using Merck Kieselgel 60 (230-400 mesh). Solvents were dried immediately before use by distillation from standard drying agents. HPLC analyses were performed using a Waters Acquity UPC^2 system or a Shimadzu LC-2010A system.

^{1}H and ^{13}C NMR Spectra

Compound 9

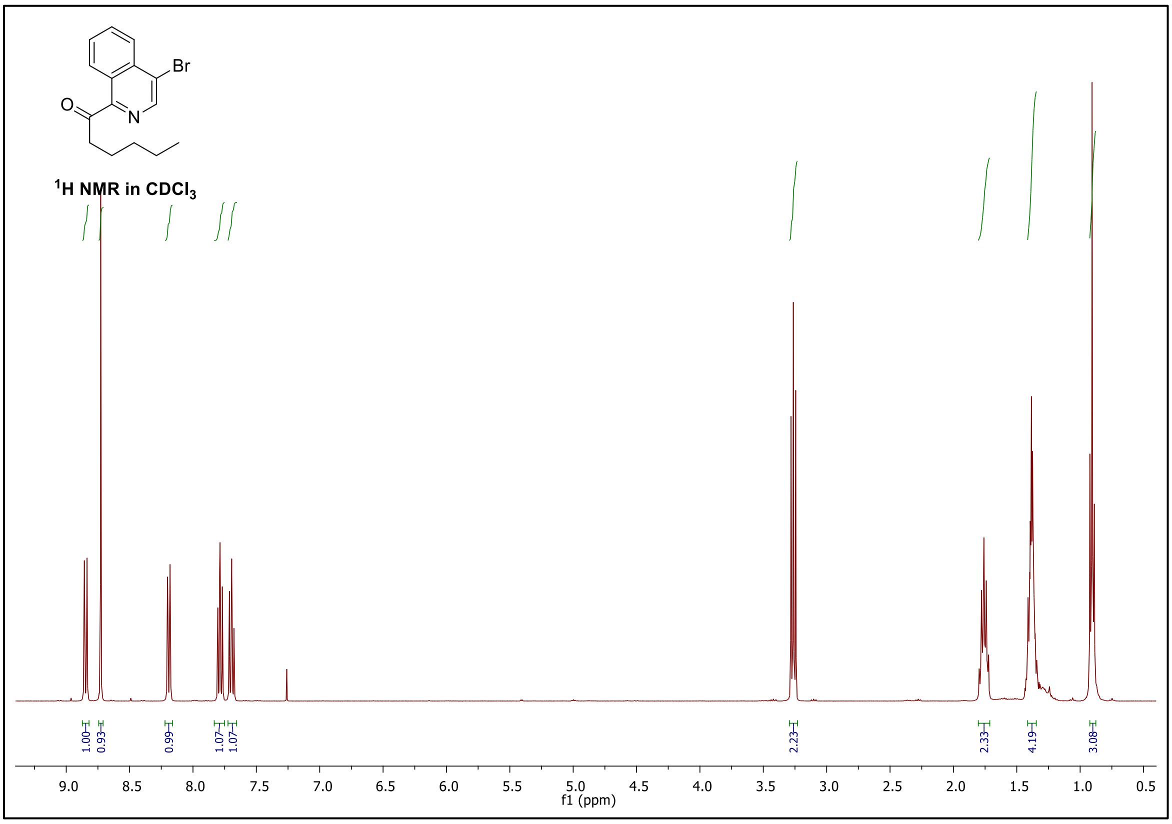

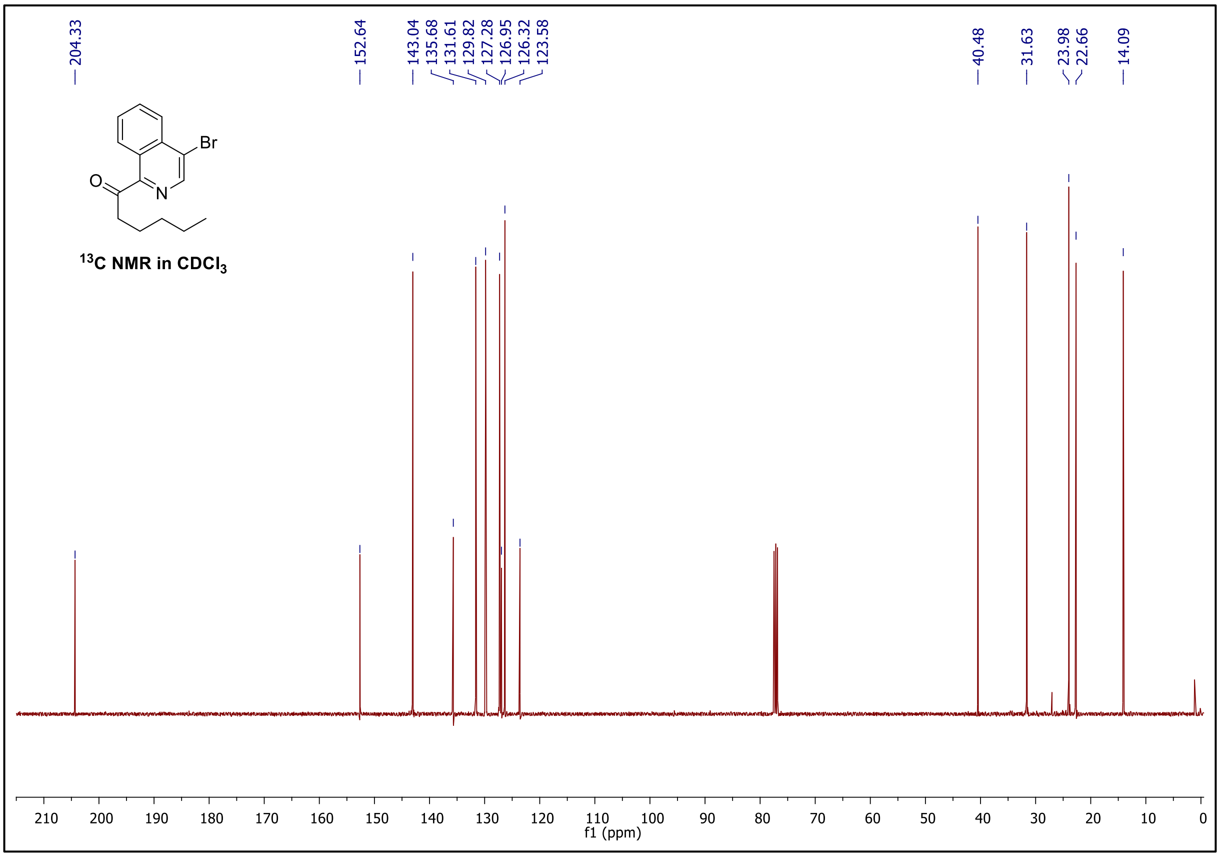

Compound 15

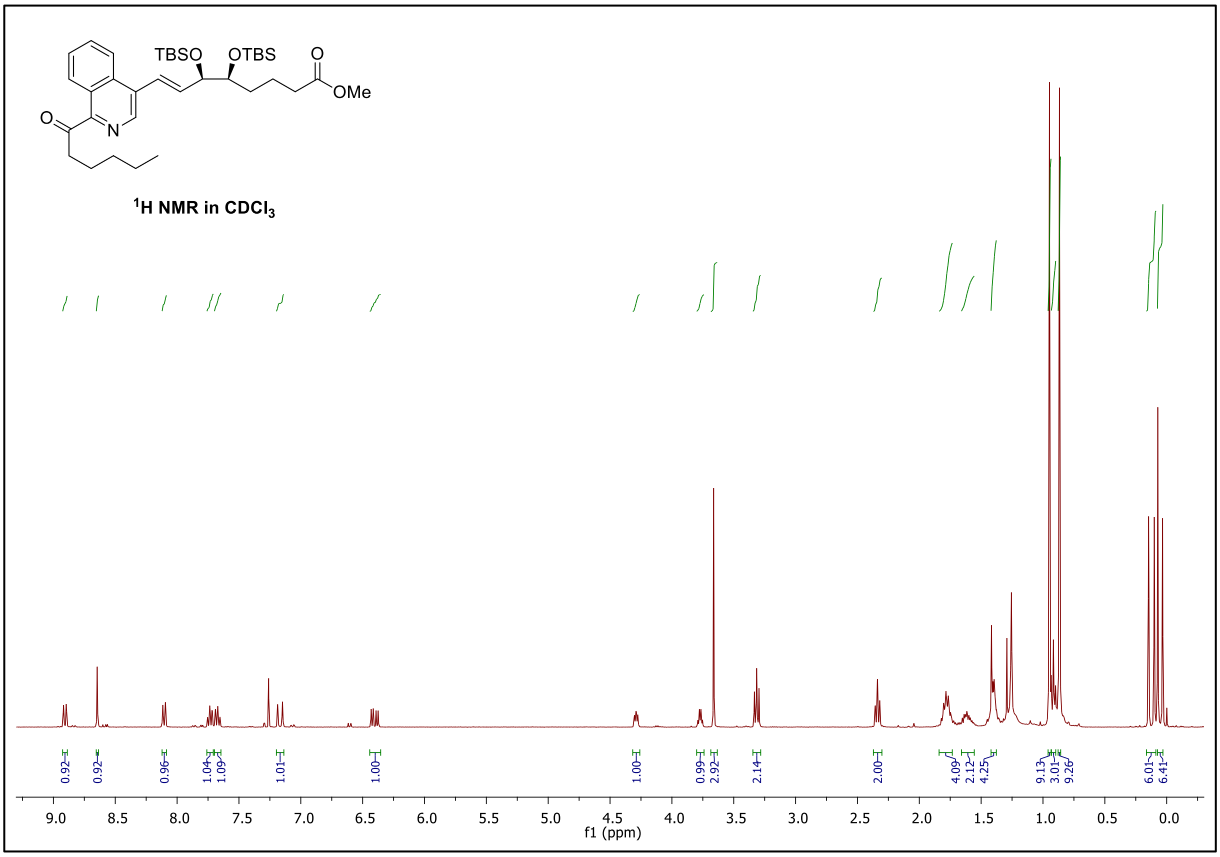

TBSO
OTBS
O
OMe
O
N
1H NMR in CDCl3
0.92
0.92
0.96
1.04
1.09
1.01
1.00
1.00
0.99
2.92
2.14
2.00
4.09
2.12
4.25
9.13
3.01
9.26
6.01
6.41
9.0
8.5
8.0
7.5
7.0
6.5
6.0
5.5
5.0
4.5
4.0
3.5
3.0
2.5
2.0
1.5
1.0
0.5
0.0
f1 (ppm)

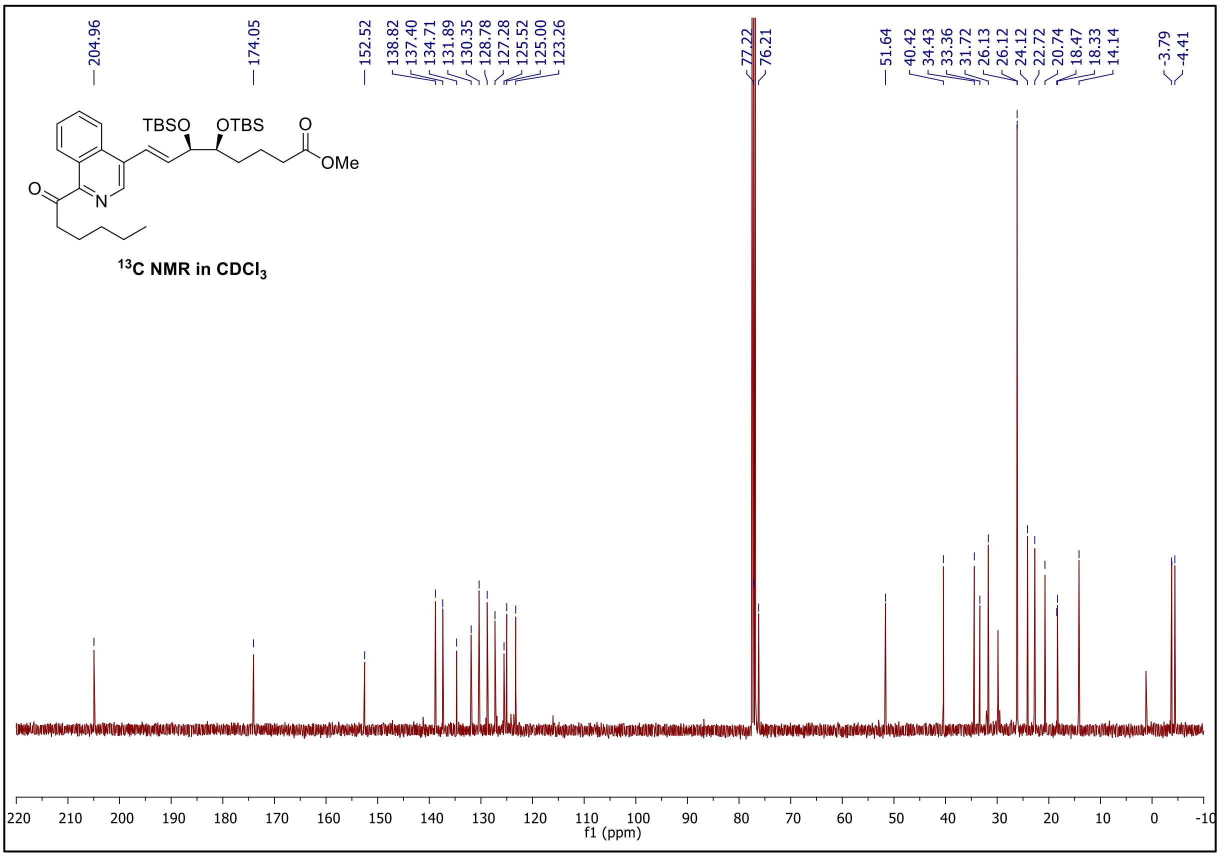

204.96
174.05
152.52
138.82
137.40
134.71
131.89
130.35
128.78
127.28
125.52
125.00
123.26
77.22
76.21
51.64
40.42
34.43
33.36
31.72
26.13
26.12
24.12
22.72
20.74
18.47
18.33
14.14
-3.79
-4.41
TBSO
OTBS
O
OMe
O
N
13C NMR in CDCl3
220
210
200
190
180
170
160
150
140
130
120
110
100
90
80
70
60
50
40
30
20
10
0
-10
f1 (ppm)

Compound (1*R*)-16

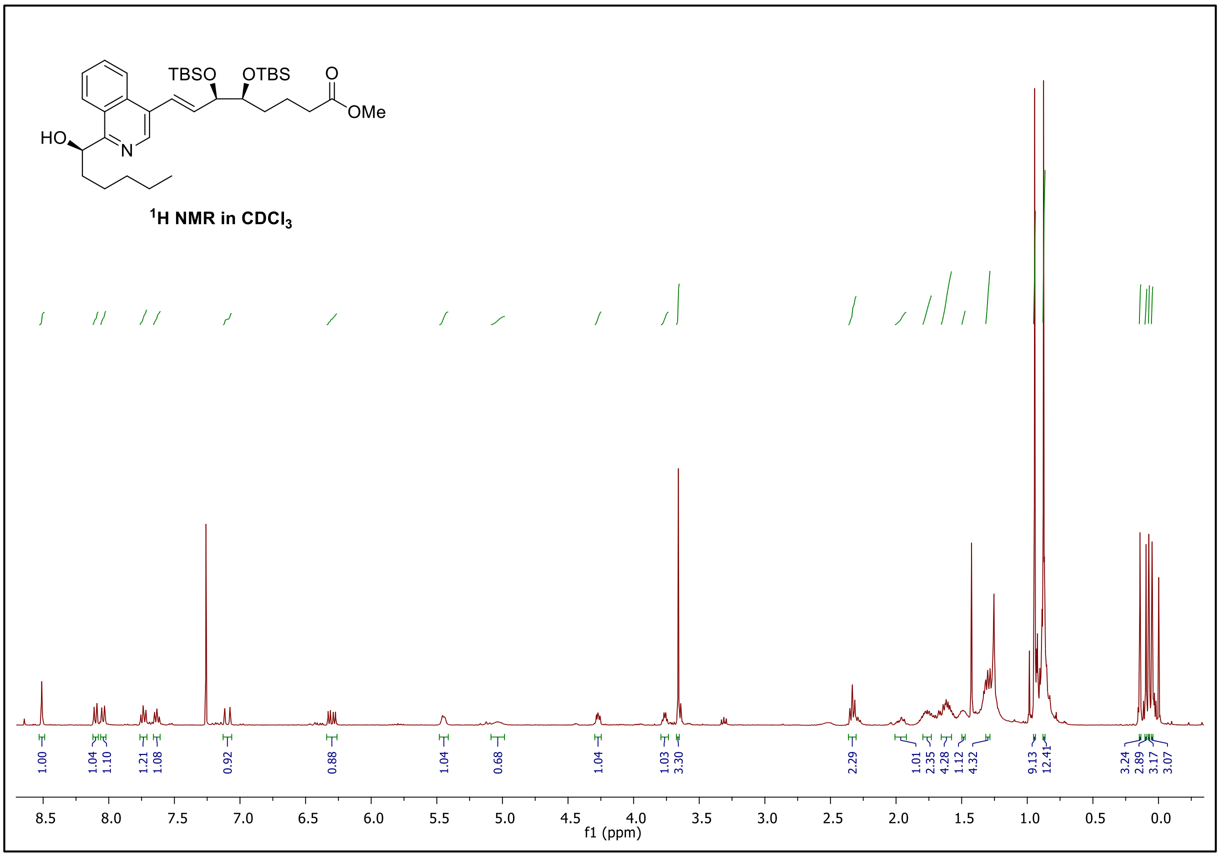

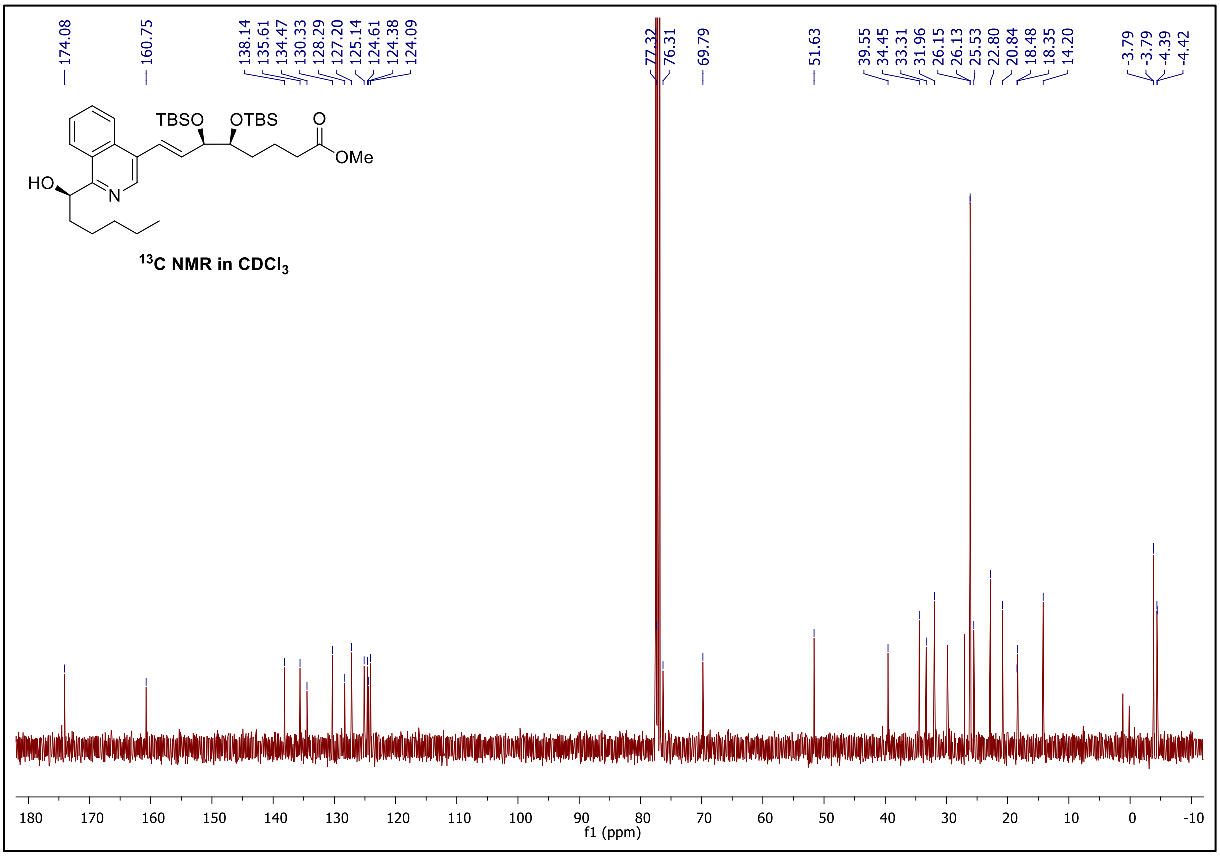

Compound (1*S*)-16

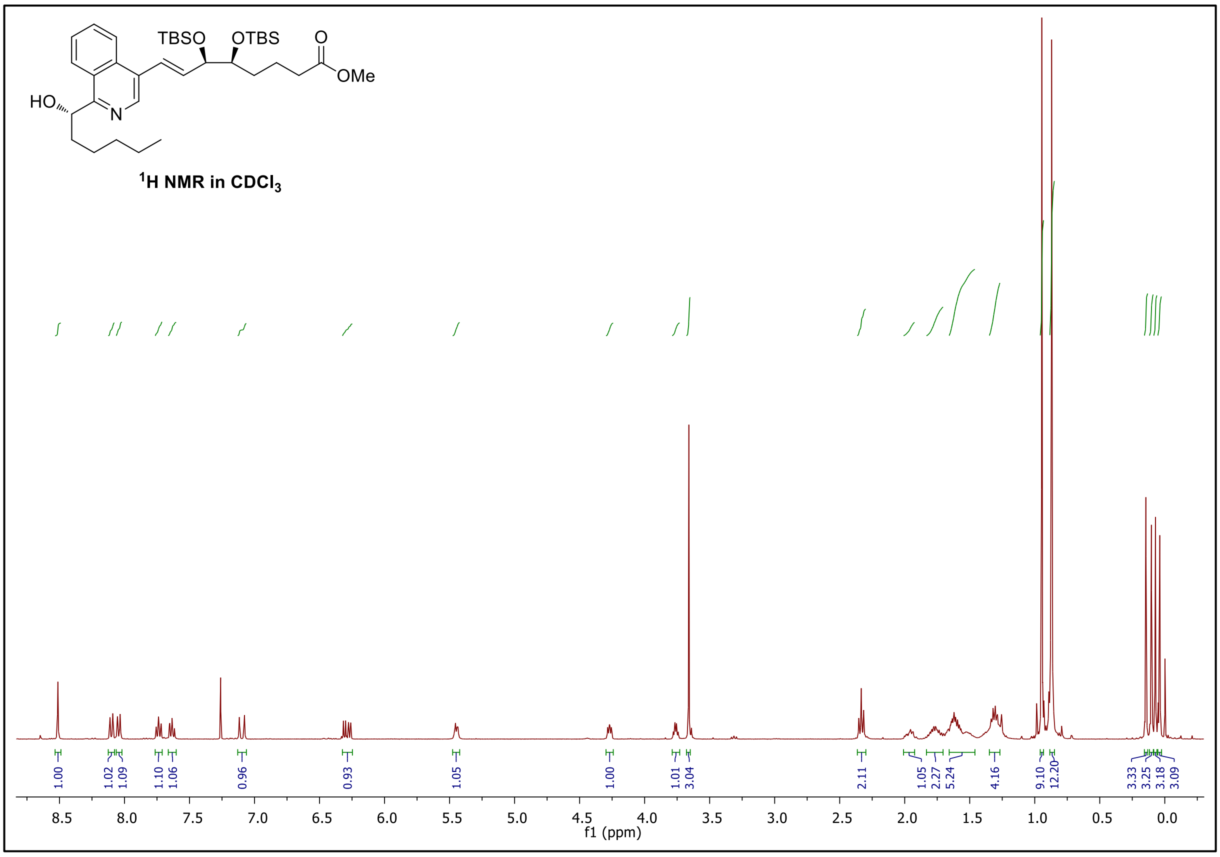

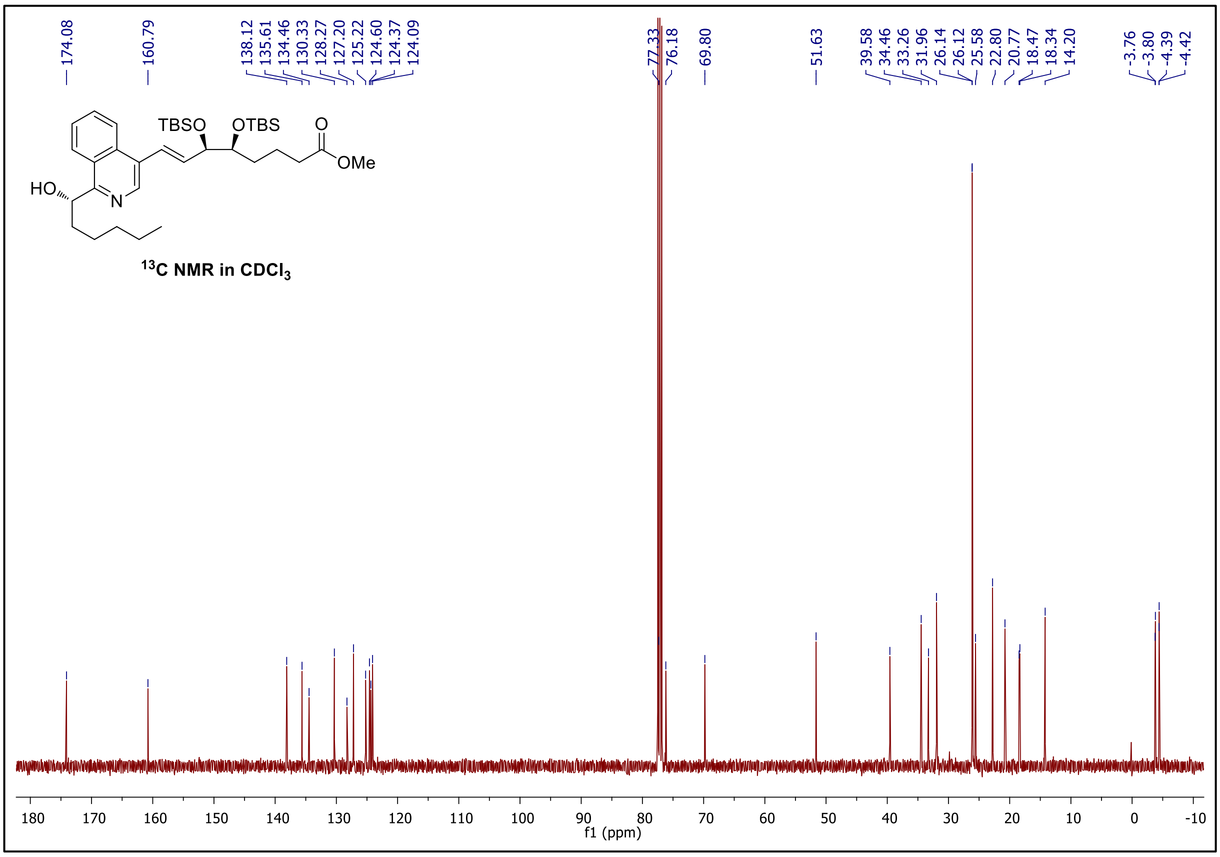

Compound (1*R*)-7

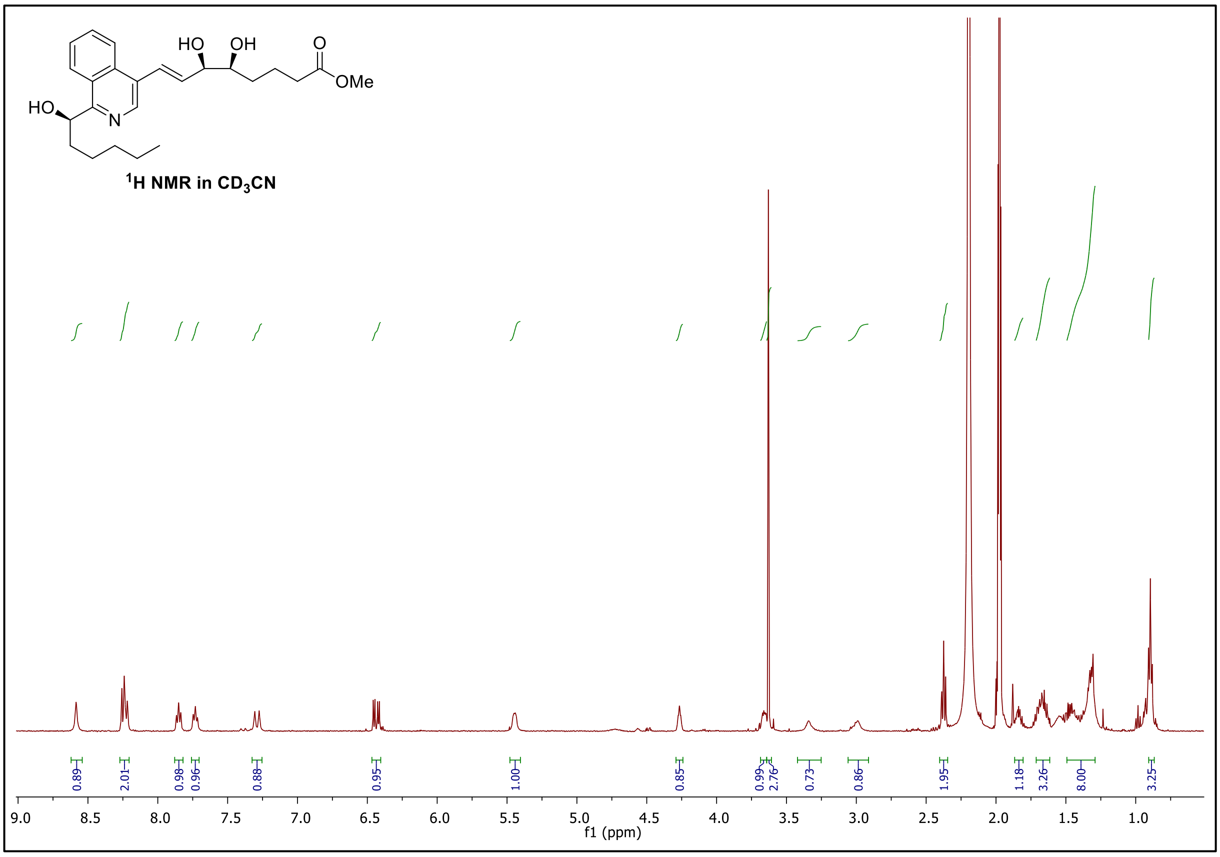

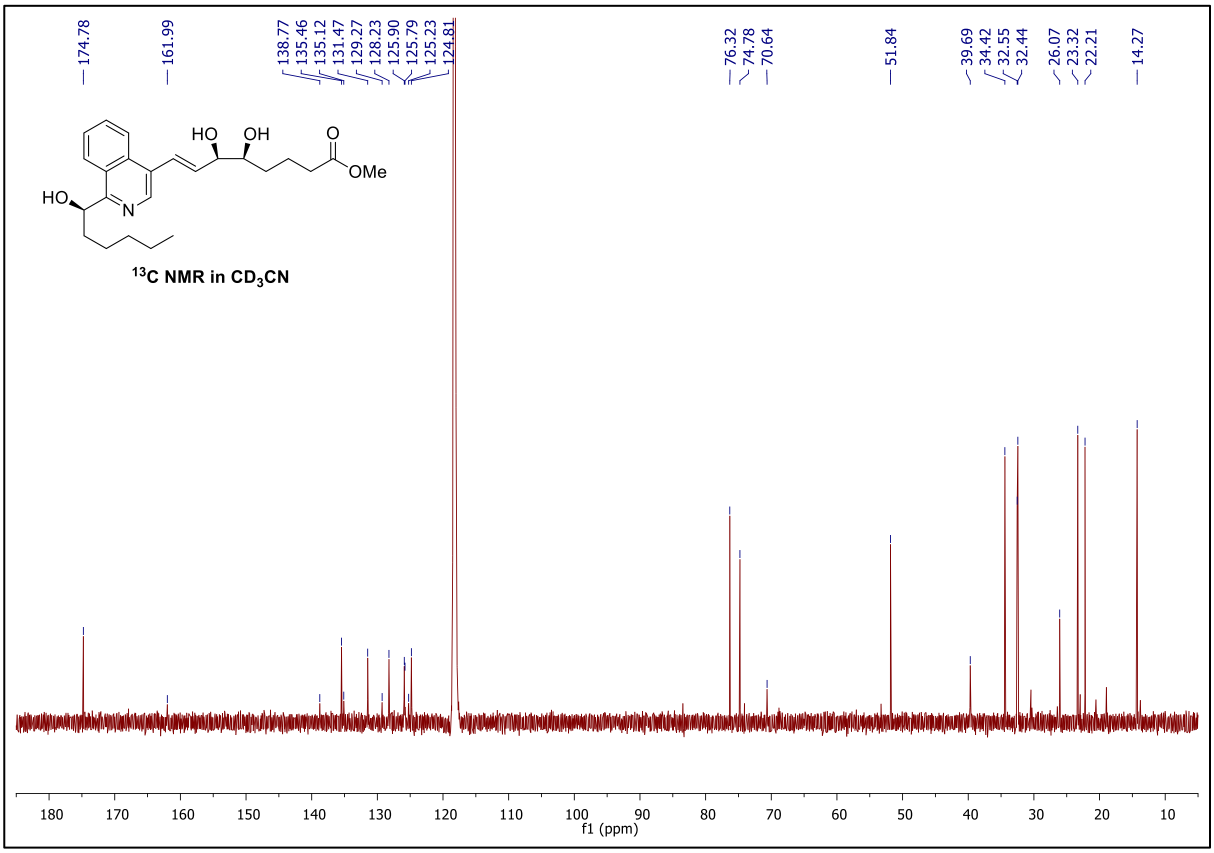

Compound (1*S*)-7

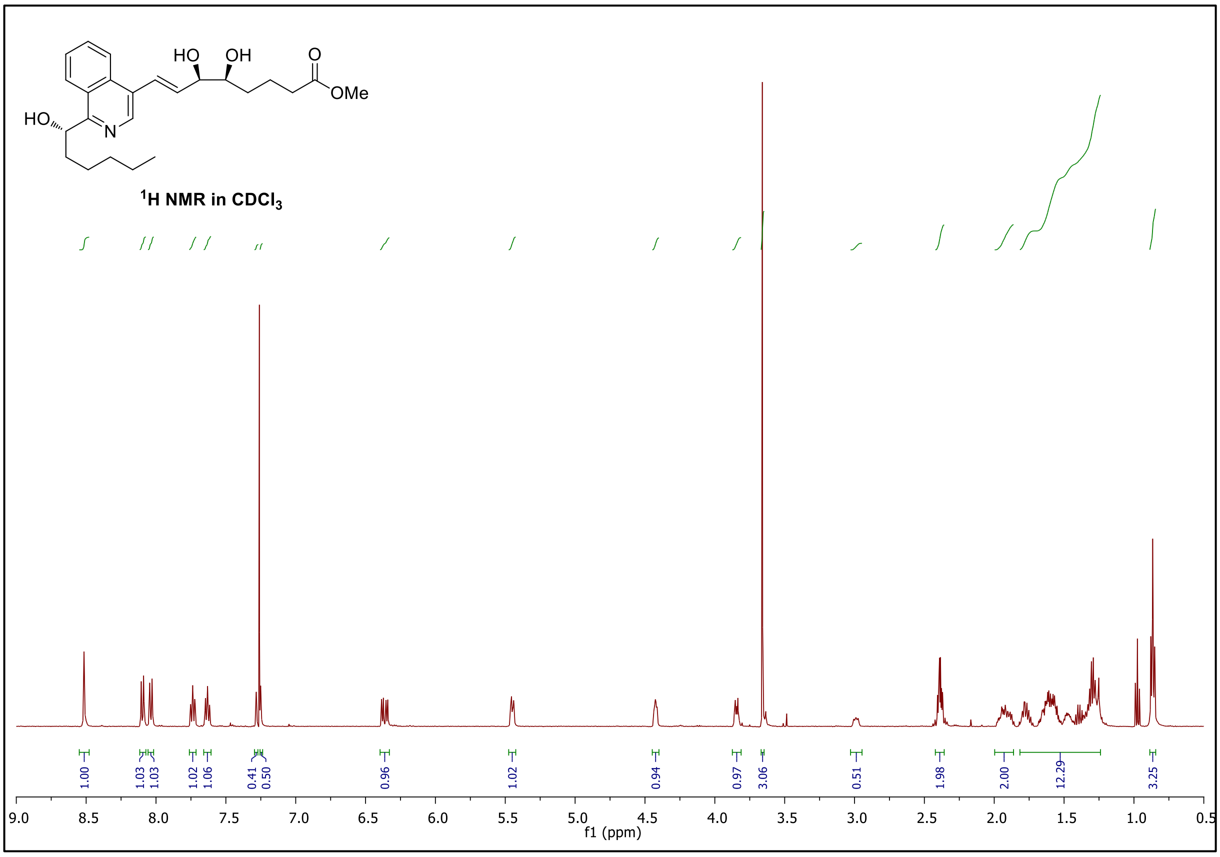

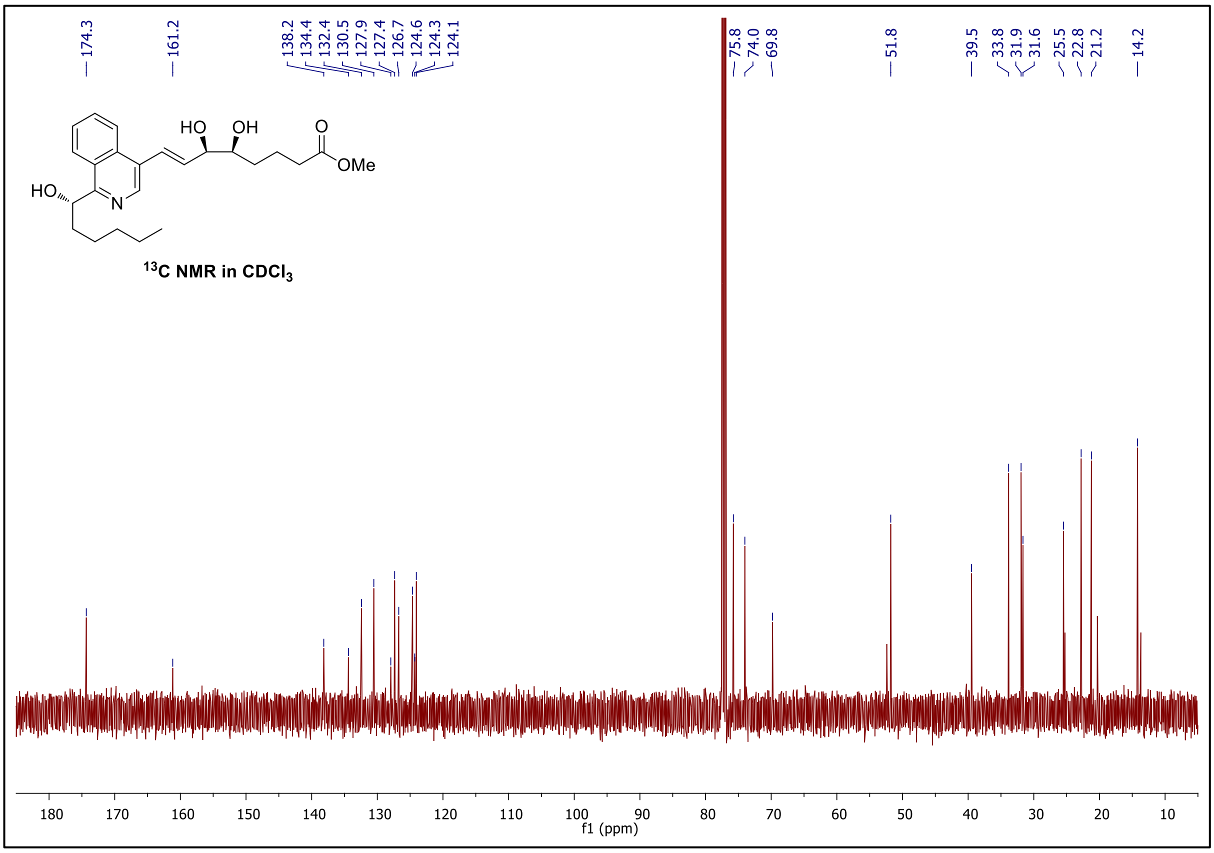

Compound (1*S*)-7-lac

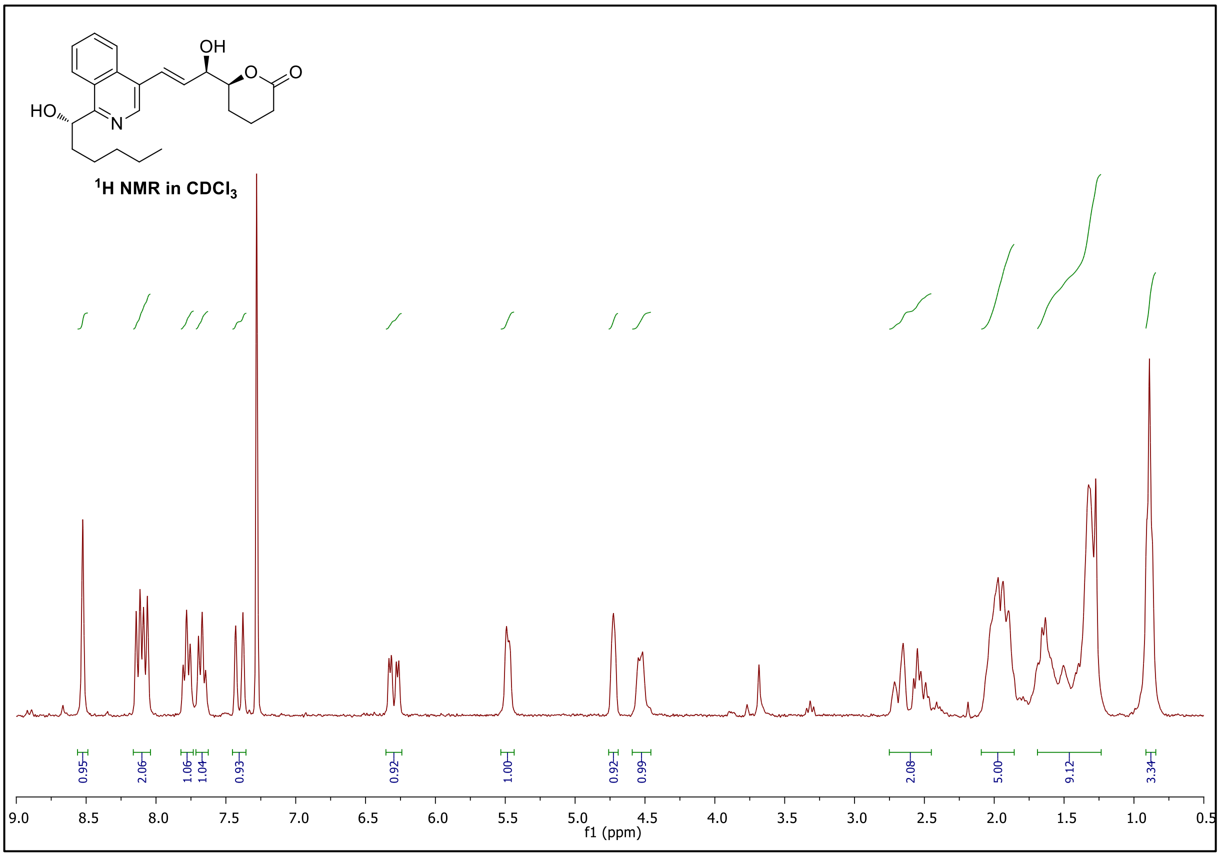

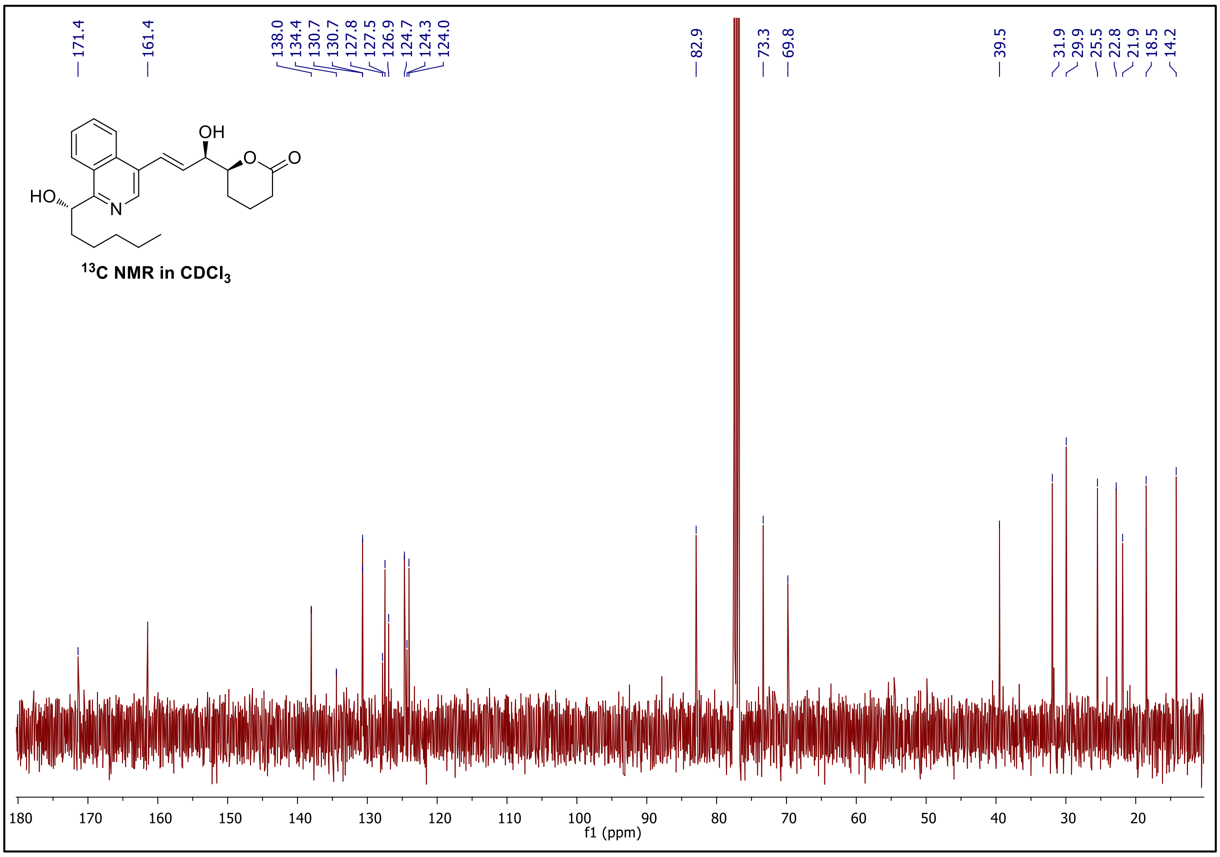

Compound 18

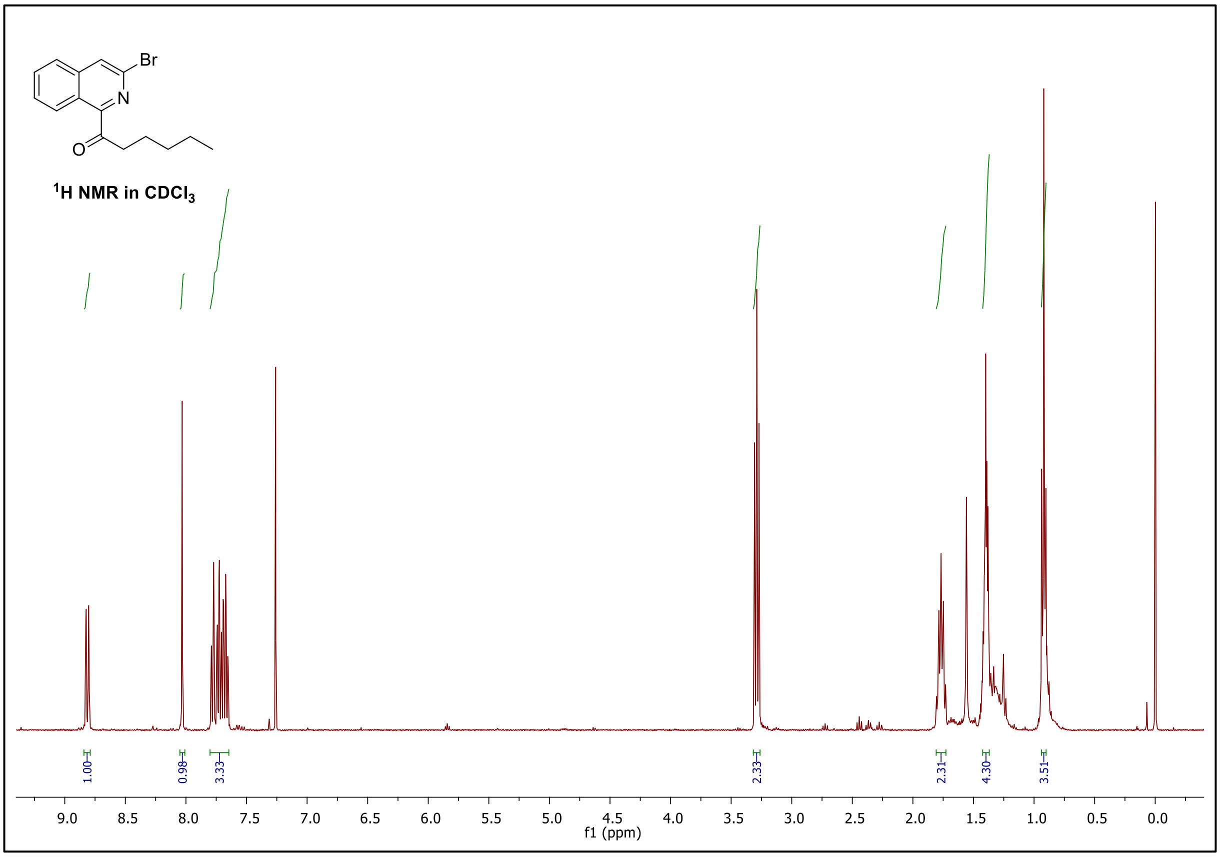
Br
N
O
¹H NMR in CDCl3
1.00
0.98
3.33
2.33
2.31
4.30
3.51
9.0 8.5 8.0 7.5 7.0 6.5 6.0 5.5 5.0 4.5 4.0 3.5 3.0 2.5 2.0 1.5 1.0 0.5 0.0
f1 (ppm)

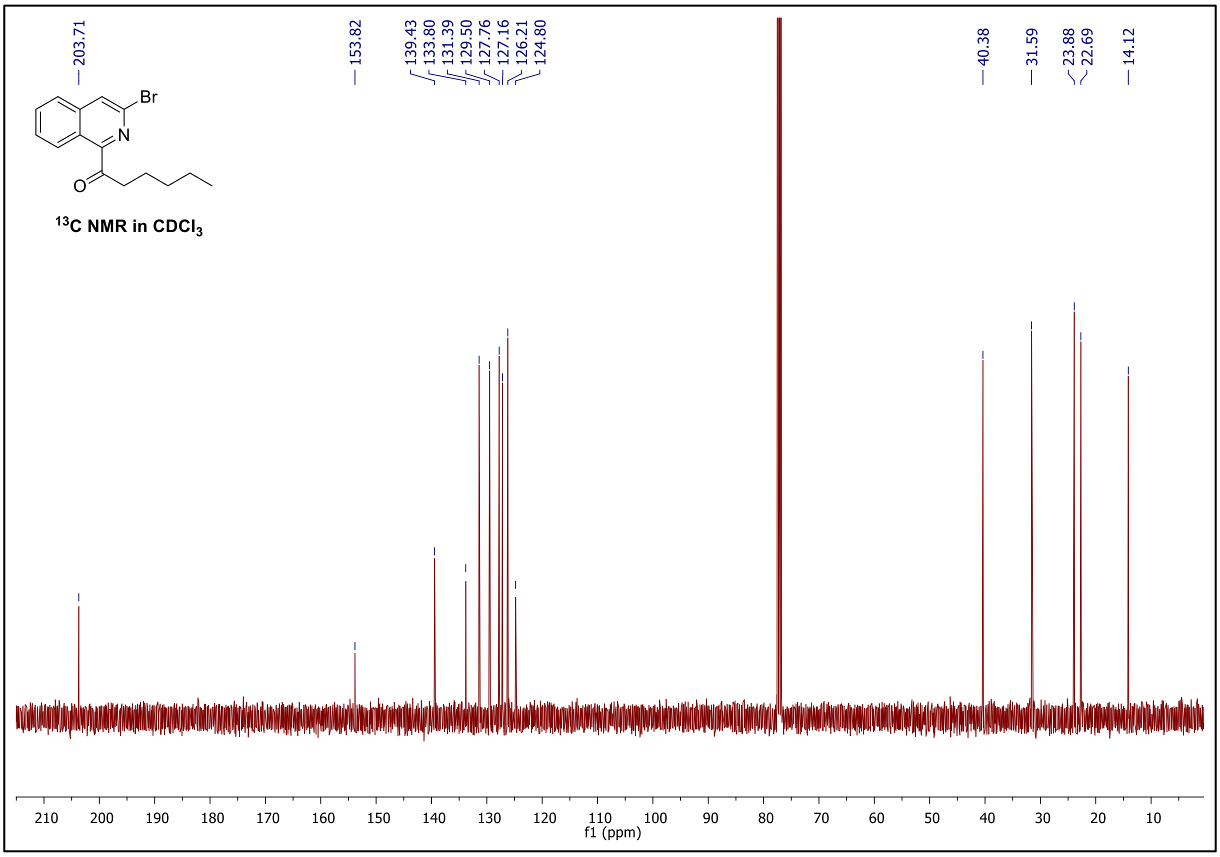
203.71
153.82
139.43
133.80
131.39
129.50
127.76
127.16
126.21
124.80
40.38
31.59
23.88
22.69
14.12
Br
N
O
¹³C NMR in CDCl3
210 200 190 180 170 160 150 140 130 120 110 100 90 80 70 60 50 40 30 20 10
f1 (ppm)

Compound 19

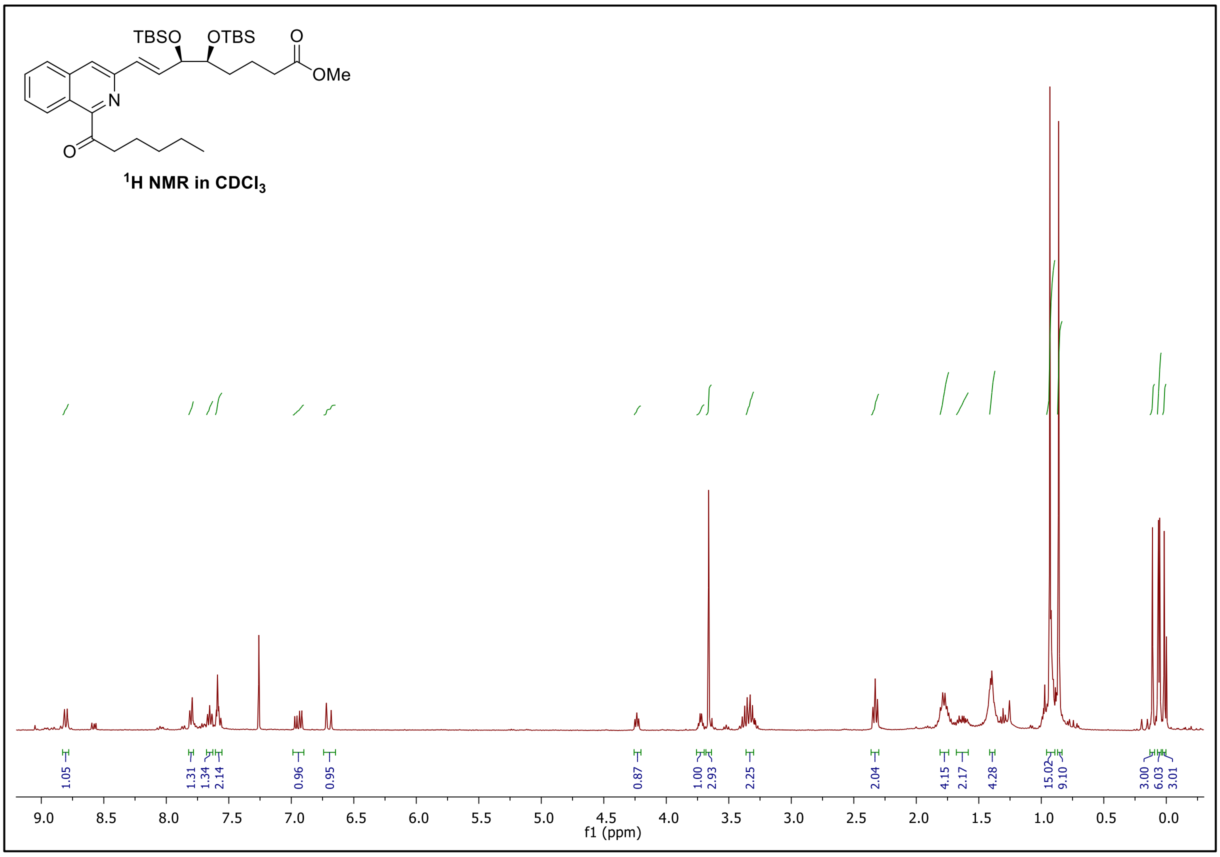

TBSO
OTBS
O
OMe
N
O
^{1}H NMR in CDCl$_3$
1.05
1.31
1.34
2.14
0.96
0.95
0.87
1.00
2.93
2.25
2.04
4.15
2.17
4.28
15.02
9.10
3.00
6.03
3.01
f1 (ppm)

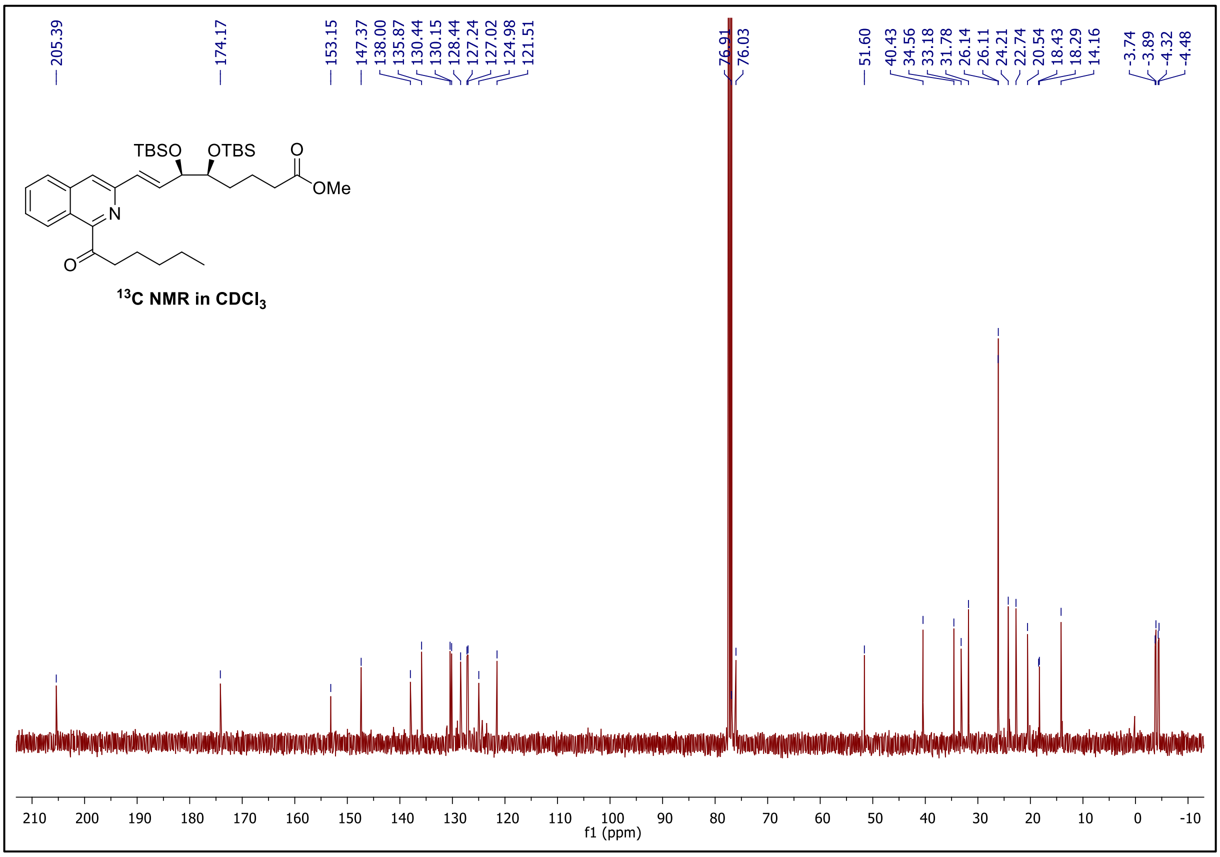

205.39
174.17
153.15
147.37
138.00
135.87
130.44
130.15
128.44
127.24
127.02
124.98
121.51
76.91
76.03
51.60
40.43
34.56
33.18
31.78
26.14
26.11
24.21
22.74
20.54
18.43
18.29
14.16
-3.74
-3.89
-4.32
-4.48
TBSO
OTBS
O
OMe
N
O
^{13}C NMR in CDCl$_3$
f1 (ppm)

Compound (1*R*)-8

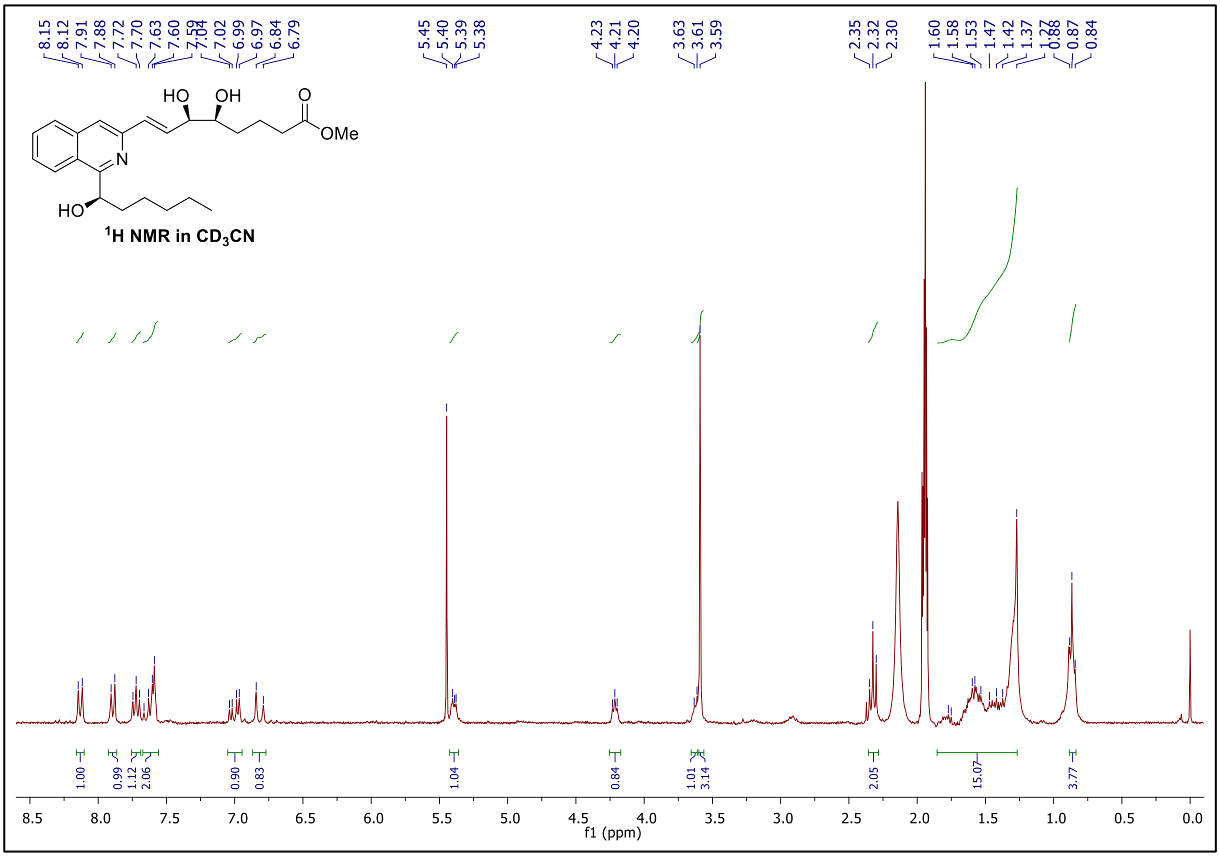

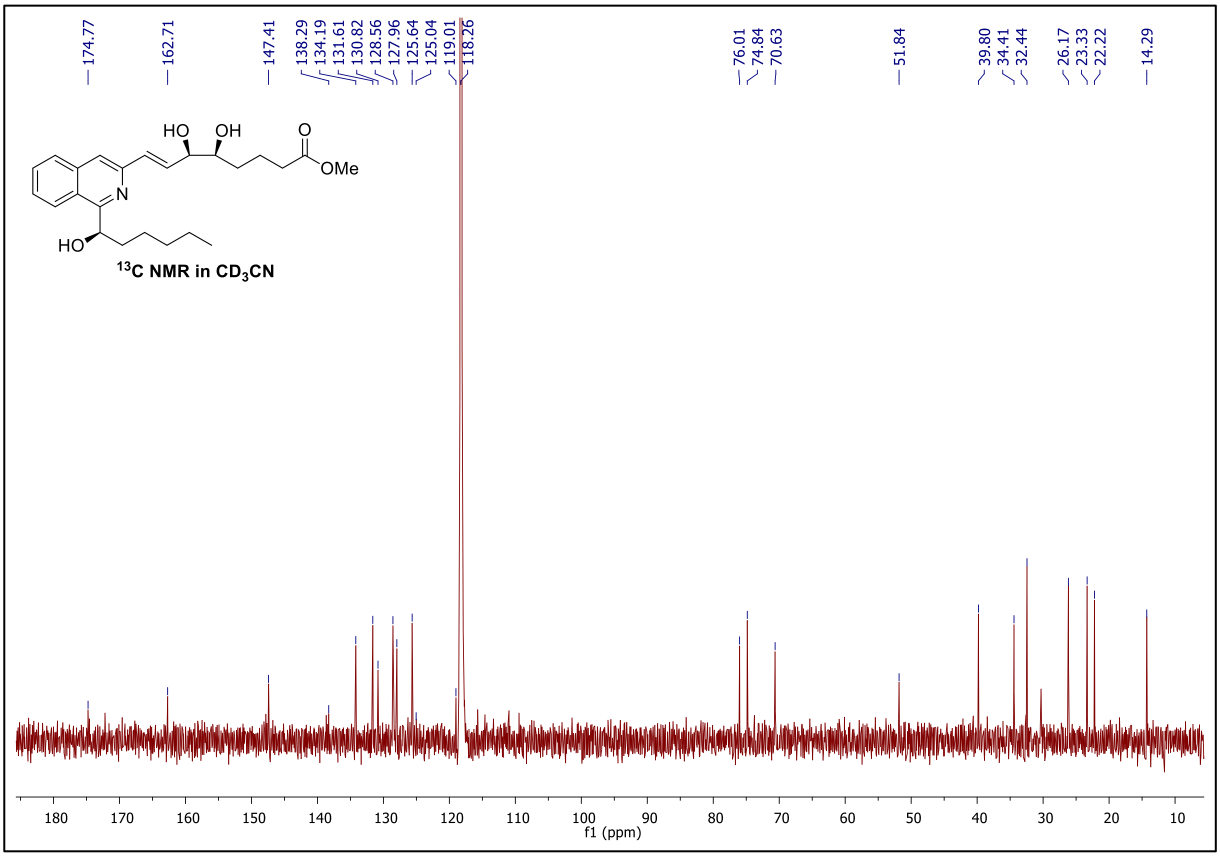

Compound (1*R*)-8-lac

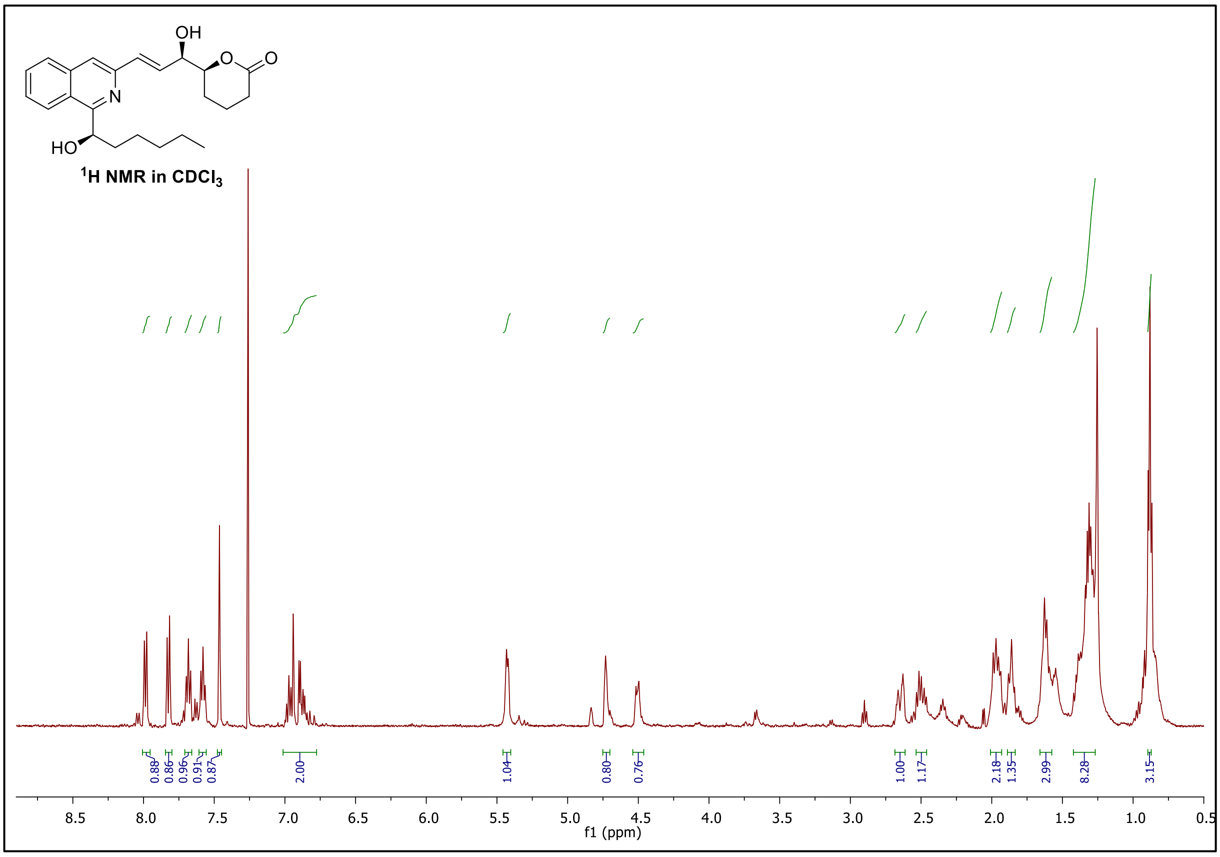

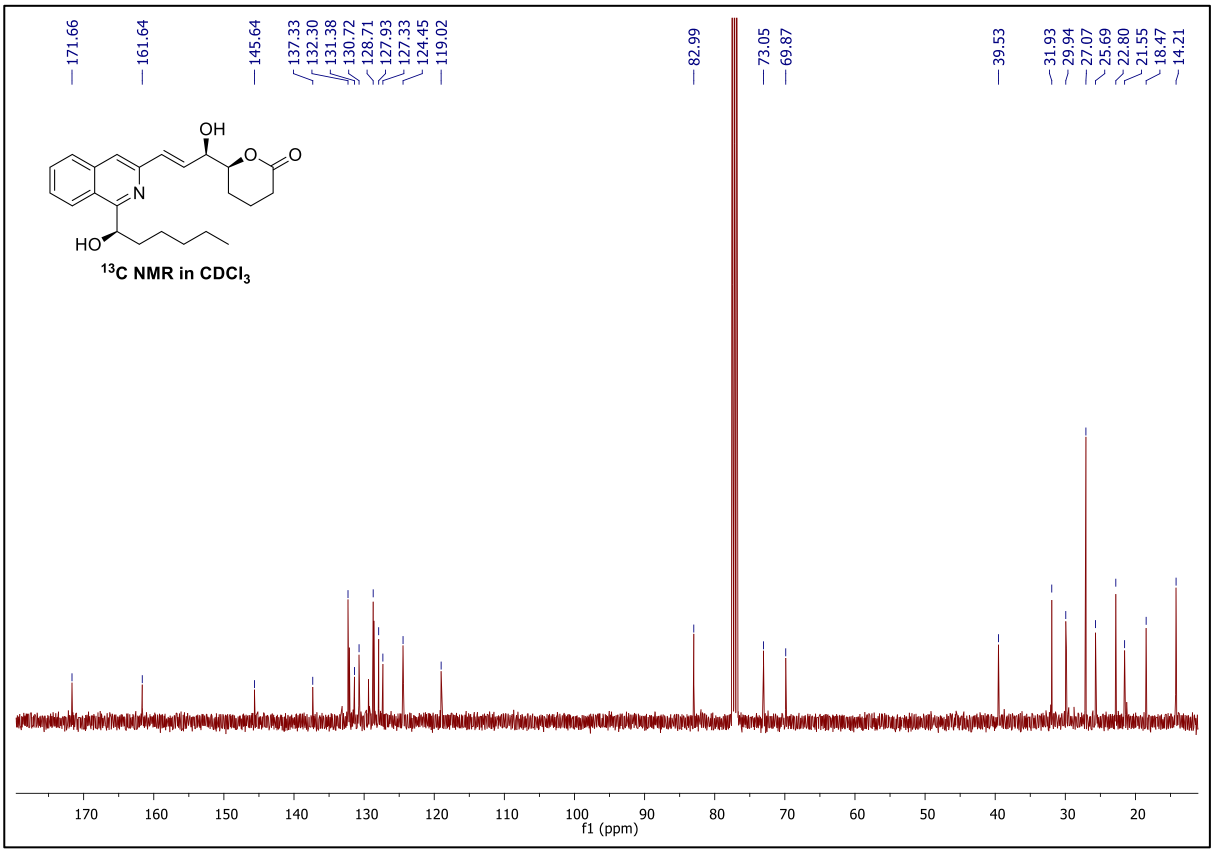

Compound (1*S*)-8

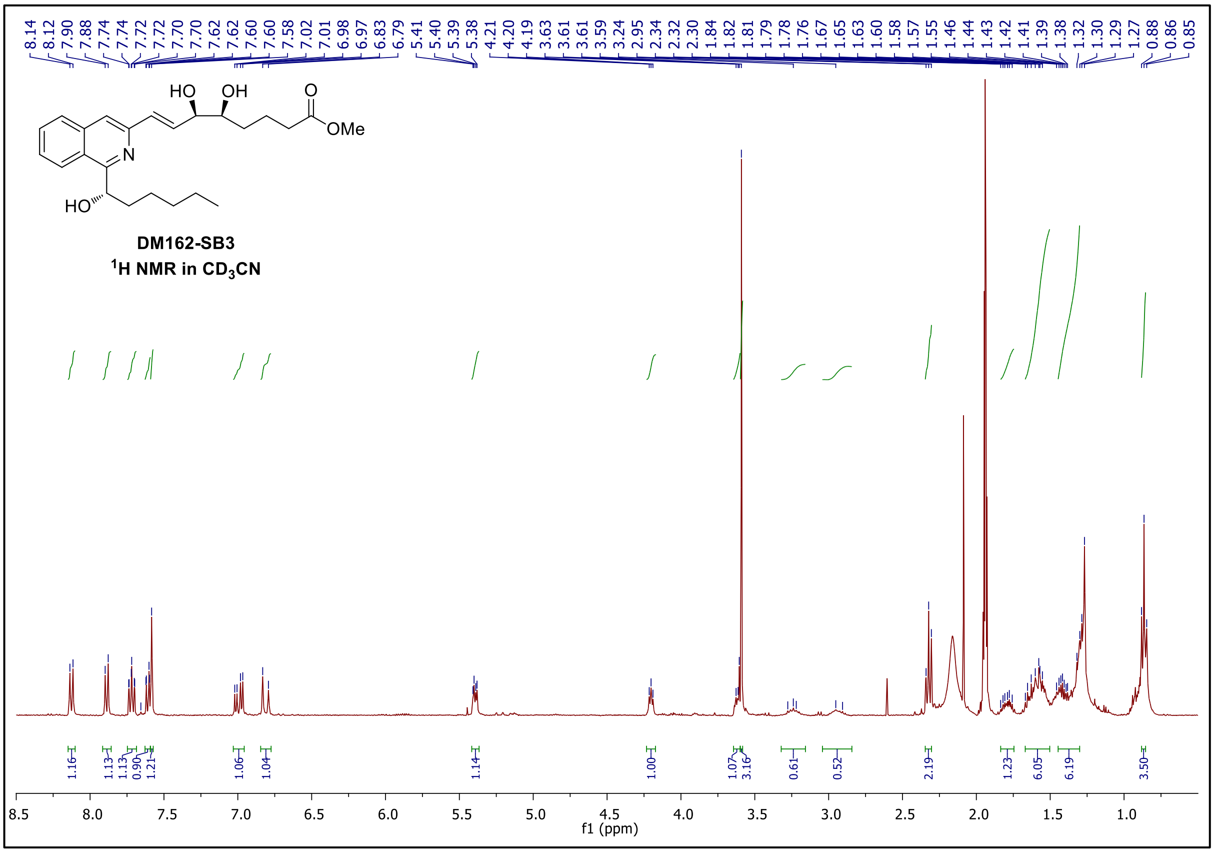

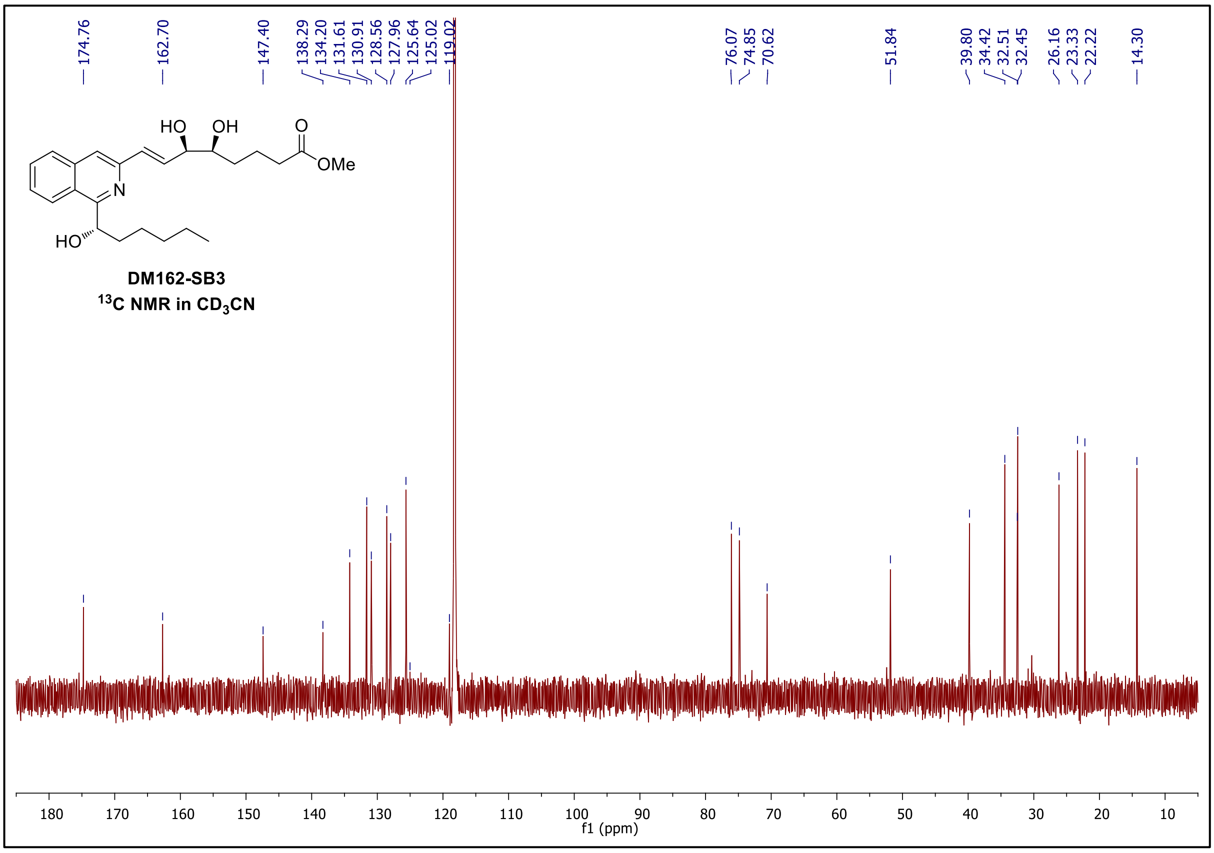

HPLC Chromatograms

Compound (1*R*)-20

96 % de

Sample Name:	DM127-T.Hyd4_A	Acquired By:	System
Sample Type:	Unknown	Sample Set Name:	DM127_THyd4_A_B
Vial:	1:E,7	Acq. Method Set:	DM_IPA15
Injection #:	1	Processing Method	DM67
Injection Volume:	10.00 ul	Channel Name:	219.4nm
Run Time:	4.0 Minutes	Proc. Chnl. Descr.:	PDA 219.4 nm
Date Acquired:	5/14/2014 12:28:33 PM IST		
Date Processed:	5/14/2014 12:55:11 PM IST		

Auto-Scaled Chromatogram

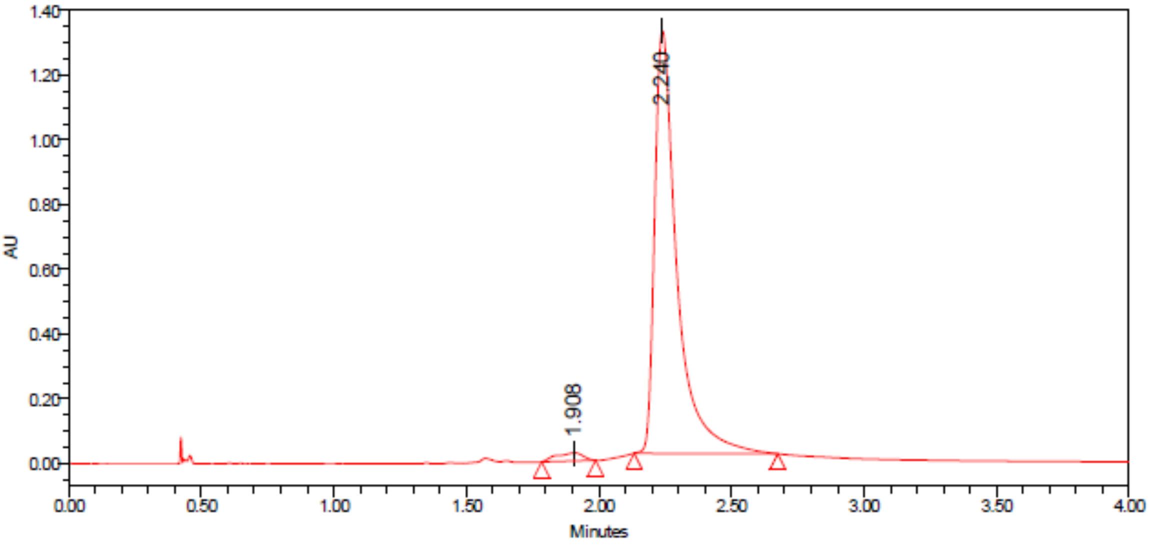

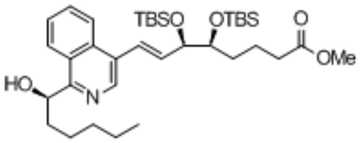

Peak Results

	Name	RT	Area	Height	% Area
1		1.908	175227	24887	2.23
2		2.240	7686597	1306808	97.77

Compound (1*S*)-20

88 % de

Sample Name:	DM86_IPA15	Acquired By:	System
Sample Type:	Unknown	Sample Set Name:	DM84_86
Vial:	1:D,8	Acq. Method Set:	DM_IPA15
Injection #:	1	Processing Method	DM67
Injection Volume:	10.00 ul	Channel Name:	219.7nm
Run Time:	4.0 Minutes	Proc. Chnl. Descr.:	PDA 219.7 nm
Date Acquired:	9/25/2013 6:36:42 PM IST		
Date Processed:	9/25/2013 6:41:56 PM IST		

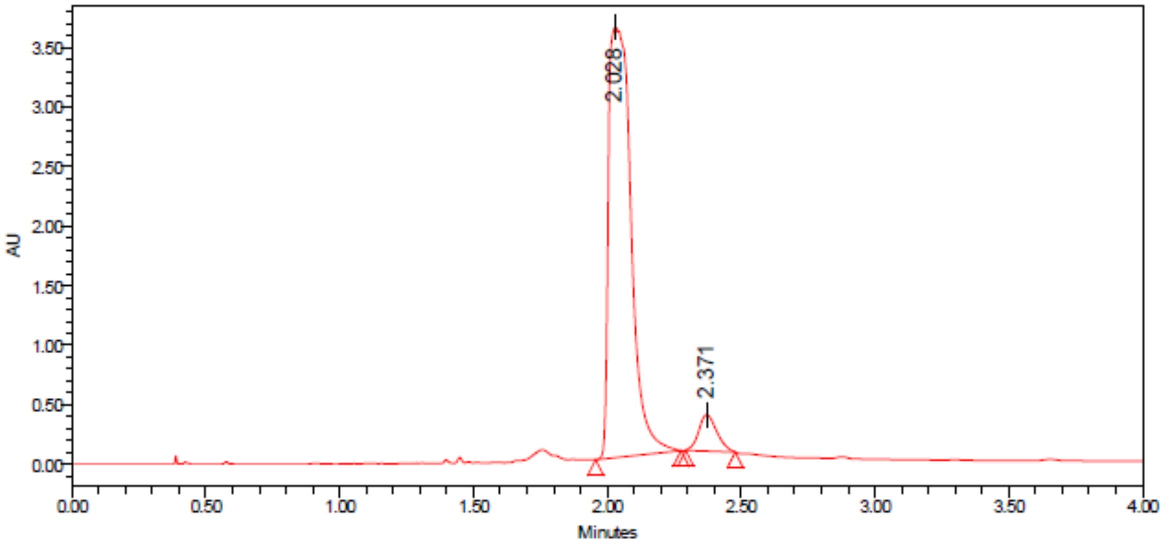

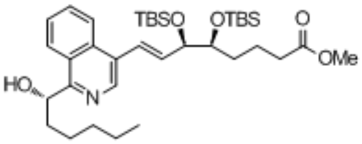

Peak Results

	Name	RT	Area	Height	% Area
1		2.028	21124701	3617603	93.61
2		2.371	1441717	306028	6.39

Compound (1*R*)-7

95 % purity @ 235 nm

Sample Name:	DM164_AB_70	Acquired By:	System
Sample Type:	Unknown	Sample Set Name:	DM164_AB_Screen
Vial:	1:F,8	Acq. Method Set:	generic_chiral_sol1
Injection #:	1	Processing Method	DM
Injection Volume:	3.00 ul	Channel Name:	235.0nm
Run Time:	20.0 Minutes	Proc. Chnl. Descr.:	PDA 235.0 nm
Date Acquired:	11/20/2014 12:24:28 PM GMT		
Date Processed:	12/2/2014 5:38:10 PM GMT		

Auto-Scaled Chromatogram

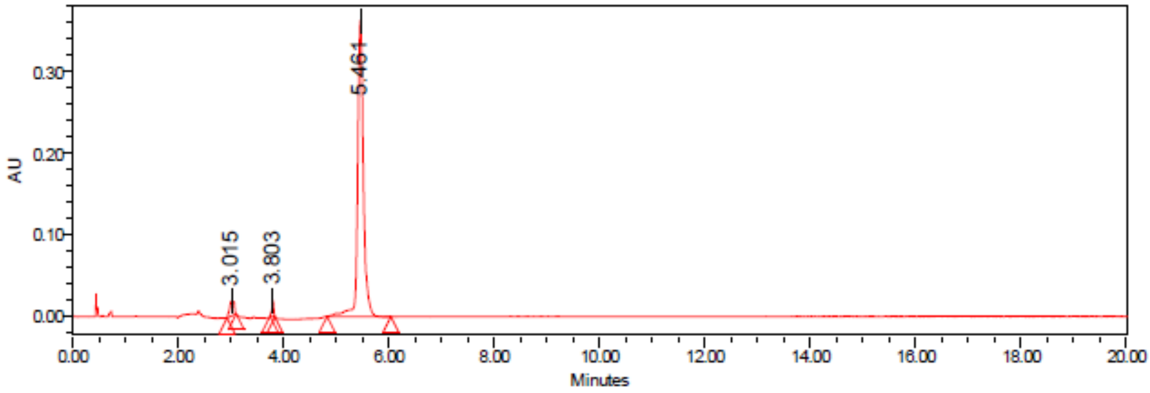

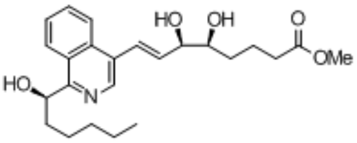

Peak Results

	Name	RT	Area	Height	% Area
1		3.015	11064(	18443	3.78
2		3.803	46947	19683	1.60
3		5.461	2772640	363900	94.62

Compound (1*R*)-7

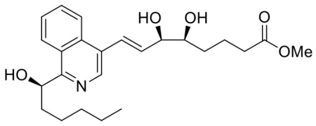

96 % purity @ 285 nm

Sample Name:	DM164_AB_70	Acquired By:	System
Sample Type:	Unknown	Sample Set Name:	DM164_AB_Screen
Vial:	1:F,8	Acq. Method Set:	generic_chiral_sol1
Injection #:	1	Processing Method	DM
Injection Volume:	3.00 ul	Channel Name:	285.0nm
Run Time:	20.0 Minutes	Proc. Chnl. Descr.:	PDA 285.0 nm
Date Acquired:	11/20/2014 12:24:28 PM GMT		
Date Processed:	9/14/2015 10:02:57 AM IST		

Auto-Scaled Chromatogram

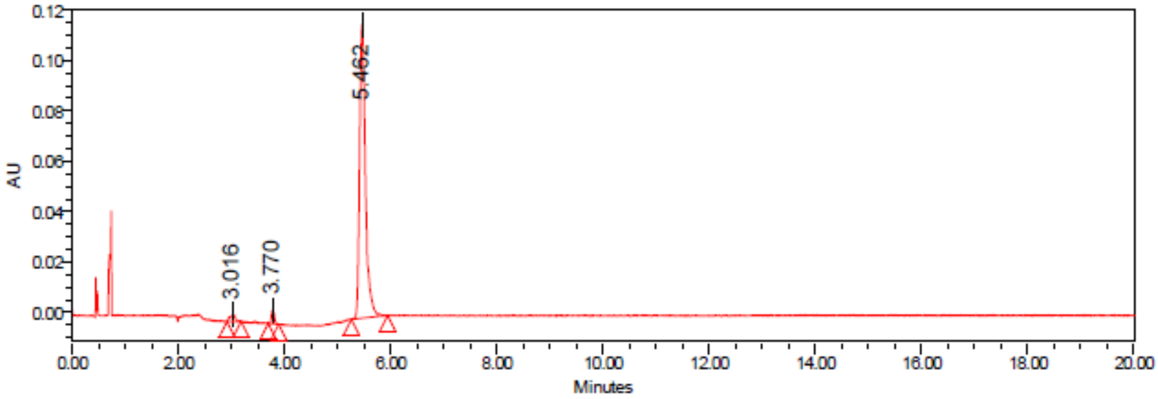

Peak Results

	Name	RT	Area	Height	% Area
1		3.016	17972	2520	1.93
2		3.770	20783	5282	2.23
3		5.462	892685	116555	95.84

Compound (1*S*)-7

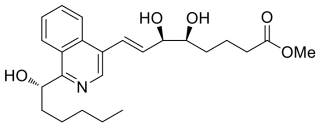

98 % purity at 220 nm

Sample Name:	DM178_SB4_NH3	Acquired By:	System
Sample Type:	Unknown	Sample Set Name:	DM178_SB4_NH3
Vial:	1:B,1	Acq. Method Set:	generic_chiral_sol1
Injection #:	1	Processing Method	DM
Injection Volume:	5.00 ul	Channel Name:	220.0nm
Run Time:	8.0 Minutes	Proc. Chnl. Descr.:	PDA 220.0 nm
Date Acquired:	2/4/2015 2:44:58 PM GMT		
Date Processed:	11/23/2015 12:09:45 PM GMT		

Auto-Scaled Chromatogram

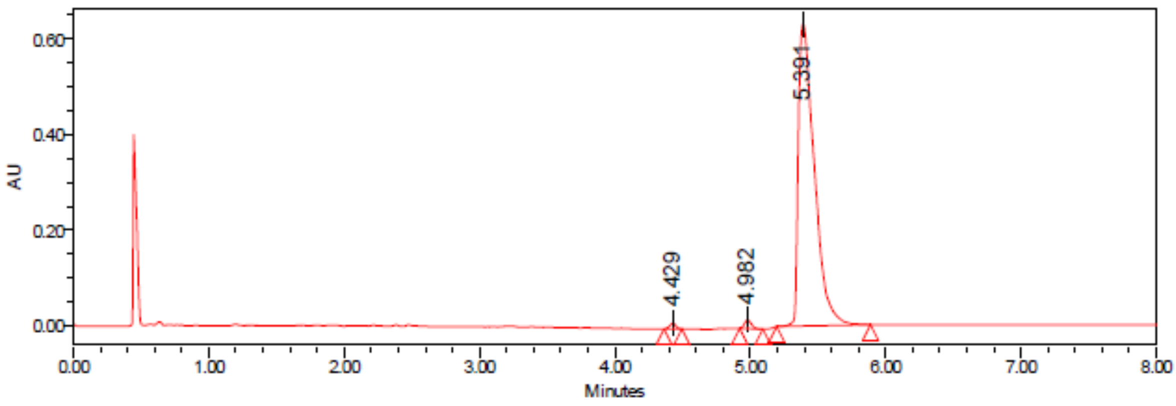

Peak Results

	Name	RT	Area	Height	% Area
1		4.429	38049	12475	0.72
2		4.982	63749	18209	1.21
3		5.391	5186338	632076	98.07

Compound (1*S*)-7

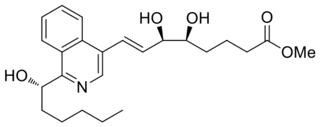

98 % purity at 240 nm

Sample Name:	DM178_SB4_NH3	Acquired By:	System
Sample Type:	Unknown	Sample Set Name:	DM178_SB4_NH3
Vial:	1:B,1	Acq. Method Set:	generic_chiral_sol1
Injection #:	1	Processing Method	DM
Injection Volume:	5.00 ul	Channel Name:	240.0nm
Run Time:	8.0 Minutes	Proc. Chnl. Descr.:	PDA 240.0 nm
Date Acquired:	2/4/2015 2:44:58 PM GMT		
Date Processed:	11/23/2015 12:11:52 PM GMT		

Auto-Scaled Chromatogram

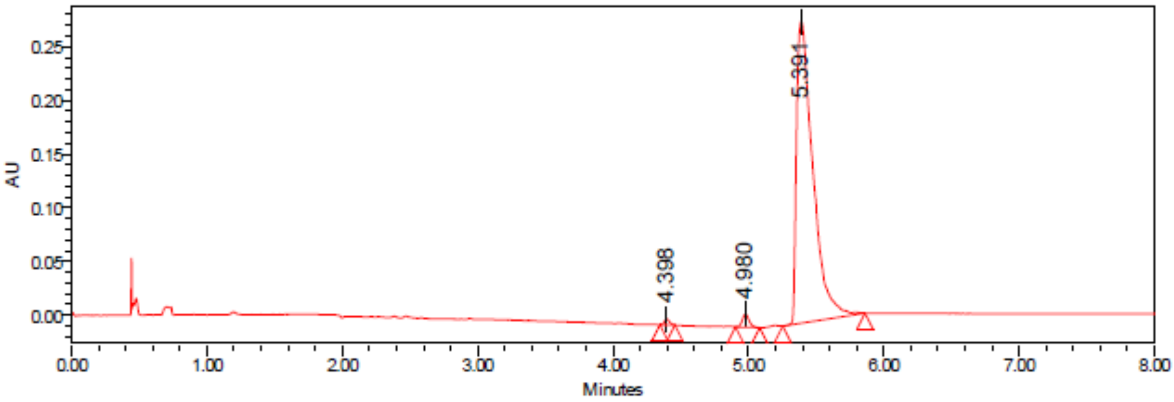

Peak Results

	Name	RT	Area	Height	% Area
1		4.398	14376	5555	0.60
2		4.980	41940	12063	1.74
3		5.391	2358820	280760	97.67

Compound (1*S*)-7-lac

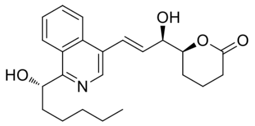

96 % purity at 240 nm

Sample Name:	DM178_SA4_initial	Acquired By:	System
Sample Type:	Unknown	Sample Set Name:	DM178_SpA4_3
Vial:	1:C,1	Acq. Method Set:	generic_chiral_sol1
Injection #:	1	Processing Method	DM164_A
Injection Volume:	5.00 ul	Channel Name:	240.0nm
Run Time:	7.0 Minutes	Proc. Chnl. Descr.:	PDA 240.0 nm
Date Acquired:	2/10/2015 6:40:54 PM GMT		
Date Processed:	11/23/2015 12:23:16 PM GMT		

Auto-Scaled Chromatogram

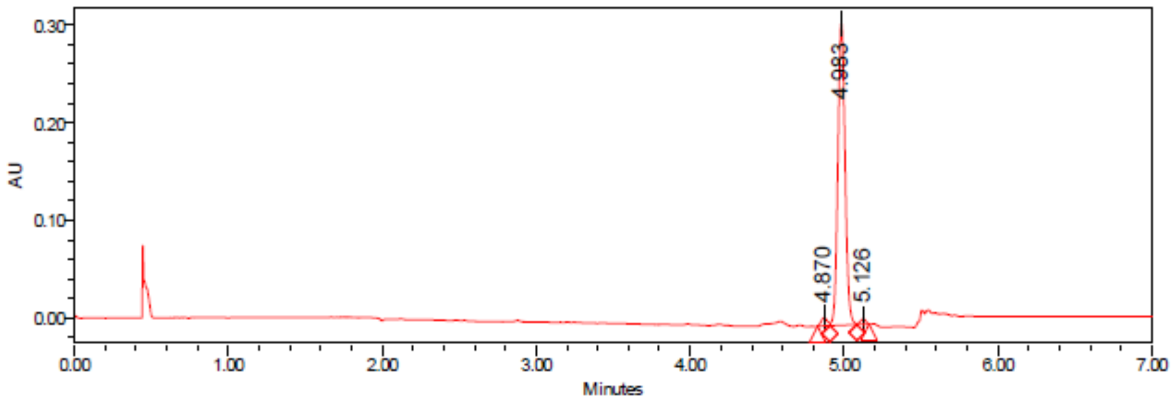

Peak Results

	Name	RT	Area	Height	% Area
1		4.870	23092	8334	2.23
2		4.983	1000464	309503	96.40
3		5.126	14294	5512	1.38

Compound (1*S*)-7-lac

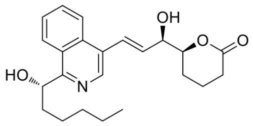

96 % purity at 340 nm

Sample Name:	DM178_SA4_initial	Acquired By:	System
Sample Type:	Unknown	Sample Set Name:	DM178_SpA4_3
Vial:	1:C,1	Acq. Method Set:	generic_chiral_sol1
Injection #:	1	Processing Method	DM164_A
Injection Volume:	5.00 ul	Channel Name:	340.0nm
Run Time:	7.0 Minutes	Proc. Chnl. Descr.:	PDA 340.0 nm
Date Acquired:	2/10/2015 6:40:54 PM GMT		
Date Processed:	11/23/2015 12:27:22 PM GMT		

Auto-Scaled Chromatogram

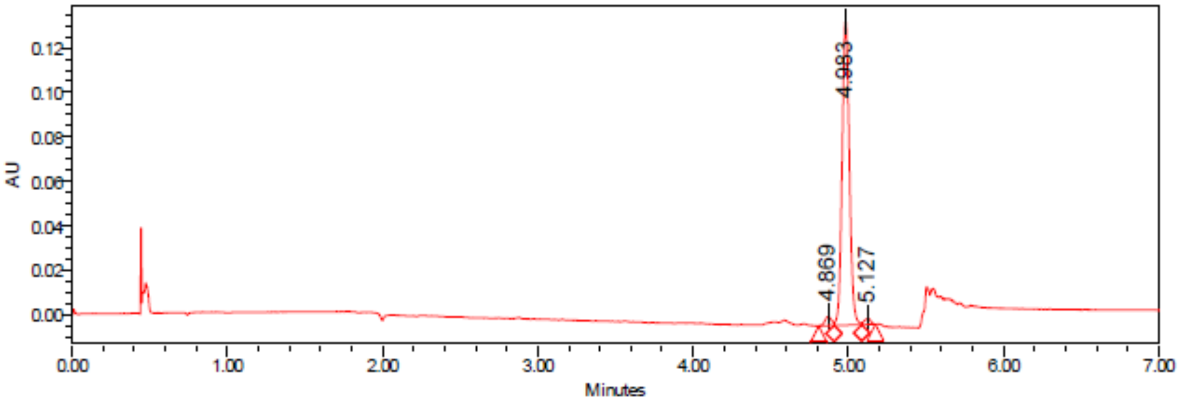

Peak Results

	Name	RT	Area	Height	% Area
1		4.869	11837	4026	2.55
2		4.983	443605	136595	95.74
3		5.127	7885	2768	1.70

Compound (1*R*)-20

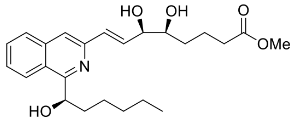

94 % de

Data Filename	: DM146-R_H3_932014_4.lcd		
Method Filename	: Isocratic-98.5_1.5_1.0ml_10min.lcm		
Batch Filename	: DM125_RacH3_RH3_3.lcb		
Vial #	: 1-77	Sample Type	: Unknown
Injection Volume	: 5 uL		
Date Acquired	: 9/3/2014 11:15:48 AM	Acquired by	: System Administrator
Date Processed	: 9/3/2014 11:25:51 AM	Processed by	: System Administrator

<Chromatogram>

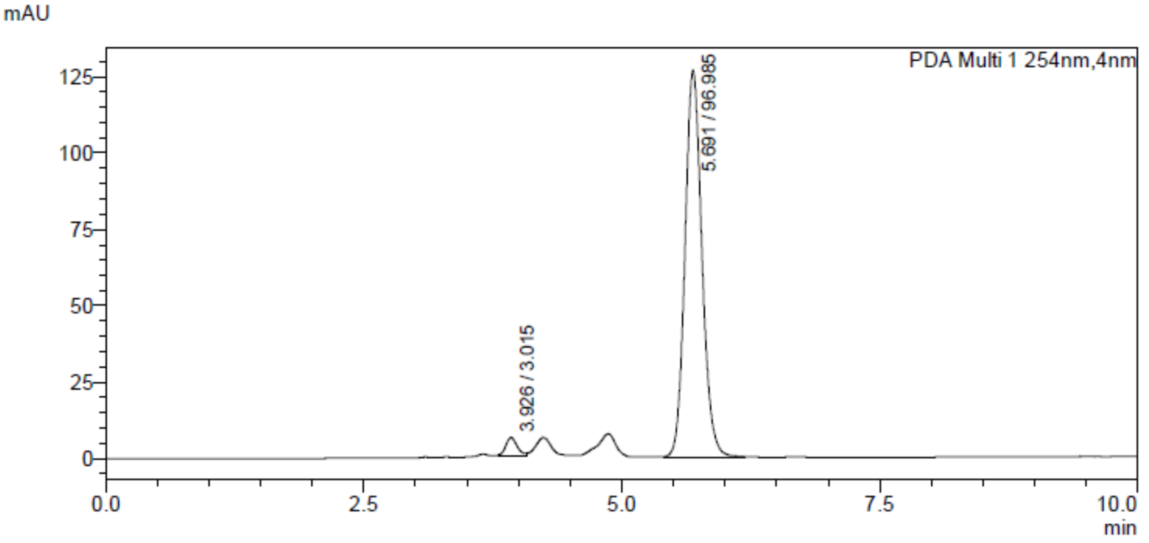

<Peak Table>

PDA Ch1 254nm

Peak#	Ret. Time	Area	Area%	Height
1	3.926	44813	3.015	5898
2	5.691	1441329	96.985	126477
Total		1486142	100.000	132375

Compound (1*S*)-20

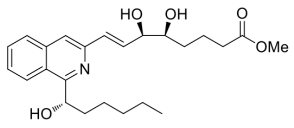

94% de

Data Filename	: DMSH3_932015_3.lcd		
Method Filename	: Isocratic-98.5_1.5_1.0ml_10min.lcm		
Batch Filename	: DM200RacH3.lcb		
Vial #	: 1-77	Sample Type	: Unknown
Injection Volume	: 1 uL		
Date Acquired	: 9/3/2015 7:17:49 PM	Acquired by	: System Administrator
Date Processed	: 9/3/2015 7:27:52 PM	Processed by	: System Administrator

<Chromatogram>

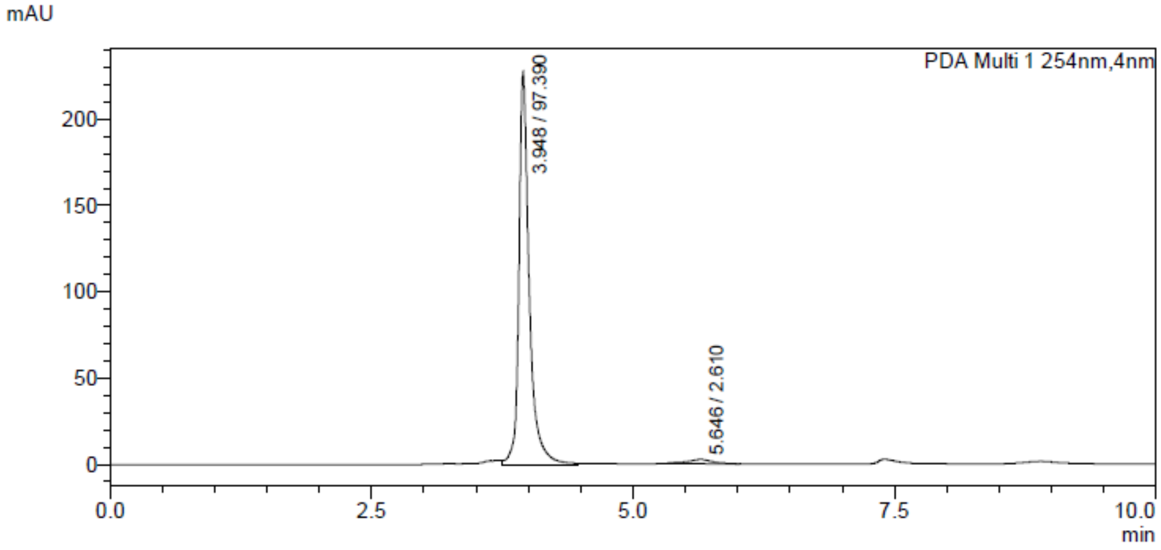

<Peak Table>

PDA Ch1 254nm

Peak#	Ret. Time	Area	Area%	Height
1	3.948	1568077	97.390	227512
2	5.646	42022	2.610	2405
Total		1610098	100.000	229918

Compound (1*R*)-8

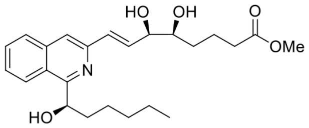

97 % purity at 235 nm

Sample Name:	DM163-pRB3	Acquired By:	System
Sample Type:	Unknown	Sample Set Name:	DM_SA3_pRB3
Vial:	1:B,7	Acq. Method Set:	generic_chiral_sol1
Injection #:	1	Processing Method	DM
Injection Volume:	10.00 ul	Channel Name:	235.0nm
Run Time:	10.0 Minutes	Proc. Chnl. Descr.:	PDA 235.0 nm
Date Acquired:	12/5/2014 5:16:08 PM GMT		
Date Processed:	1/6/2016 11:02:25 AM GMT		

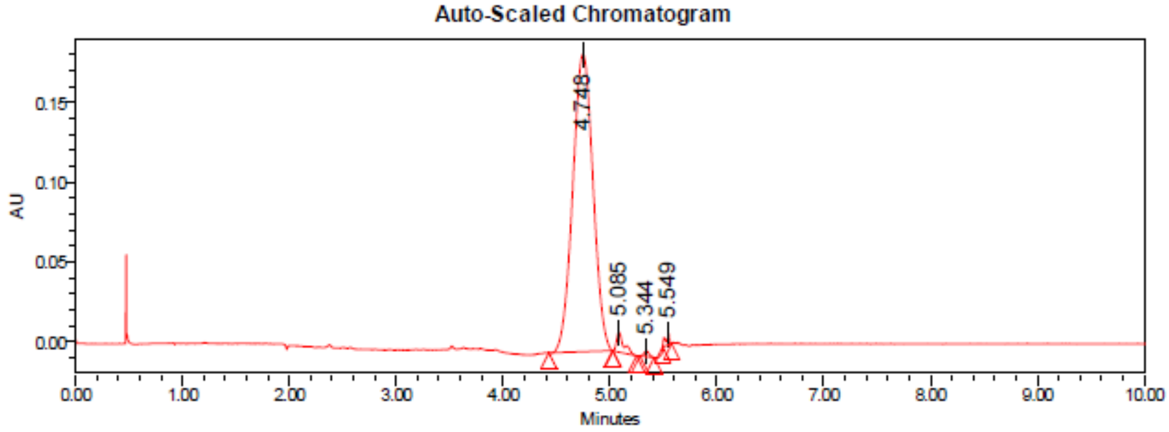

Peak Results

	Name	RT	Area	Height	% Area
1		4.748	2462974	186590	96.63
2		5.085	57725	12502	2.26
3		5.344	1145[illegible]	3618	0.45
4		5.549	16611	7037	0.65

Compound (1*R*)-8

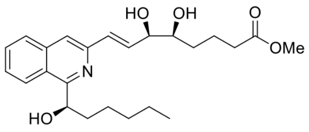

97 % purity at 250 nm

Sample Name:	DM163-pRB3	Acquired By:	System
Sample Type:	Unknown	Sample Set Name:	DM_SA3_pRB3
Vial:	1:B,7	Acq. Method Set:	generic_chiral_sol1
Injection #:	1	Processing Method	DM
Injection Volume:	10.00 ul	Channel Name:	250.0nm
Run Time:	10.0 Minutes	Proc. Chnl. Descr.:	PDA 250.0 nm
Date Acquired:	12/5/2014 5:16:08 PM GMT		
Date Processed:	1/8/2015 2:56:19 PM GMT		

Auto-Scaled Chromatogram

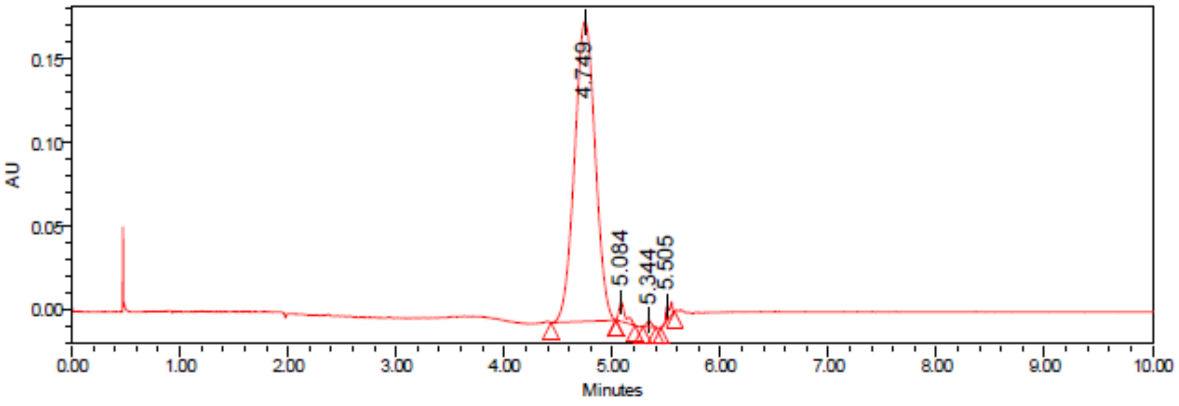

Peak Results

	Name	RT	Area	Height	% Area
1		4.749	2387605	179666	96.53
2		5.084	47850	11614	1.93
3		5.344	11664	3742	0.47
4		5.505	26353	8482	1.07

Compound (1*R*)-8-lac

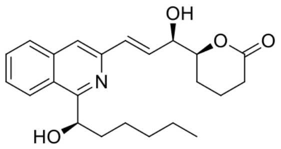

97 % purity at 230 nm

Sample Name:	DM-RA3	Acquired By:	System
Sample Type:	Unknown	Sample Set Name:	DM_RA3_gen1
Vial:	1:E,8	Acq. Method Set:	generic_chiral_sol1
Injection #:	1	Processing Method	DM164_A
Injection Volume:	10.00 ul	Channel Name:	230.0nm
Run Time:	7.0 Minutes	Proc. Chnl. Descr.:	PDA 230.0 nm
Date Acquired:	3/16/2015 6:04:38 PM GMT		
Date Processed:	1/6/2016 11:27:11 AM GMT		

Auto-Scaled Chromatogram

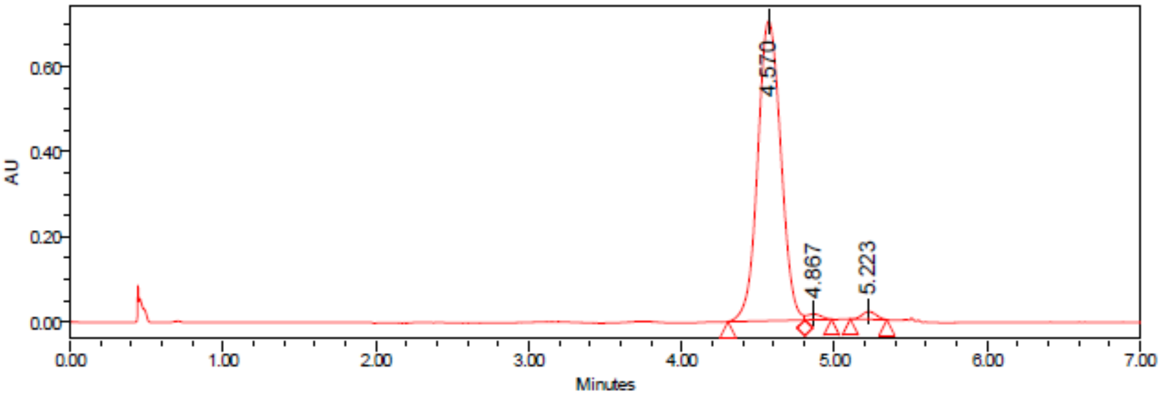

Peak Results

	Name	RT	Area	Height	% Area
1		4.570	7400115	704722	97.35
2		4.867	87697	13254	1.15
3		5.223	113814	18193	1.50

Compound (1*R*)-8-lac

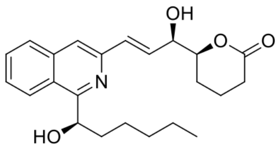

97 % purity at 250 nm

Sample Name:	DM-RA3	Acquired By:	System
Sample Type:	Unknown	Sample Set Name:	DM_RA3_gen1
Vial:	1:E,8	Acq. Method Set:	generic_chiral_sol1
Injection #:	1	Processing Method	DM
Injection Volume:	10.00 ul	Channel Name:	250.0nm
Run Time:	7.0 Minutes	Proc. Chnl. Descr.:	PDA 250.0 nm
Date Acquired:	3/16/2015 6:04:38 PM GMT		
Date Processed:	1/6/2016 11:31:08 AM GMT		

Auto-Scaled Chromatogram

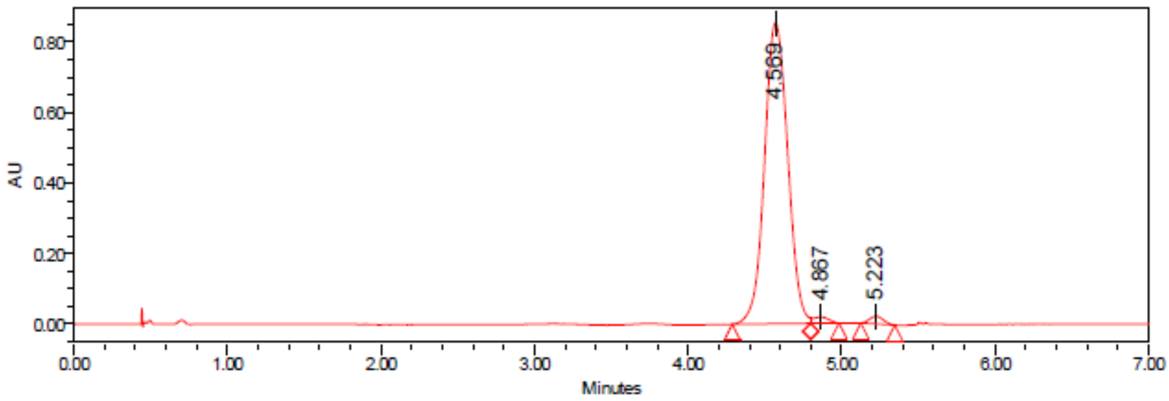

Peak Results

	Name	RT	Area	Height	% Area
1		4.569	8964171	852940	97.18
2		4.867	125032	17671	1.36
3		5.223	134756	21896	1.46

Compound (1*S*)-8

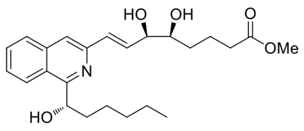

95 % purity at 285 nm

Sample Name	: DM162_SB3_90.10		
Sample ID	:		
Data Filename	: DM162_SB3-90.10_DM162_SB3_90.10_005.lcd		
Method Filename	: Isocratic-90_10_1.0ml_40min.lcm		
Batch Filename	: DM162_SB3-90.10.lcb		
Vial #	: 1-79	Sample Type	: Unknown
Injection Volume	: 2 uL		
Date Acquired	: 10/23/2014 3:58:27 PM	Acquired by	: System Administrator
Date Processed	: 10/23/2014 5:11:28 PM	Processed by	: System Administrator

<Chromatogram>

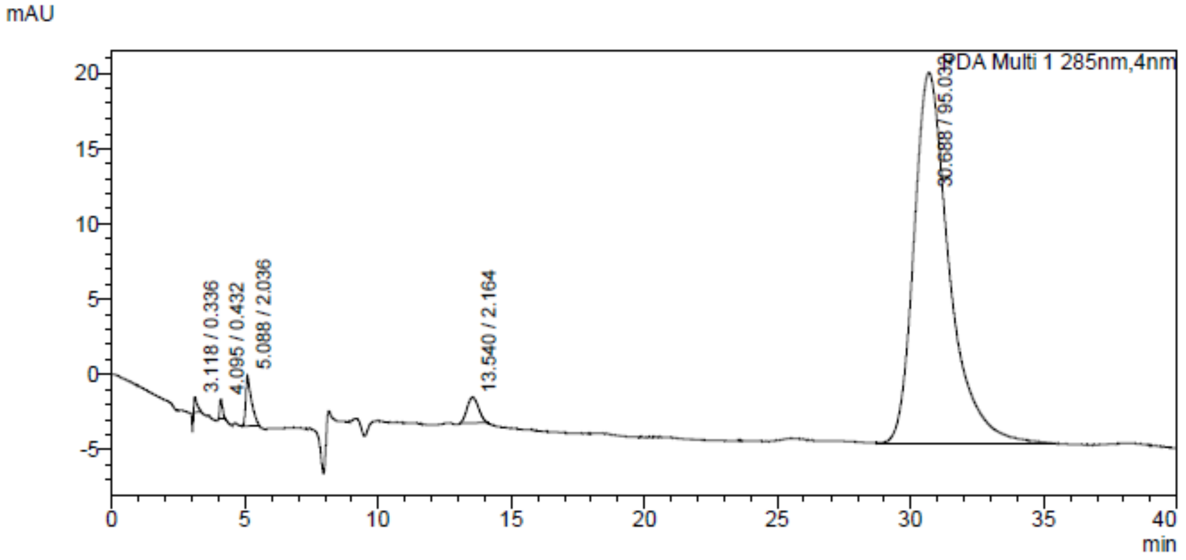

<Peak Table>

PDA Ch1 285nm

Peak#	Ret. Time	Area	Area%	Height
1	3.118	8023	0.336	1013
2	4.095	10327	0.432	1317
3	5.088	48653	2.036	3366
4	13.540	51717	2.164	1717
5	30.688	2270956	95.032	24601
Total		2389676	100.000	32014

Compound (1*S*)-8

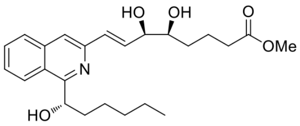

95 % purity at 300 nm

Sample Name	: DM162_SB3_90.10		
Sample ID	:		
Data Filename	: DM162_SB3-90.10_DM162_SB3_90.10_005.lcd		
Method Filename	: Isocratic-90_10_1.0ml_40min.lcm		
Batch Filename	: DM162_SB3-90.10.lcb		
Vial #	: 1-79	Sample Type	: Unknown
Injection Volume	: 2 uL		
Date Acquired	: 10/23/2014 3:58:27 PM	Acquired by	: System Administrator
Date Processed	: 10/23/2014 5:11:28 PM	Processed by	: System Administrator

<Chromatogram>

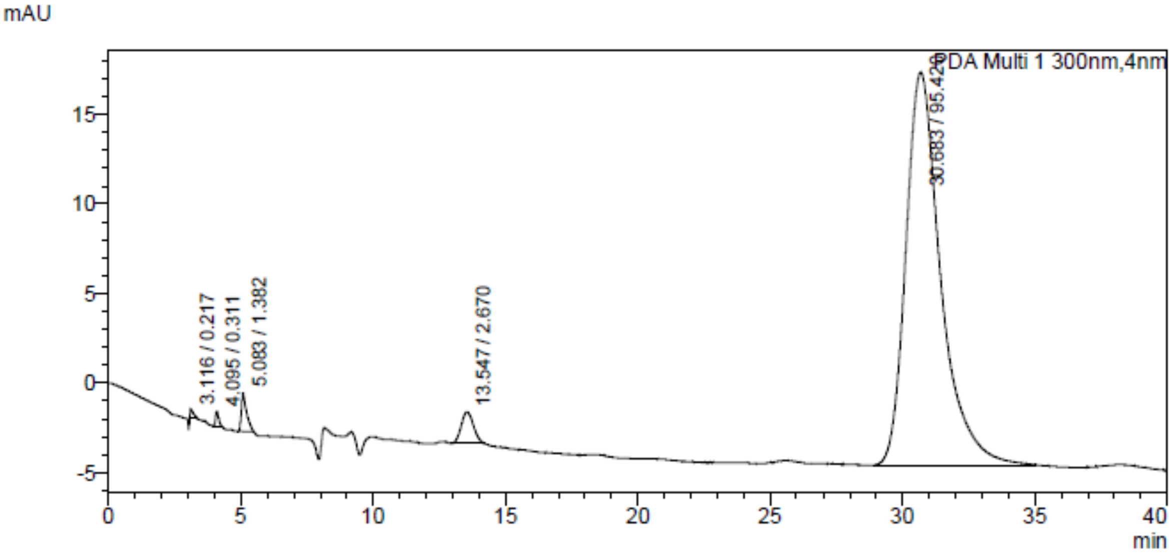

<Peak Table>

PDA Ch1 300nm

Peak#	Ret. Time	Area	Area%	Height
1	3.116	4582	0.217	521
2	4.095	6553	0.311	806
3	5.083	29119	1.382	2181
4	13.547	56248	2.670	1765
5	30.683	2010553	95.420	21948
Total		2107055	100.000	27222

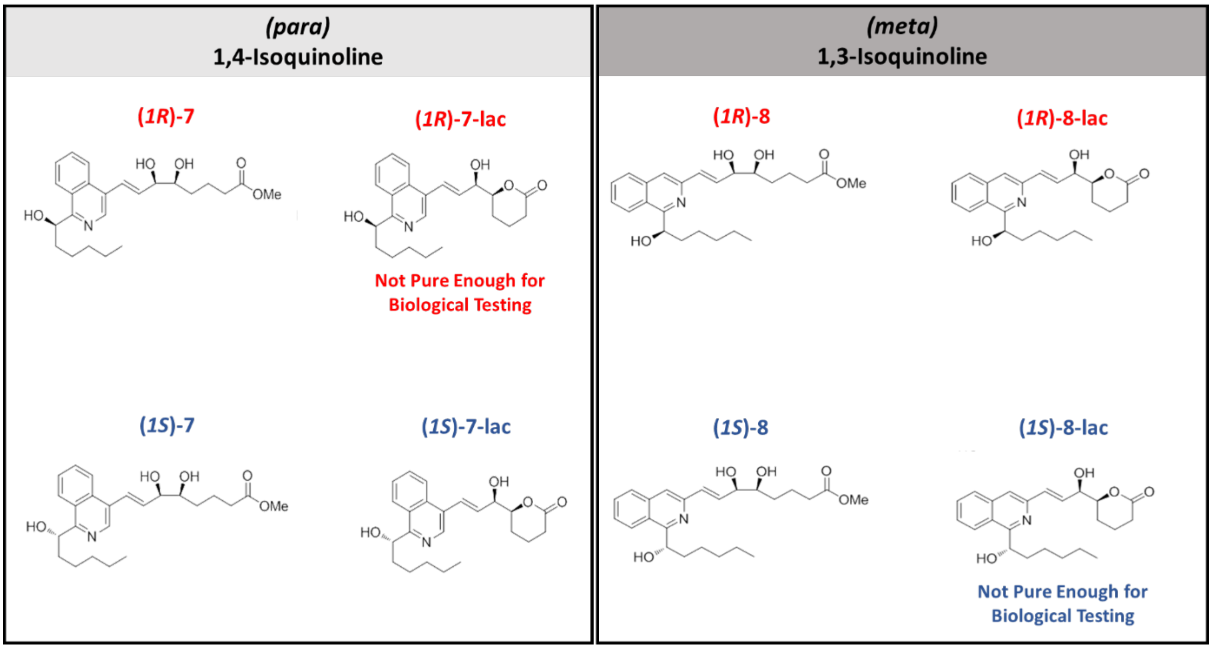

Supplementary Figure 1 - Rational Design of SAR Analysis of Isoquinoline-containing LXA4 mimetics. Chemical structures of the 8 compounds synthesised to mimic the actions of the native LXA4. Addition of the heterocyclic aromatic organic substituent (Isoquinoline) in position *para* (1,4) or *meta* (1,3), followed by the addition of an esterified methyl group or a lactone (lac) ring to C alpha, were performed to improve the stability/activity of LXA4. Both (*R*)- and (*S*)-enantiomers were also included in testing for stringency. (1*R*)-7-lac and (1*S*)-8-lac were determined to not be pure enough for biological testing (purity <95% *via* HPLC).

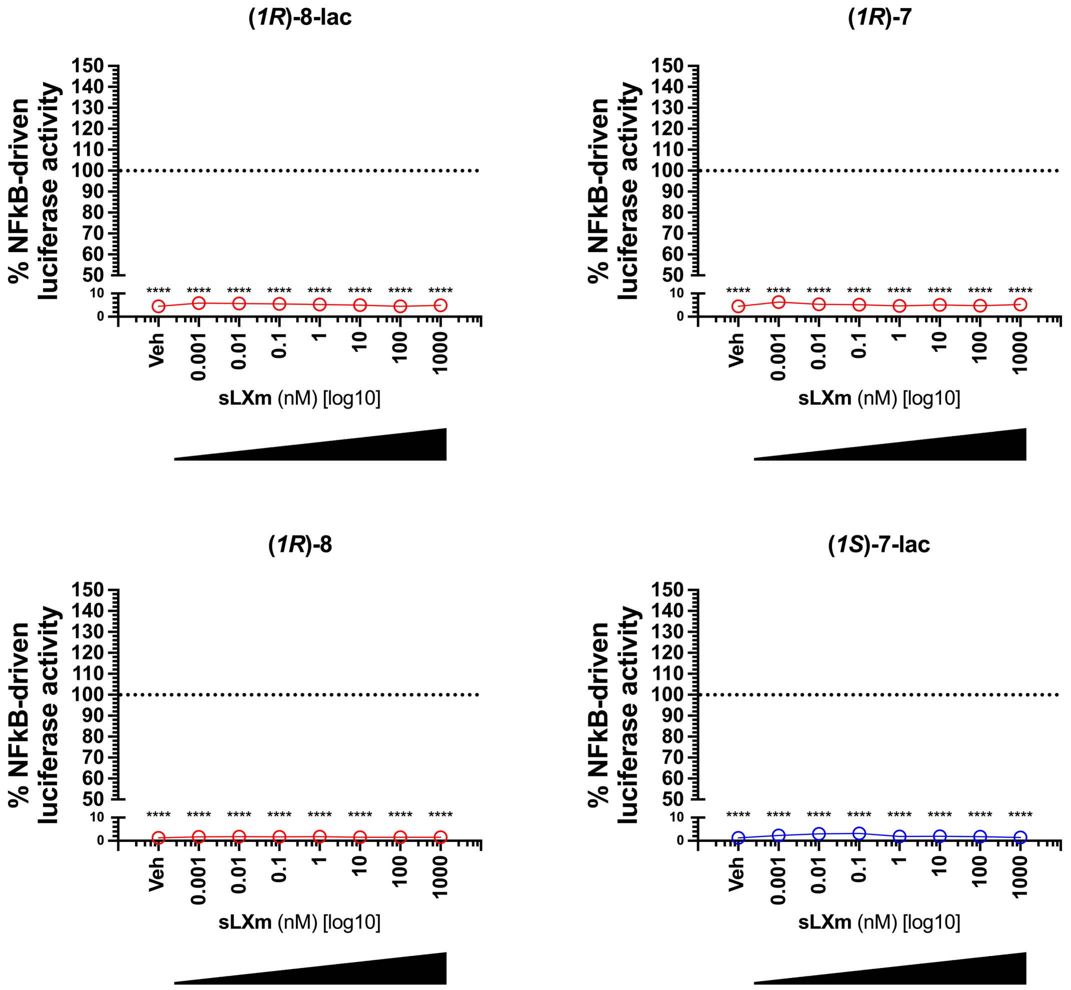

Supplementary Figure 2 - Representative effect of Isoquinoline-containing LXA4 mimetics on intrinsic NF*k*B activity of THP-1 LUCIA monocytes. 1 x10^5 THP-1 LUCIA monocytes were treated for 24 h with increasing concentrations of the isoquinoline-containing sLXms (10-12 M / 1 pM – 10-6 M / 1000 nM), vehicle, or appropriate controls, in the **absence** of LPS stimulation. After 24 h, cell supernatants were collected, and NF-kB luciferase activity was assayed. Data are expressed as a % ± SEM (*n* = 3) of NF-kB-driven luciferase activity (dotted line at 100% response) as normalised luminescence unit relative to an LPS-induced response in the absence of sLXms. Concentration-response curve displayed are comparing the epimers of four representative sLXms. Statistical analyses performed using Student's unpaired two-tailed *t-test* of the tested compounds *vs* LPS (*p <0.05; **p<0.01; ***p<0.001; ****p,0.0001).

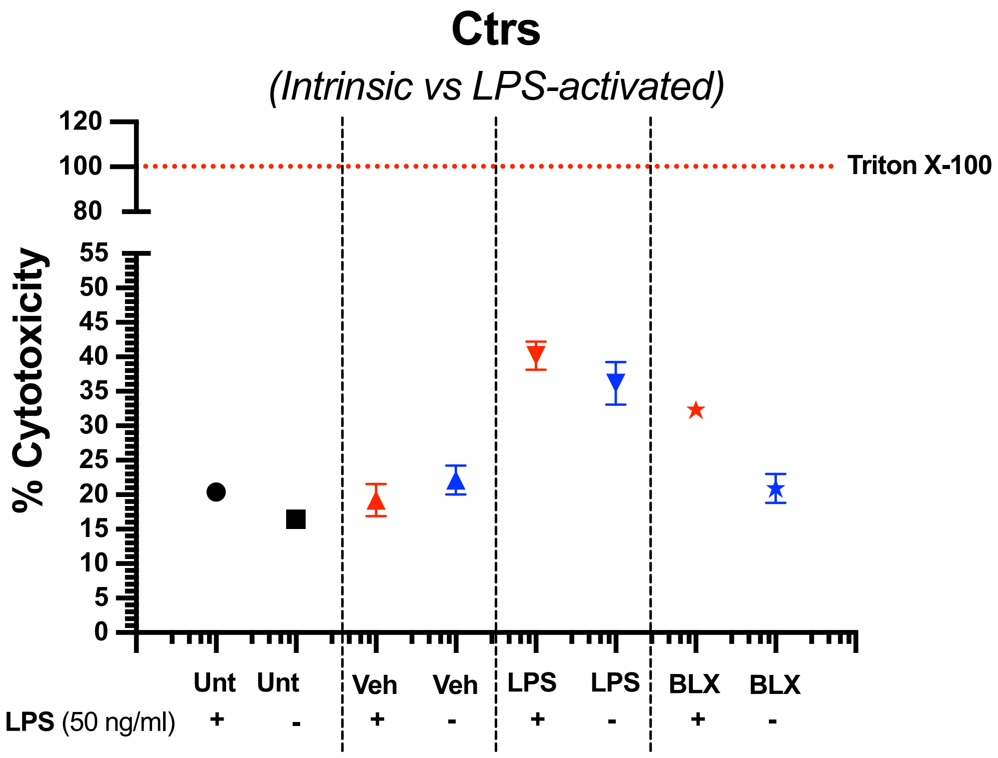

Supplementary Figure 3. Intrinsic/Extrinsic Cytotoxicity Profile of controls. 1 x 10^5 THP-1 LUCIA monocytes were pre-treated for 30 min. with vehicle, or the appropriate controls. This was followed by the addition of 50 ng/mL LPS (extrinsic response)or the absence of it (intrinsic response) . After 24 h, LDH release was analysed and expressed as a percentage of cytotoxicity. Concentration-curve both with LPS stimulus (blue) and without LPS (red). Triton X-100 represents maximal LDH release from cells and has been set to 100%, allowing for benchmarks to be set at spontaneous LDH release (red dotted line). Data expressed as %Triton X-100 ± SEM (n = 3). Data analysis performed as One-way ANOVA with Dunnett's multiple comparisons test comparing reduction of LDH compared to LPS LDH release, where p<0.05 for LPS-treated samples is annotated as *, and p<0.05 is annotated by § for non-LPS-treated samples.
